# Supplementary material for: Distributing aminophospholipids asymmetrically across leaflets causes anomalous membrane stiffening
Source: Biophys J. 2023 Apr 29;122(12):2445–55. doi: 10.1016/j.bpj.2023.04.025 (PMC10322881; doi:10.1016/j.bpj.2023.04.025)
Supplement: Document S1. Figures S1–S16 and Tables S1–S8 [file mmc1.pdf]

Biophysical Journal, Volume *122*

Supplemental information

Distributing aminophospholipids asymmetrically across leaflets causes anomalous membrane stiffening

Moritz P.K. Frewein, Paulina Piller, Enrico F. Semeraro, Orsolya Czakkel, Yuri Gerelli, Lionel Porcar, and Georg Pabst

Supplement: Aminophospholipids Stiffen Membranes When Populating Their Inner Leaflets

Moritz PK Frewein, Paulina Piller, Enrico F Semeraro,
Orsolya Czakkel, Yuri Gerelli, Lionel Porcar and Georg Pabst

Contents

1 Effect of hypotonic conditions on membrane tension **2**

2 Evaluation of NMR data **3**

3 SDP-model used for SAS-analysis **4**

4 The contribution of diffusion in NSE-data modelling **7**

5 Bending Rigidities of Lipid mixtures **8**

6 SAS-model parameters of donor/acceptor lipids **9**

7 SAS-model parameters of asymmetric vesicles **11**

8 SAS-model parameters of reference LUVs **16**

1 Effect of hypotonic conditions on membrane tension

Preparation of acceptor vesicles in 25 mM NaCl and washing in pure water after CD-mediated lipid exchange creates an osmotic imbalance between the lumen of the aLUVs and the bulk water solution, which potentially leads to a surface tension that affects bending rigidity measurements. In the following, we estimate this effect.

Lipid vesicles are known to swell under hypotonic conditions until reaching a critical tension beyond which pores or defects may form to relax the stress [1]. At lower tension and in equilibrium, the osmotic pressure created by the osmolytes

$$P_{osm} = kTN_A\Delta c_s \tag{1}$$

balances the Laplace pressure

$$P_L = 2\sigma/R, \tag{2}$$

where k is Boltzmann's constant, N_A is Avogadro's constant, Δc_s is the concentration difference of the solute between the lumen of the vesicle and bulk solution, σ is the surface tension and R is the vesicle radius. The change in membrane tension due to swelling of the vesicle is easily calculated from the change of vesicle size.

$$\Delta\sigma = \frac{kTN_A\Delta c_s}{2}\Delta R. \tag{3}$$

We observed an increase of vesicle radii between 0 and 18 nm (Tab. S1) by DLS. The highest value appears to be an outlier due to the presence of a minor population of donor vesicles; see also (Fig. S8). Thus, using $\Delta c_s = 25$ mM and $\Delta R = 3$ nm (average size increase, excluding the outlier) we calculate $\Delta\sigma \approx 10^{-4}$ mN/m.

We can estimate the effect of the calculated membrane tension change on the membrane thickness, d_B. Molecular dynamics simulations of dioleoyl phosphatidylcholine reported $\frac{\Delta d_B}{\Delta\sigma} \approx -1.33 \times 10^3$ Å/(mN/m) [2]. The expected change of membrane thickness for $\Delta\sigma \approx 10^{-4}$ mN/m, therefore, is ≈ -0.1 Å, i.e. within our present experimental uncertainty of SAXS/SANS (consistent with our observations). Membrane tension does affect the undulation amplitudes of vesicles, which might couple for sufficiently high values to the bending rigidity, κ [3]. However, the calculated tension change is well within the low-tension regime (< 0.5 mN/m), where κ remains constant and enables its measurement via micropipette pressurization of giant bilayer vesicles [4]. Thus, we can neglect any systematic increase of bending rigidity originating from our aLUV preparation.

Table S1: Vesicle radii for acceptor vesicles, R_{av}^{acc}, and aLUVs, R_{av}^{asym} from DLS experiments (experimental uncertainty: ±3 nm). Experiments and data analysis were done using a Malvern Zetasizer Nano ZS90 and the software supplied by the manufacturer (Malvern Panalytical, Malvern, UK). PDI denotes the polydispersity index of the measured size distribution.

Sample	R_{av}^{acc} [nm]	PDI [%]	R_{av}^{asym} [nm]	PDI [%]	ΔR [nm]
$POPE^{in}/ESM^{out}$	61	6	63	11	2
$POPE^{in}/MSM^{out}$	55	10	58	18	3
$POPE^{in}/POPC^{out}$	55	10	64	30	9
$POPE^{in}/(POPC/MSM)^{out}$	55	10	58	17	3
$(POPE/POPS)^{in}/MSM^{out}$	65	15	65	6	0
$(POPE/POPS)^{in}/POPC^{out}$	65	15	83	30	18[a]
$(POPE/POPS)^{in}/(POPC/MSM)^{out}$	65	15	68	13	3

[a] minor donor contamination (see also Fig. S8)

2 Evaluation of NMR data

Figure S1 shows an exemplary NMR spectrum of asymmetric vesicles before and after addition of Pr^{3+}. The areas A_0 and A_1 under the Lorentzian fits of the peak at 3.4 ppm (dotted lines) correspond to the total amount of choline headgroups (before) and the amount of choline lipids inside the vesicle (after), respectively. The mol-fraction of PC-lipids (POPC and/or Sphingomyelin) in the inner leaflet is therefore given by $\chi_P^{in} = A_1/A_0$.

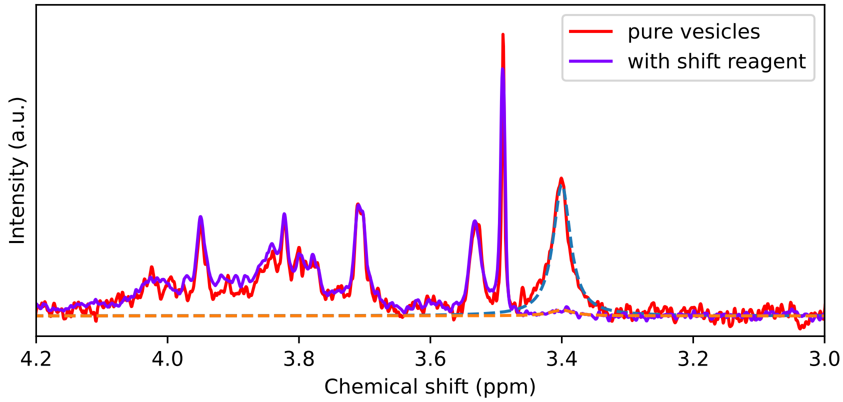

Figure S1: The graph shows a part of the NMR spectrum of asymmetric vesicles before and after adding Pr^{3+}. While the majority of the choline peak is moved from its original position (3.4 ppm), the rest of the spectrum is unaffected. Dotted lines mark Lorentzians used to fit the peaks and to calculate the underlying areas.

3 SDP-model used for SAS-analysis

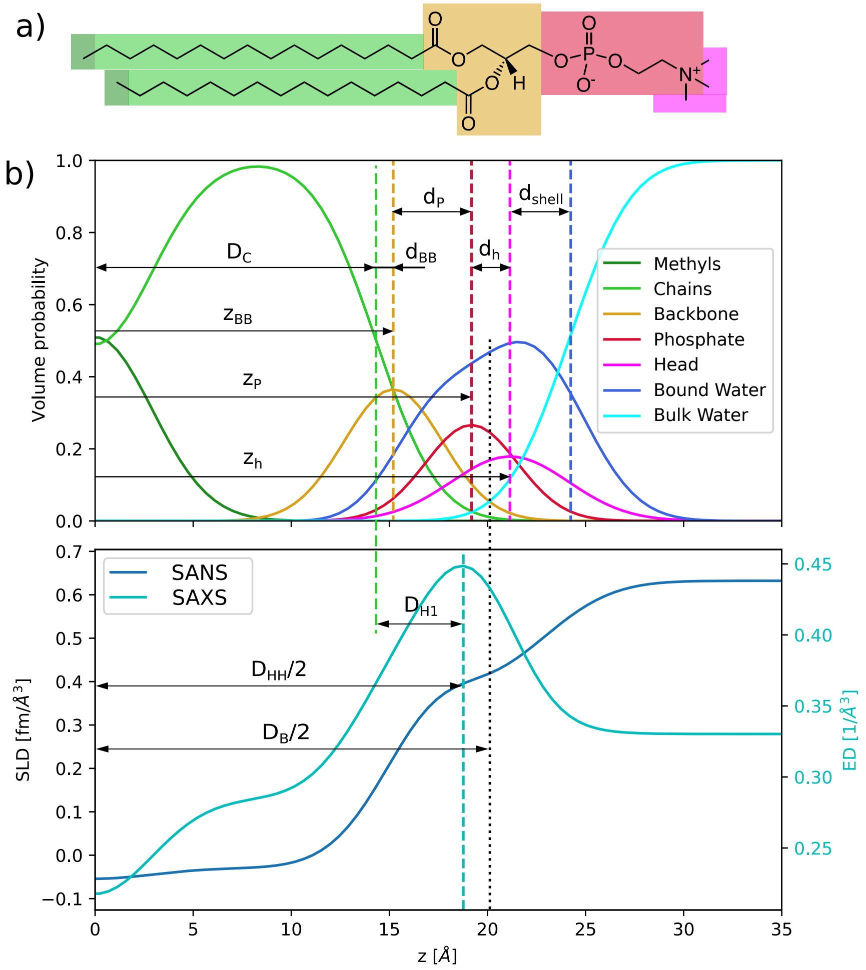

Figure S2: Lipid parsing and scattering length density profile modelling: a) The upper graphic shows how a lipid is divided in fragments on the example of DPPC. The parsing off all other used lipids can be found in Table S2. b) The color-coded arrangement of functions (*pdf*) in the upper diagram corresponds to the probability density to find a fragment at a distance z from the bilayer center. The *pdf*s for the terminal methyls (T), lipid backbones (BB), phosphate groups (P) and heads (h) are modelled by Gaussian functions. Hydrocarbon chains (HC) and a hydration water layer (bound water, BW) are slabs with altitude 1 and smeared with error-functions. From these, the functions of T and $BB + P + H$ are substracted respectively. The *pdf* of the solvent (bulk water W) fills the remaining space, so that the sum of all *pdf*s is 1 at all z. The lower diagram shows the neutron scattering length density (SLD) and electron density (ED) profiles of the modelled lipid in D_2O. It is obtained from the sum over all *pdf*s, multiplied with the respective SLD or ED.

Table S2: List of lipid fragments and chemical compositions for all used lipids. X denote exchangable hydrogens, which are replaced with deuterium in a D_2O-environment.

Fragment	Terminal methyls	Chains without T	Backbone	Phosphate group	Head
Abbr.	T	HC	BB	P	h
DPPC	$(CH_3)_2$	$(CH_2)_{28}$	$C_5O_4H_5$	$PO_4(CH_2)_2N$	$(CH_3)_3$
DPPG	$(CH_3)_2$	$(CH_2)_{28}$	$C_5O_4H_5$	PO_4	$C_3H_5O_2X_2$
POPE	$(CH_3)_2$	$(CH_2)_{28}(CH)_2$	$C_5O_4H_5$	PO_4	$(CH_2)_2NX_3$
POPG	$(CH_3)_2$	$(CH_2)_{28}(CH)_2$	$C_5O_4H_5$	PO_4	$C_3H_5O_2X_2$
POPS	$(CH_3)_2$	$(CH_2)_{28}(CH)_2$	$C_5O_4H_5$	PO_4	$C_3H_2X_3NO_2H$
POPC	$(CH_3)_2$	$(CH_2)_{28}(CH)_2$	$C_5O_4H_5$	$PO_4(CH_2)_2N$	$(CH_3)_3$
ESM	$(CH_3)_2$	$(CH_2)_{26.6}(CH)_2$	$C_4O_2NH_6$	$PO_4(CH_2)_2N$	$(CH_3)_3$
MSM	$(CH_3)_2$	$(CH_2)_{32}(CH)_2$	$C_4O_2NH_6$	$PO_4(CH_2)_2N$	$(CH_3)_3$

SAS-data are modelled using the vesicle form factor F_{sphere}, the bilayer form F_{bil}, which we split into real and imaginary part, and the incoherent background I_{inc}. Furthermore, we applied a Gaussian polydispersity on the hydrophobic thickness D_C, which results in a weighted average over several bilayer form factors $F_{bil,k}$. A more detailed description of the model was published earlier [5]. Due to the lack of contrast between both leaflets, we are not able to locate the bilayer center by SANS and therefore fixed the position of the terminal methyl group $z_T = 0$ in this study. For all symmetric references, the imaginary part of the bilayer form factor is equal 0 and only one leaflet has to be modelled. All quantities $\Delta\rho$ denote the scattering length density contrast of moiety k with respect to the water/heavy water environment $\Delta\rho_k = \rho_k - \rho_W$. Distances are defined in Fig. S2, where D_B is defined as the Luzzati bilayer thickness and D_{HH} the distance between the maxima in the ED-profile. $D_{H1} = (D_B - D_{HH})/2$. σ_k are the standard deviations of the respective *pdf*s. d_{shell} was fixed to 3.1 Å for all samples. Further parameters: A area per lipid; V_L total lipid volume; V_H total head group volume (back bone, phosphate group and head); $r_{BB} = V_{BB}/V_H$, $r_P = V_P/V_H$, $r = V_{CH3}/V_{CH2}$, $r_{12} = V_{CH}/V_{CH2}$; $V_{W,bound}$ volume per bound water molecule; n_W number of bound water molecules; Y relative interdigitation (see [6]).

$$I(q) \propto F_{sphere}(R_m, \sigma_R) \sum_k \mathcal{N}(D_{C,k}|\bar{D_C}, \sigma_{\text{poly}}) \left\{ |F^{real}_{bil,k}|^2 + |F^{imag}_{bil,k}|^2 | \right\} + I_{inc} \tag{4}$$

with

$$\begin{aligned} F^{real}_{bil,k} =& 4\Delta\rho_{\text{bw}} \frac{1}{q} \mathrm{e}^{-\frac{q^2 \sigma^{\text{in}\,2}_{\text{CH2}}}{2}} \sin\left(q \frac{d^{\text{in}}_{\text{BB}} + d^{\text{in}}_P + d^{\text{in}}_{shell}}{2} \right) \\ & \cos\left(q(-D^{\text{in}}_{C,k} - \frac{d^{\text{in}}_{\text{BB}} + d^{\text{in}}_P d^{\text{in}}_{shell}}{2}) \right) + \\ & 2(\Delta\rho^{\text{in}}_P - \Delta\rho_{\text{bw}}) \frac{V^{in}_P}{A} \mathrm{e}^{-\frac{q^2 \sigma^{\text{in}\,2}_P}{2}} \cos\left(q(-D^{\text{in}}_{C,k} - d^{\text{in}}_{\text{BB}} - d^{\text{in}}_P/2) \right) + \\ & 2(\Delta\rho^{\text{in}}_{\text{BB}} - \Delta\rho_{\text{bw}}) \frac{V^{\text{in}}_{\text{BB}}}{A} \mathrm{e}^{-\frac{q^2 \sigma^{\text{in}\,2}_{\text{BB}}}{2}} \cos\left(q(-D^{\text{in}}_{C,k} - d^{\text{in}}_{\text{BB}}/2) \right) + \\ & 2\Delta\rho^{\text{in}}_{\text{CH2}} \frac{1}{q} \mathrm{e}^{-\frac{q^2 \sigma^{\text{in}\,2}_{\text{CH2}}}{2}} \sin\left(q D^{\text{in}}_{C,k}/2 \right) \cos\left(q(-D^{\text{in}}_{C,k}/2) \right) + \\ & 2\Delta\rho_{\text{CH2}} \frac{1}{q} \mathrm{e}^{-\frac{q^2 \sigma^2_{\text{CH2}}}{2}} \sin\left(q D^{\text{out}}_{C,k}/2 \right) \cos\left(q D^{\text{out}}_{C,k}/2 \right) + \\ & 2(\Delta\rho^{\text{out}}_{\text{BB}} - \Delta\rho_{\text{bw}}) \frac{V^{\text{out}}_{\text{BB}}}{A} \mathrm{e}^{-\frac{q^2 \sigma^{\text{out}\,2}_{\text{BB}}}{2}} \cos\left(q(D^{\text{out}}_{C,k} + d^{\text{out}}_{\text{BB}}/2) \right) + \\ & 2(\Delta\rho^{\text{out}}_P - \Delta\rho_{\text{bw}}) \frac{V^{\text{out}}_P}{A} \mathrm{e}^{-\frac{q^2 \sigma^{\text{out}\,2}_P}{2}} \cos\left(q(D^{\text{out}}_{C,k} + d^{\text{out}}_{\text{BB}} + d^{\text{out}}_P/2) \right) + \\ & 4\Delta\rho_{\text{bw}} \frac{1}{q} \mathrm{e}^{-\frac{q^2 \sigma^{\text{out}\,2}_{\text{CH2}}}{2}} \sin\left(q \frac{d^{\text{out}}_{\text{BB}} + d^{\text{out}}_P + d^{\text{out}}_{shell}}{2} \right) \\ & \cos\left(q(D^{\text{out}}_{C,k} + \frac{d^{\text{out}}_{\text{BB}} + d^{\text{out}}_P + d^{\text{out}}_{shell}}{2}) \right) \end{aligned} \tag{5}$$

and

$$
\begin{aligned}
F_{bil,k}^{imag} =& 4\Delta\rho_{\mathrm{bw}}\frac{1}{q}\mathrm{e}^{-\frac{q^2\sigma_{\mathrm{CH2}}^{\mathrm{in}\,2}}{2}}\sin\left(q\frac{d_{\mathrm{BB}}^{\mathrm{in}}+d_{P}^{\mathrm{in}}+d_{shell}^{\mathrm{in}}}{2}\right) \\
& \sin\left(q(-D_{C,k}^{\mathrm{in}}-\frac{d_{\mathrm{BB}}^{\mathrm{in}}+d_{P}^{\mathrm{in}}d_{shell}^{\mathrm{in}}}{2})\right)+ \\
& 2(\Delta\rho_{P}^{\mathrm{in}}-\Delta\rho_{\mathrm{bw}})\frac{V_{P}^{in}}{A}\mathrm{e}^{-\frac{q^2\sigma_{P}^{\mathrm{in}\,2}}{2}}\sin\left(q(-D_{C,k}^{\mathrm{in}}-d_{\mathrm{BB}}^{\mathrm{in}}-d_{P}^{\mathrm{in}}/2)\right)+ \\
& 2(\Delta\rho_{\mathrm{BB}}^{\mathrm{in}}-\Delta\rho_{\mathrm{bw}})\frac{V_{\mathrm{BB}}^{\mathrm{in}}}{A}\mathrm{e}^{-\frac{q^2\sigma_{\mathrm{BB}}^{\mathrm{in}\,2}}{2}}\sin\left(q(-D_{C,k}^{\mathrm{in}}-d_{\mathrm{BB}}^{\mathrm{in}}/2)\right)+ \\
& 2\Delta\rho_{\mathrm{CH2}}^{\mathrm{in}}\frac{1}{q}\mathrm{e}^{-\frac{q^2\sigma_{\mathrm{CH2}}^{\mathrm{in}\,2}}{2}}\sin\left(qD_{C,k}^{\mathrm{in}}/2\right)\sin\left(q(-D_{C,k}^{\mathrm{in}}/2\right)+ \\
& \frac{\Delta\rho_{\mathrm{T}}^{\mathrm{in}}+\Delta\rho_{\mathrm{T}}^{\mathrm{out}}}{2}\mathrm{e}^{-\frac{q^2\sigma_{\mathrm{T}}^2}{2}}+ \\
& 2\Delta\rho_{\mathrm{CH2}}\frac{1}{q}\mathrm{e}^{-\frac{q^2\sigma_{\mathrm{CH2}}^2}{2}}\sin\left(qD_{C,k}^{\mathrm{out}}/2\right)\sin\left(qD_{C,k}^{\mathrm{out}}/2\right)+ \\
& 2(\Delta\rho_{\mathrm{BB}}^{\mathrm{out}}-\Delta\rho_{\mathrm{bw}})\frac{V_{\mathrm{BB}}^{\mathrm{out}}}{A}\mathrm{e}^{-\frac{q^2\sigma_{\mathrm{BB}}^{\mathrm{out}\,2}}{2}}\sin\left(q(D_{C,k}^{\mathrm{out}}+d_{\mathrm{BB}}^{\mathrm{out}}/2)\right)+ \\
& 2(\Delta\rho_{P}^{\mathrm{out}}-\Delta\rho_{\mathrm{bw}})\frac{V_{P}^{\mathrm{out}}}{A}\mathrm{e}^{-\frac{q^2\sigma_{P}^{\mathrm{out}\,2}}{2}}\sin\left(q(D_{C,k}^{\mathrm{out}}+d_{\mathrm{BB}}^{\mathrm{out}}+d_{P}^{\mathrm{out}}/2)\right)+ \\
& 4\Delta\rho_{\mathrm{bw}}\frac{1}{q}\mathrm{e}^{-\frac{q^2\sigma_{\mathrm{CH2}}^{\mathrm{out}\,2}}{2}}\sin\left(q\frac{d_{\mathrm{BB}}^{\mathrm{out}}+d_{P}^{\mathrm{out}}+d_{shell}^{\mathrm{out}}}{2}\right) \\
& \sin\left(q(D_{C,k}^{\mathrm{out}}+\frac{d_{\mathrm{BB}}^{\mathrm{out}}+d_{P}^{\mathrm{out}}+d_{shell}^{\mathrm{out}}}{2})\right)
\end{aligned} \tag{6}
$$

To describe the contribution from the overall vesicle shape we use the Schultz-distributed form factor of a sphere, as described in [7]:

$$
F_{sphere} = \frac{8\pi^2(z+1)(z+2)}{s^2q^2}\left\{1-\left(1+\frac{4q^2}{s^2}\right)^{-(z+3)/2}\cos\left[(z+3)\arctan\left(\frac{2q}{s}\right)\right]\right\} \tag{7}
$$

Mean vesicle radius R_m and polydispersity σ_R enter via the auxiliary quantities $s=\frac{R_m}{\sigma_R^2}$ and $z=\frac{R_m^2}{\sigma_R^2}-1$.

4 The contribution of diffusion in NSE-data modelling

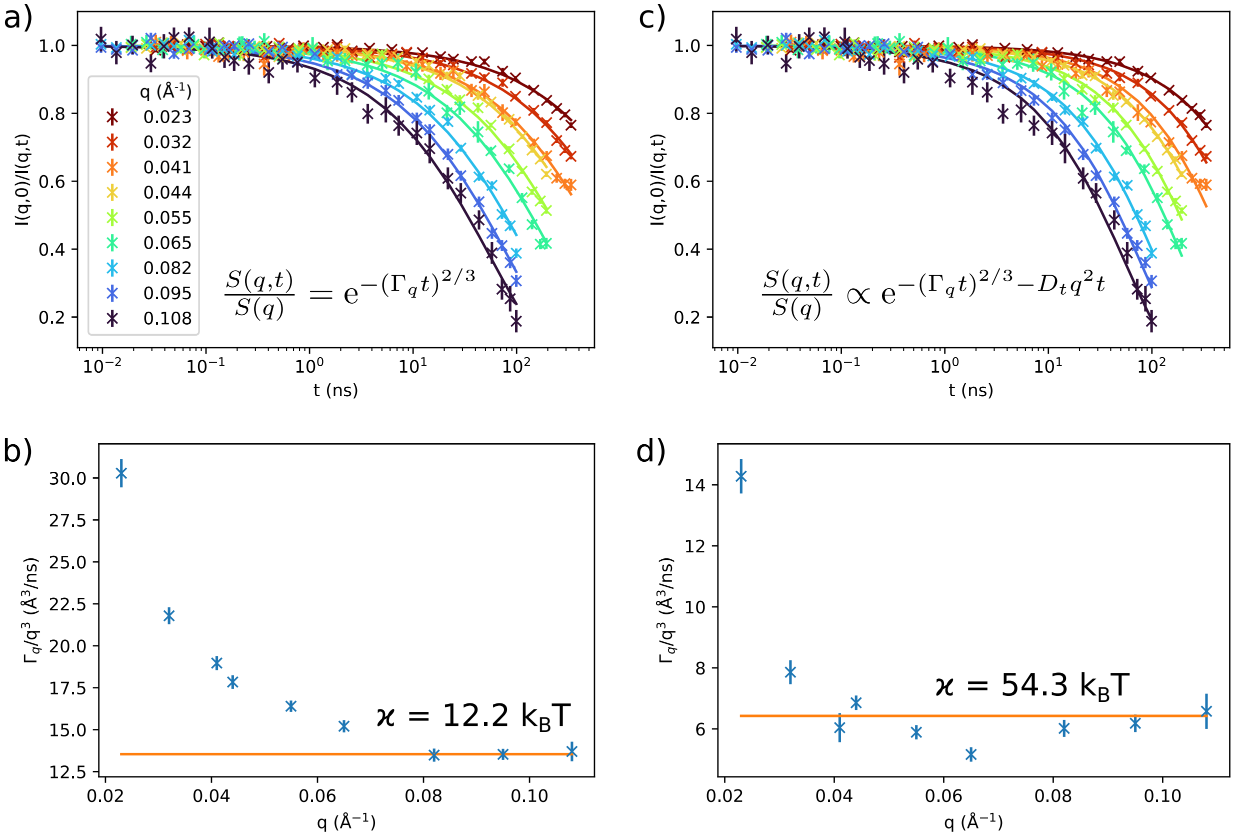

Figure S3: The graphic shows the comparison of 2 models used to evaluate NSE-data of DPPC-vesicles at 50 °C: (I) the pure Zilman-Granek (ZG) model (a,b) and (II) the ZG model with a contribution from diffusion (c,d), using a translational diffusion constant $D_t = 0.66$ Å^2/ns, measured by dynamic light scattering. The formulas to fit data are given in (a) and (c). Both models are in reasonable agreement with the raw data, however using model (I), the resulting values for the q-dependent decay constant Γ_q (b) do not follow q^3 as predicted by the ZG-theory. Model (II) improves the agreement over a wider q-range (d), but still deviates at low q. The given values for the bending rigidity κ in (b) and (d) correspond to the orange lines.

5 Bending Rigidities of Lipid mixtures

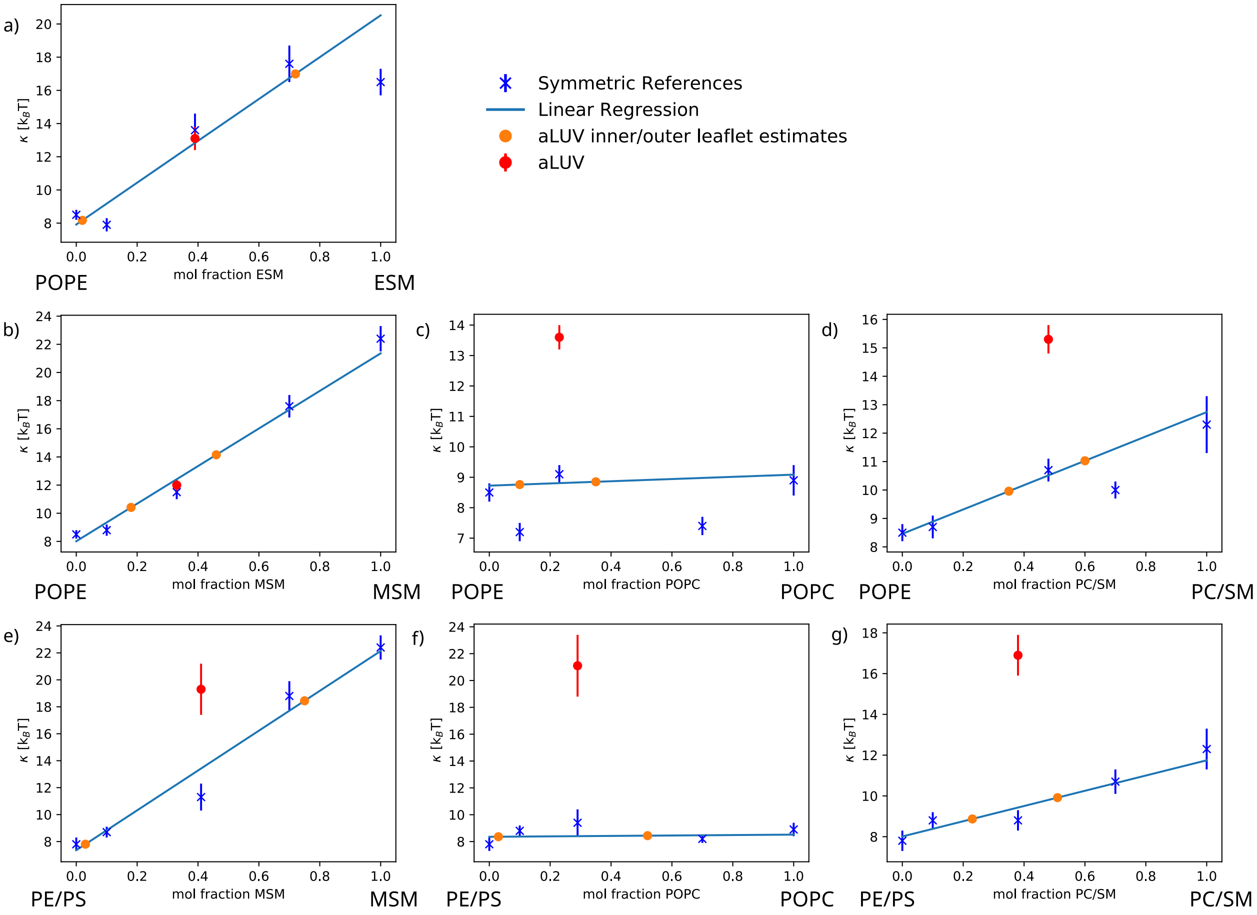

Figure S4: The graphic shows the measured bending rigidities κ for all aLUVs (red) as well as symmetric references (blue). We performed linear regressions through the reference samples and extrapolated to retrieve the κ at the measured inner/outer leaflet compositions (orange). Some points had to be excluded from the analysis due to large deviations from the linear law: a) $X_{ESM} = 0.1$ and $X_{ESM} = 1$, c) $X_{POPC} = 0.1$ and $X_{POPC} = 0.7$, d) $X_{PC/SM} = 0.7$, e) $X_{MSM} = 0.46$. The origin of these deviations is unclear.

6 SAS-model parameters of donor/acceptor lipids

Table S3: Summary of structural properties of symmetric LUVs at 50°C.

	A (Å^2)	D_{HH} (Å)[c]	$2D_C$ (Å)	h (Å)
DPPC[a]	63.1 ± 1.3	37.5 ± 1.1	28.6 ± 0.9	15.2 ± 1.2
POPC[a]	67.5 ± 1.4	37.5 ± 1.1	28.4 ± 0.9	14.8 ± 1.2
MSM[a]	64.8 ± 1.3	43.0 ± 1.1	32.8 ± 1.0	18.4 ± 1.5
ESM	56.2 ± 1.1	41.5 ± 1.1	33.6 ± 1.0	19.8 ± 1.6
POPE	60.5 ± 1.2	36.0 ± 1.1	31.2 ± 0.9	17.0 ± 1.4
POPE/POPS[b]	65.3 ± 1.3	37.1 ± 1.1	28.9 ± 0.9	17.0 ± 1.4

[a] Structural data taken from ref. [6].
[b] 7:3 mol/mol.
[c] head-to-headgroup distance.

Table S4: Properties of symmetric lipid bilayers from SAXS/SANS analysis at 50 °C containing POPE/POPG 9:1 (POPE), POPE/POPS 7:3 (PE/PS) and ESM/DPPG 19:1 (ESM).

	ϵ [%]	POPE	PE/PS	ESM
V_L^* [Å^3]		1193.8	1199.3	1218.5
V_H^* [Å^3]		249.6	254.9	274.9
r_{BB}^*		0.51	0.50	0.33
r_P^*		0.14	0.18	0.31
r^*		2.09	2.09	2.09
r_{12}^*		0.8	0.8	0.8
D_B [Å]	3	39.5	36.8	43.4
D_{HH} [Å]	3	36.0	37.1	41.5
$2D_C$ [Å]	3	31.2	28.9	33.6
D_{H1} [Å]	20	2.4	4.1	4.0
A [Å^2]	2	60.5	65.3	56.2
z_{BB} [Å]	8	17.0	17.0	19.8
σ_{BB} [Å]	20	2.5	2.5	3.2
z_P [Å]	8	18.8	20.0	21.1
σ_P [Å]	20	3.5	3.0	2.9
z_h [Å]	3	23.8	20.0	21
$\sigma_h^\dagger$ [Å]		2.8	3	3.0
σ_{HC} [Å]		2.5	2.5	2.5
σ_T [Å]	5	3.0	3.0	4.1
σ_{poly} [%]	6	4.0	8.1	3.5
$V_{W,bound}$ [Å^3]	6	29.6	29.4	27.3
n_W	6	13.8	10.0	4.4
Y	9	0.40	0.45	0.62

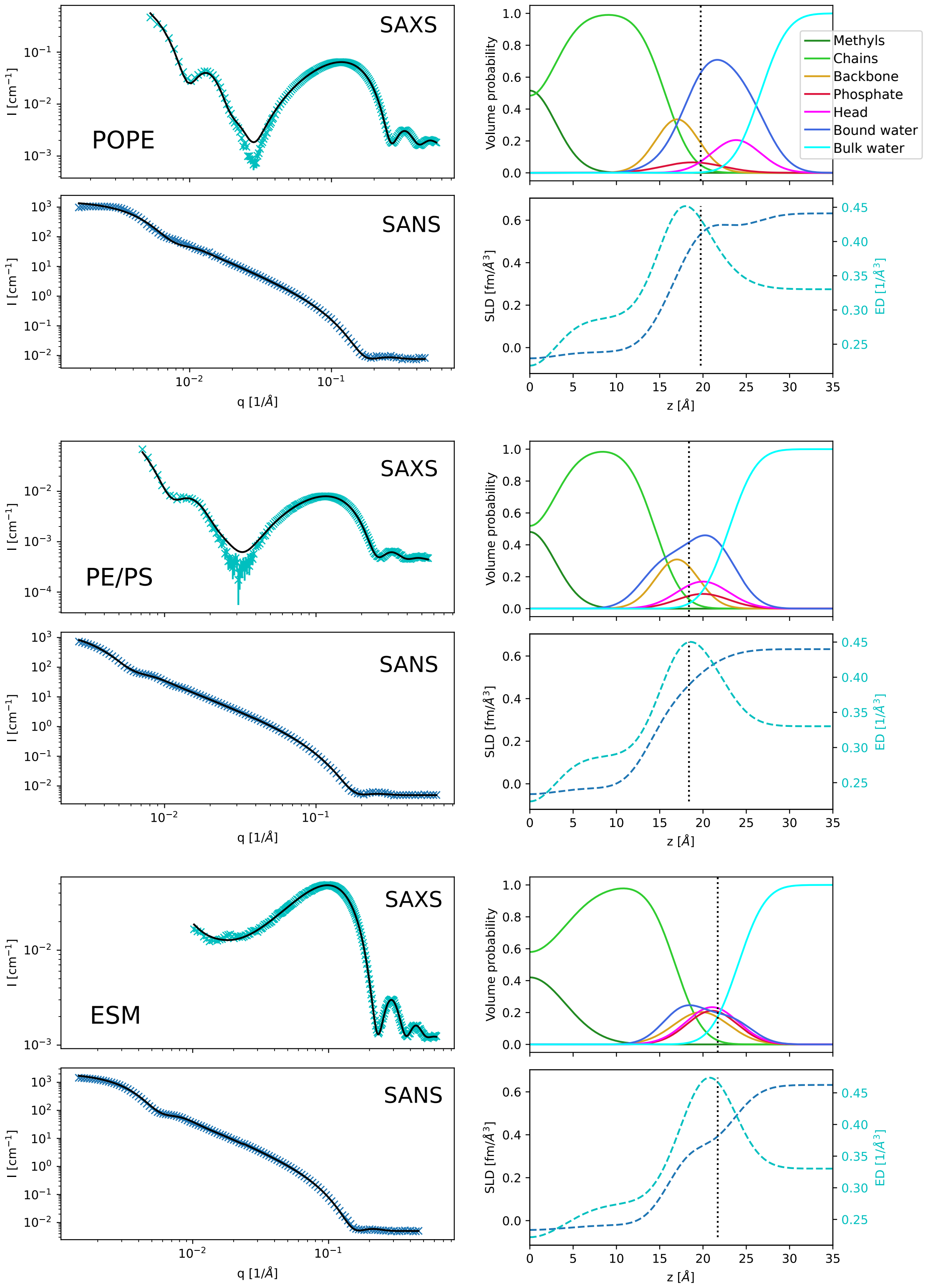

Figure S5: SAXS and SANS data with fits (black lines); SDP volume probability, electron density and neutron scattering length density profiles for symmetric lipid vesicles containing POPE/POPG 9:1, POPE/POPS 7:3 and Egg-SM/DPPG 19:1.

7 SAS-model parameters of asymmetric vesicles

Table S5: SAS-fitting parameters of aLUVs containing either a POPE/POPG 9:1 mixture (POPE) or a POPE/POPS 7:3 mixture (PE/PS) as acceptor lipids and ESM, POPC, MSM or a 1:1 POPC/MSM mixture (PC/SM) as donor lipids.

	ϵ [%]	POPE ESM	POPE POPC	POPE MSM	POPE PC/SM	PE/PS POPC	PE/PS MSM	PE/PS PC/SM
Total acc/don %	5	61:39	77:23	67:33	52:48	71:29	59:41	62:38
In acc/don %	5	98:2	90:10	82:18	65:35	97:3	97:3	77:23
Out acc/don %	5	28:72	65:35	54:46	40:60	48:52	25:75	49:51
D_B [Å]	3	37.6	39.0	40.3	39.6	37.1	40.0	39.1
D_{HH} [Å]	3	37.3	36.6	37.8	37.8	34.3	37.4	38.5
$2D_C$ [Å]	3	29.5	30.4	31.9	30.9	28.8	31.6	30.5
D_C^{in} [Å]	5	14.3	15.2	15.4	15.7	13.5	16.3	15.1
D_C^{out} [Å]	5	15.2	15.2	16.5	15.2	15.3	15.3	15.5
D_{H1}^{in} [Å]	20	4.6	2.8	3.3	3.8	2.3	3.4	3.9
D_{H1}^{out} [Å]	20	3.2	3.4	2.6	3.2	3.2	2.4	4.1
A^{av} [Å^2]	2	64.0	62.0	61.4	62.9	66.0	62.8	63.4
z_{BB}^{in} [Å]	6	-16.4	-16.0	-16.2	-16.5	-14.3	-17.1	-15.9
z_{BB}^{out} [Å]	6	17.4	16.0	17.3	16.0	16.1	16.1	16.3
$\sigma_{BB}^{in/out}$ [Å]		2.5	2.5	2.5	2.5	2.5	2.5	2.5
z_P^{in} [Å]	10	-19.4	-21.0	-19.2	-21.2	-17.3	-20.1	-19.3
z_P^{out} [Å]	10	22.4	19.0	21.5	19.0	19.7	19.9	20
σ_P^{in} [Å]	10	2.0	3.5	2	3.0	4.0	2.0	2
σ_P^{out} [Å]	10	4.0	2.0	3.5	2.3	3.0	3.8	2
z_h^{in} [Å]	10	-21.7	-24.0	-22.2	-24.2	-20.3	-23.1	-22.3
z_h^{out} [Å]	10	24.6	22.0	24.5	22.0	22.7	22.9	23
$\sigma_h^{in/out\dagger}$ [Å]		3.0	3.0	3	3.0	3.0	3.0	3
$\sigma_{HC}^{in/out}$ [Å]		2.5	2.5	2.5	2.5	2.5	2.5	2.5
σ_T [Å]	20	3.7	3.0	3.4	3.7	5.0	3.6	3.1
σ_{poly} [%]	6	0.0	3.9	4.3	5.0	10.0	7.0	7
$V_{W,bound}$ [Å^3]	6	30.3	30.3	30.2	30.0	29.4	29.6	29.3
n_W^{in}	6	14.4	15.4	11.8	14.6	14.2	10.3	12.8
n_W^{out}	6	16.7	11.0	13.4	11.5	11.6	14.8	12.7
R_m [Å]	10	361	361	345.3	373.4	369	361.2	381.9
σ_R [Å]	10	133	133	101.4	111.7	116.1	133	134.7

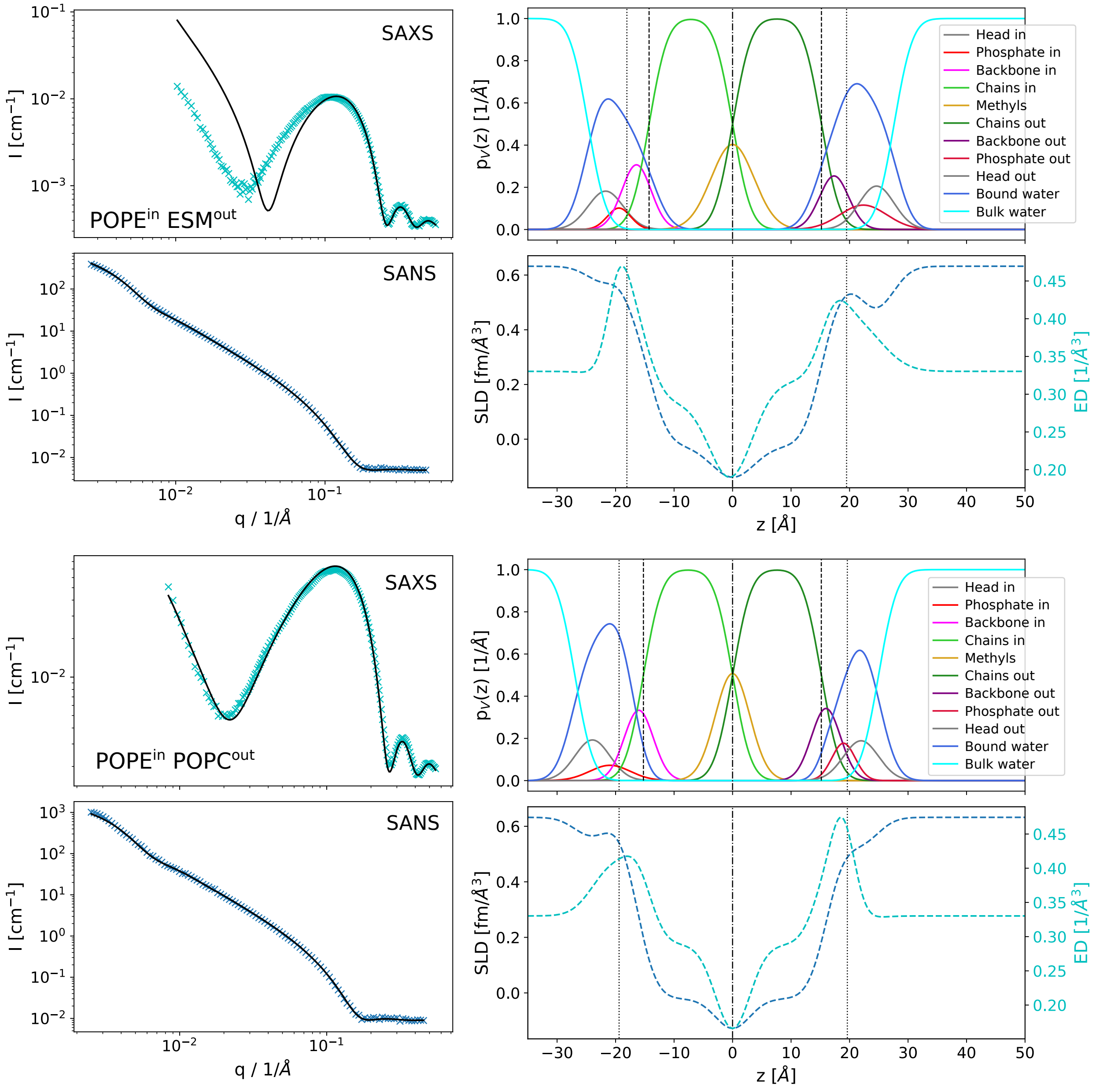

Figure S6: SAXS and SANS data with fits (black lines); SDP volume probability, electron density and neutron scattering length density profiles for the systems $POPE^{in}/ESM^{out}$ and $POPE^{in}/POPC^{out}$.

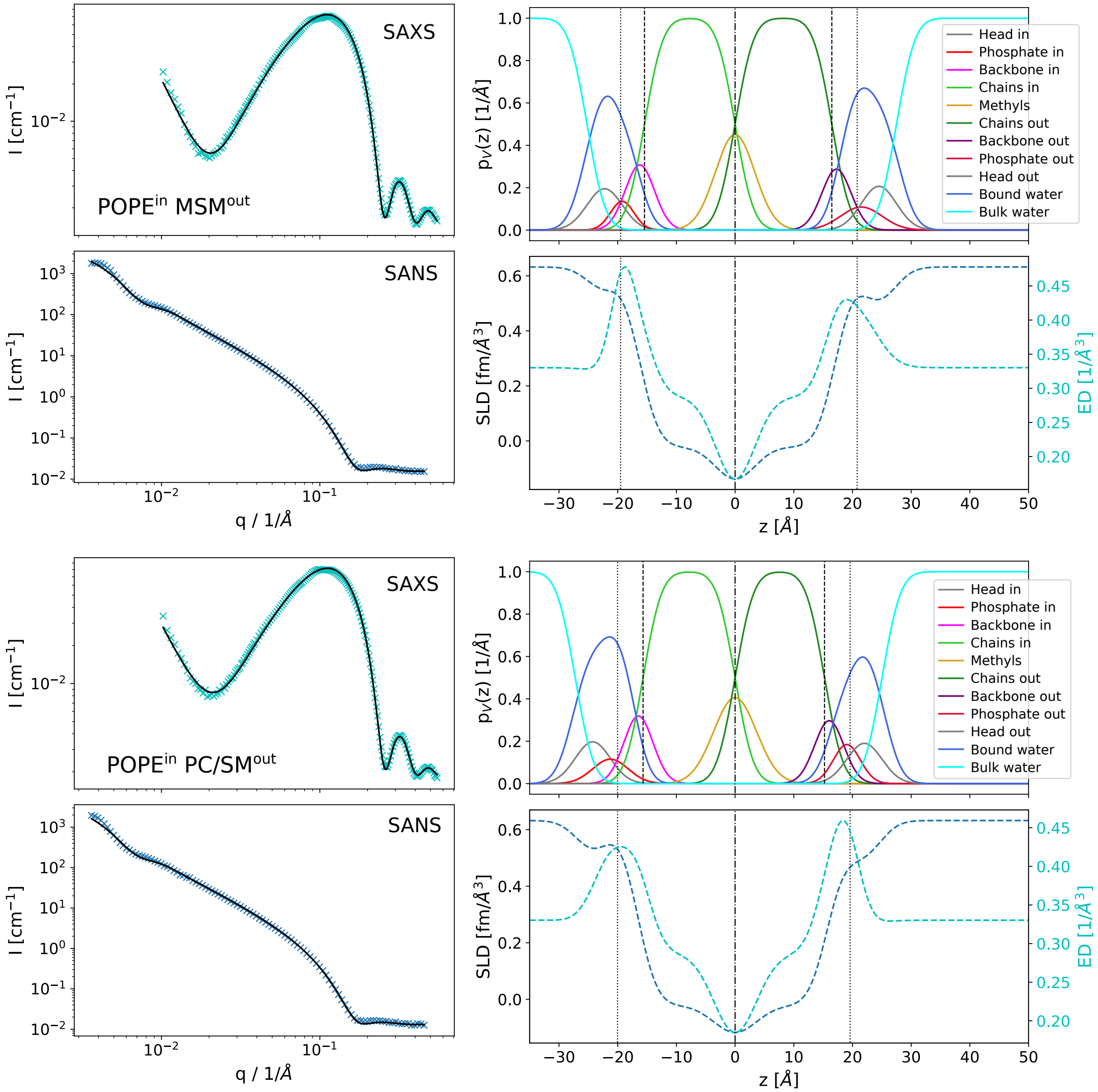

Figure S7: SAXS and SANS data with fits (black lines); SDP volume probability, electron density and neutron scattering length density profiles for the systems POPE^{in}/MSM^{out} and POPE^{in}/(PC/SM)^{out}.

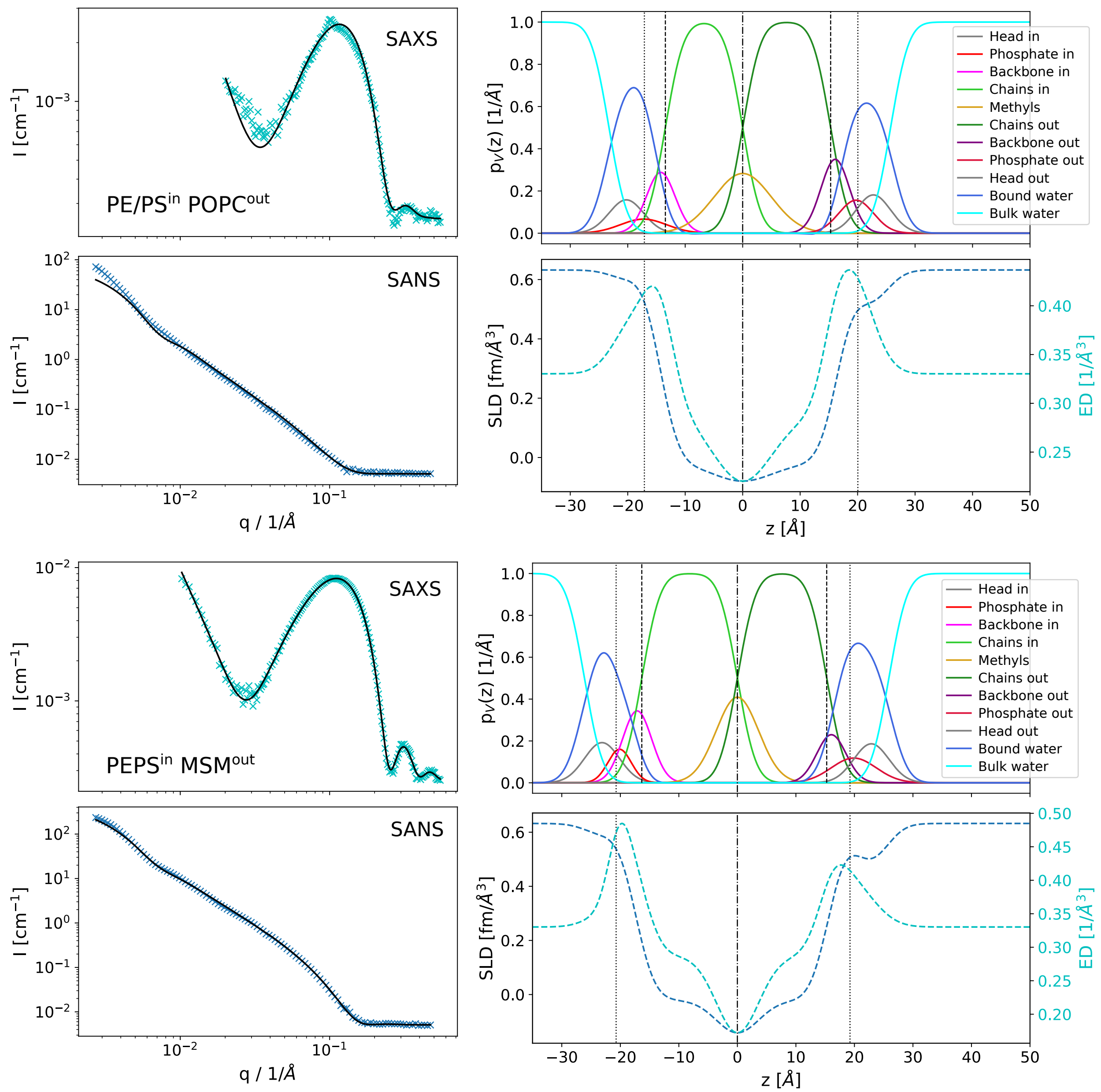

Figure S8: SAXS and SANS data with fits (black lines); SDP volume probability, electron density and neutron scattering length density profiles for the systems (PE/PS)^{in}/POPC^{out} and (PE/PS)^{in}/MSM^{out}. SAXS data in the upper panel show a small bragg peak, which is probably connected to a small contamination of multilamellar donor vesicles in the sample. This could be also responsable for the disagreement between data and model in low-q SANS. We expect a higher experimental error for this sample.

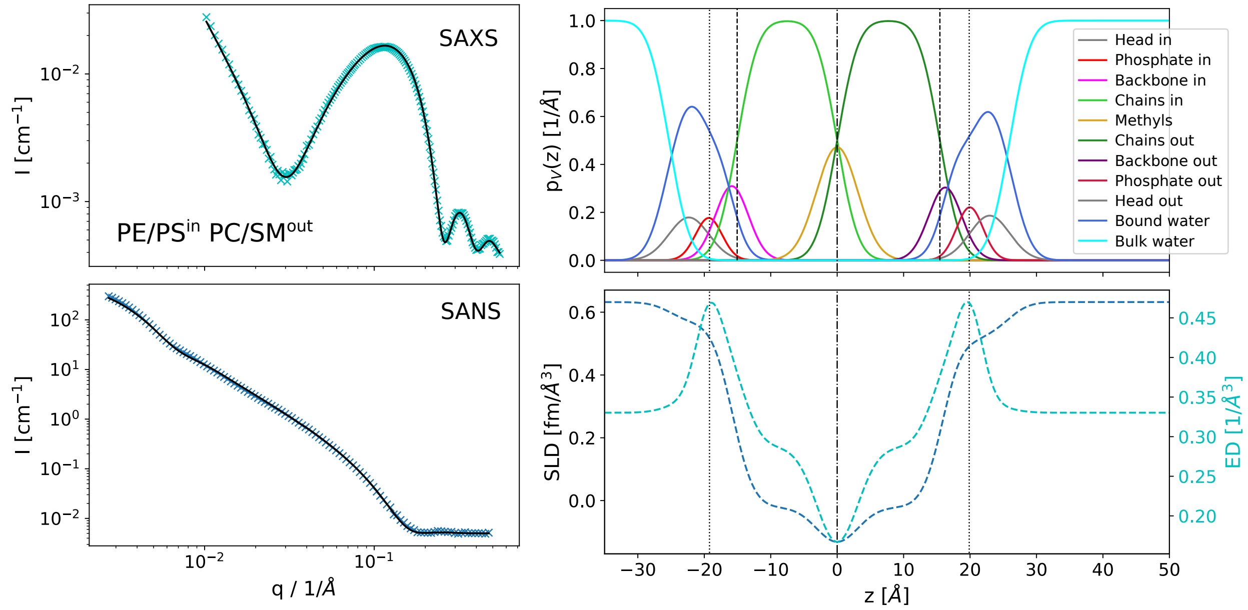

Figure S9: SAXS and SANS data with fits (black lines); SDP volume probability, electron density and neutron scattering length density profiles for the system $(PE/PS)^{in}/(PC/SM)^{out}$.

8 SAS-model parameters of reference LUVs

Table S6: SAS-fitting parameters of symmetric reference LUVs for POPE/POPG acceptor vesicles.
Inner leaflet samples (in): 90% POPE/POPG (9:1 mol/mol) and 10% POPC, MSM, or POPC/MSM (1:1 mol/mol).
Outer leaflet samples (out): 30% POPE/POPG (9:1 mol/mol) and 70% POPC, MSM, or POPC/MSM (1:1 mol/mol).
Scrambled vesicles (scram): same composition as aLUVs, see Tab. S5.

		POPC			MSM			PC/SM		
	ϵ [%]	in	out	scram	in	out	scram	in	out	scram
V_L^* [Å^3]		1202.1	1252.0	1212.94	1208.1	1293.56	1240.85	1205.11	1272.77	1247.96
V_H^* [Å^3]		257.4	304.48	267.63	252.0	266.68	257.65	254.74	285.58	274.27
r_{BB}^*		0.50	0.46	0.49	0.49	0.38	0.45	0.5	0.42	0.45
r_P^*		0.16	0.25	0.18	0.16	0.27	0.2	0.16	0.26	0.22
r^*		2.09	2.09	2.09	2.09	2	2.09	2.09	2.09	2.09
r_{12}^*		0.8	0.8	0.8	0.8	1	0.8	0.8	0.8	0.8
D_B [Å]	3	39.6	38.9	37.37	40.6	43.13	38.87	39.92	41.28	38.36
D_{HH} [Å]	3	37.2	35.6	33.84	37.0	39.44	35.59	36.3	37.53	35.31
$2D_C$ [Å]	3	31.1	29.4	29.12	32.2	34.23	30.79	31.47	32.01	29.92
D_{H1} [Å]	20	3.1	3.1	2.36	2.4	2.61	2.4	2.41	2.76	2.7
A [Å^2]	2	60.8	64.5	64.93	59.5	60	63.86	60.39	61.68	65.08
z_{BB} [Å]	8	17.9	17.1	15.65	17.7	18.24	16.53	17.13	18.04	16.63
σ_{BB} [Å]	20	2.5	2.5	2.5	2.5	2.5	2.5	2.5	2.5	2.5
z_P [Å]	8	19.1	18.2	17.66	18.9	20.83	18.81	19.15	19.21	18.21
σ_P [Å]	20	3.0	3.2	3.2	3.0	3	3.3	3.5	3.01	3.05
z_h [Å]	3	22.0	21.5	19.95	23	23.72	21.37	23.06	21.1	20.02
$\sigma_h^\dagger$ [Å]		2.8	2.8	2.8	2.8	2.8	2.8	2.8	2.8	2.8
σ_{HC} [Å]		2.5	2.5	2.5	2.5	2.5	2.5	2.5	2.5	2.5
σ_T [Å]	5	2.4	2.4	2.79	2.4	4	3.76	3.51	2.7	4
σ_{poly} [%]	6	4.8	3.22	5.96	5.4	6.34	4.82	4.31	3.66	7.5
$V_{W,bound}$ [Å^3]	6	29.8	29.9	28.83	29.5	29.8	29.01	29.75	29.87	28.47
n_W	6	10.0	10.3	8.73	10.8	9.83	10	11.77	6.66	7.84
R_m [Å]	10	370.1	370.2	430.7	372.5	470.2	408.6	356.2	391.4	390.6
σ_R [Å]	10	103.9	123.7	171.7	102.3	127.7	182.9	91.67	106.7	188.3

Table S7: SAS-fitting parameters of symmetric reference LUVs for POPE/POPS acceptor vesicles.
Inner leaflet samples (in): 90% POPE/POPS (7:3 mol/mol) and 10% POPC, MSM, or POPC/MSM (1:1 mol/mol).
Outer leaflet samples (out): 30% POPE/POPS (7:3 mol/mol) and 70% POPC, MSM, or POPC/MSM (1:1 mol/mol).
Scrambled vesicles (scram): same composition as aLUVs, see Tab. S5.

		POPC			MSM			PC/SM		
	ϵ [%]	in	out	scram	in	out	scram	in	out	scram
V_L^* [Å^3]		1207.1	1253.6	1221.8	1213.03	1295.21	1255.49	1210.06	1274.42	1240.09
V_H^* [Å^3]		262.2	306.07	276.1	256.81	268.27	262.73	259.51	287.17	272.42
r_{BB}^*		0.49	0.46	0.48	0.48	0.37	0.42	0.48	0.41	0.45
r_P^*		0.19	0.26	0.21	0.19	0.28	0.24	0.19	0.27	0.23
r^*		2.09	2.09	2.09	2.09	2	2.09	2.09	2.09	2.09
r_{12}^*		0.8	0.8	0.8	0.8	1	0.8	0.8	0.8	0.8
D_B [Å]	3	37.8	38.2	37.2	38.22	42.65	39.08	38.06	40.22	37.09
D_{HH} [Å]	3	37.0	35.3	32.7	36.85	38.99	38.93	36.28	37.2	36.53
$2D_C$ [Å]	3	29.6	28.8	28.8	30.12	33.81	30.89	29.89	31.15	28.93
D_{H1} [Å]	20	3.7	3.2	2.0	3.37	2.59	4.02	3.19	3.03	3.8
A [Å^2]	2	63.8	65.7	65.8	63.49	60.75	64.27	63.61	63.39	66.89
z_{BB} [Å]	8	16.3	16.1	16.0	17.22	18.53	19.39	16.8	17.84	17.31
σ_{BB} [Å]	20	2.5	2.5	2.5	2.5	2.5	2.86	2.5	2.5	2.5
z_P [Å]	8	19.1	18.3	16.0	19.92	20.34	19.39	19.59	19.1	19.42
σ_P [Å]	20	2.2	2.8	2.9	3.5	3.29	2.2	3.5	3.27	3.5
z_h [Å]	3	24.1	23.3	21	19.92	21.77	24.39	19.59	19.47	19.42
$\sigma_h^\dagger$ [Å]		2.8	2.8	2.8	2.8	2.8	2.8	2.8	2.8	2.8
σ_{HC} [Å]		2.5	2.5	2.5	2.5	2.5	2.5	2.5	2.5	2.5
σ_T [Å]	5	3.5	3.1	4.0	3.32	3.89	2.4	3.26	3.19	4
σ_{poly} [%]	6	7.3	6.63	10.0	5.26	4.81	9.46	6.08	5.58	6.92
$V_{W,bound}$ [Å^3]	6	30.0	29.7	30.3	30.03	29.78	30.01	28.97	29.26	29.41
n_W	6	16.9	15.2	11.4	7.58	6.53	16.25	7.07	4.54	8.14
R_m [Å]	10	381	392.6	469.1	409.5	495.7	607.7	430.9	451.9	500.7
σ_R [Å]	10	109.2	133.5	154.4	131.7	167.1	151.9	140.6	133.1	128.7

Table S8: SAS-fitting parameters of symmetric reference LUVs for POPE/POPG acceptor vesicles.
Inner leaflet samples (in): 90% POPE/POPG (9:1 mol/mol) and 10% ESM.
Outer leaflet samples (out): 30% POPE/POPG (9:1 mol/mol) and 70% ESM.
Scrambled vesicles (scram): same composition as aLUVs, see Tab. S5.

	ϵ [%]	ESM in	out	scram
V_L^* [Å^3]		1196.5	1212.2	1181.0
V_H^* [Å^3]		252.0	266.68	259.1
r_{BB}^*		0.49	0.38	0.44
r_P^*		0.16	0.27	0.21
r^*		2.09	2.09	2.09
r_{12}^*		0.8	0.8	0.8
D_B [Å]	3	38.0	41.1	38.2
D_{HH} [Å]	3	36.9	37.0	34.9
$2D_C$ [Å]	3	30.0	32.0	29.8
D_{H1} [Å]	20	3.4	2.5	2.6
A [Å^2]	2	63.0	59.1	61.8
z_{BB} [Å]	8	17.6	17.6	15.7
σ_{BB} [Å]	20	2.5	2.5	2.5
z_P [Å]	8	19.4	18.7	20.2
σ_P [Å]	20	3.5	2.7	3.5
z_h [Å]	3	19.4	22.9	21
$\sigma_h^\dagger$ [Å]		2.8	2.8	2.8
σ_{HC} [Å]		2.5	2.5	2.5
σ_T [Å]	5	3.1	2.4	4.0
σ_{poly} [%]	6	5.3	4.95	8.5
$V_{W,bound}$ [Å^3]	6	27.7	27.6	30.3
n_W	6	6.7	10.0	8.8
R_m [Å]	10	393.1	455	531.6
σ_R [Å]	10	114.1	132.9	138.9

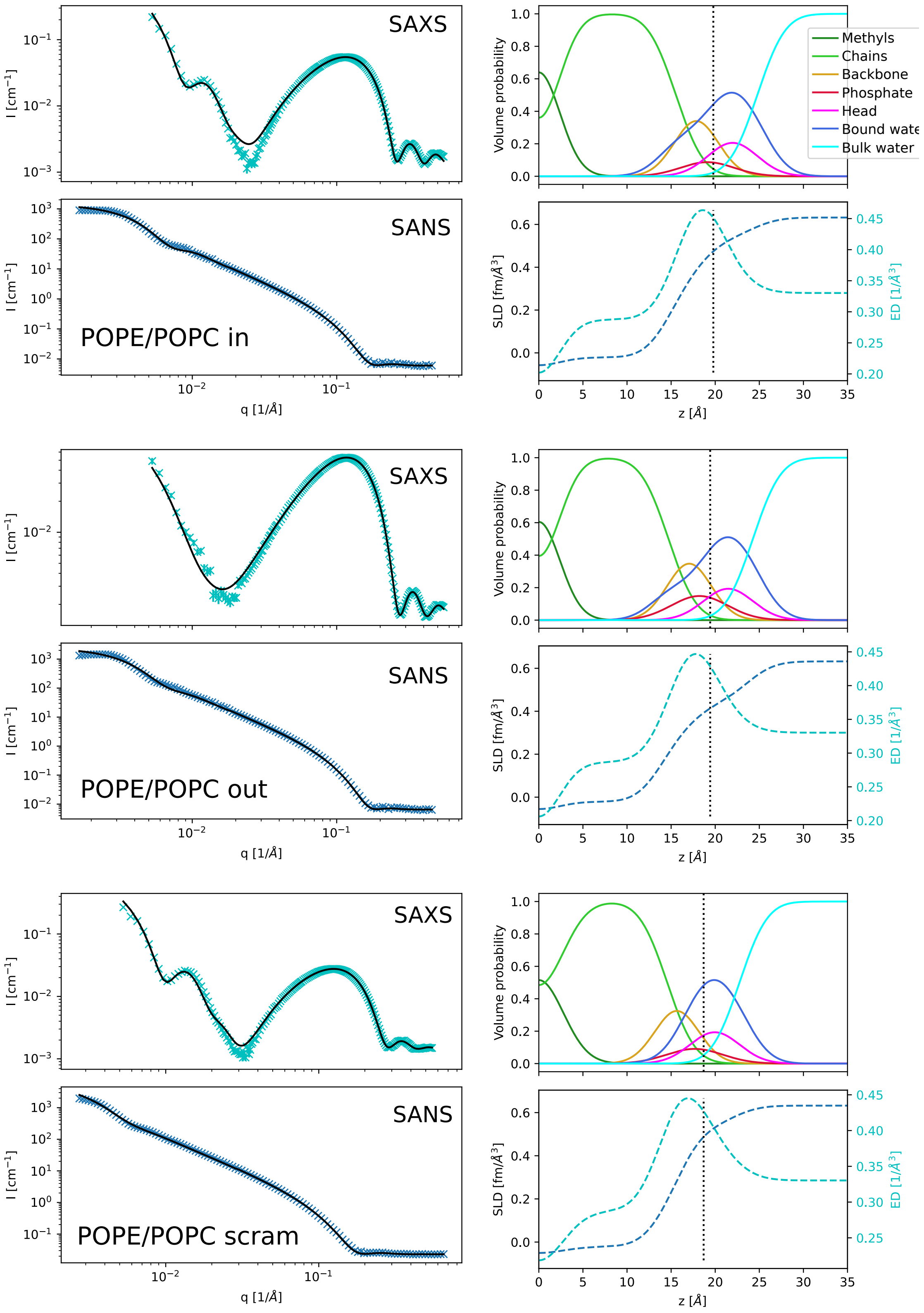

Figure S10: SAXS and SANS data with fits (black lines); SDP volume probability, electron density and neutron scattering length density profiles for POPE/POPC inner/outer leaflet symmetric mimics, as well as the scrambled sample.

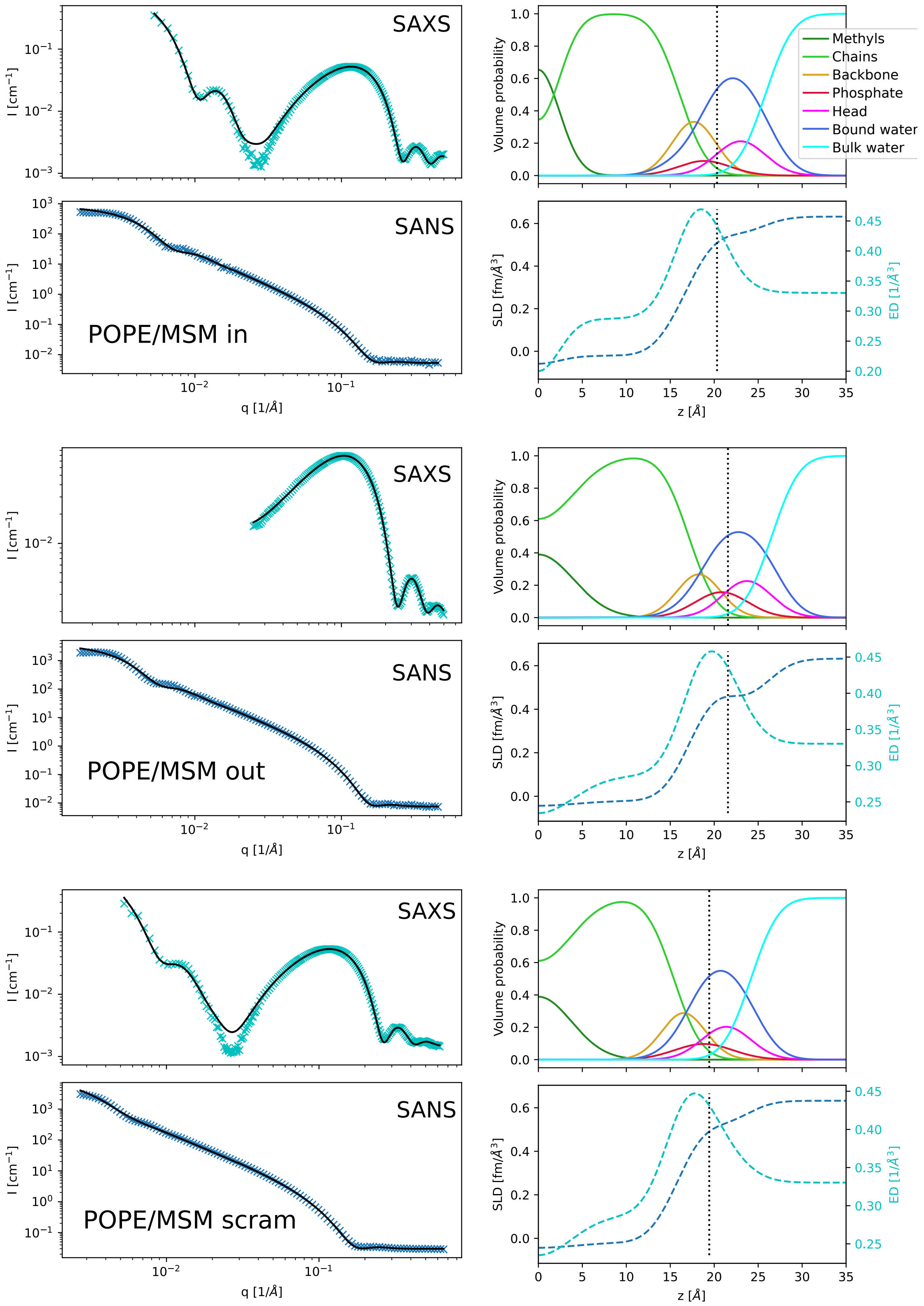

Figure S11: SAXS and SANS data with fits (black lines); SDP volume probability, electron density and neutron scattering length density profiles for POPE/MSM inner/outer leaflet symmetric mimics, as well as the scrambled sample.

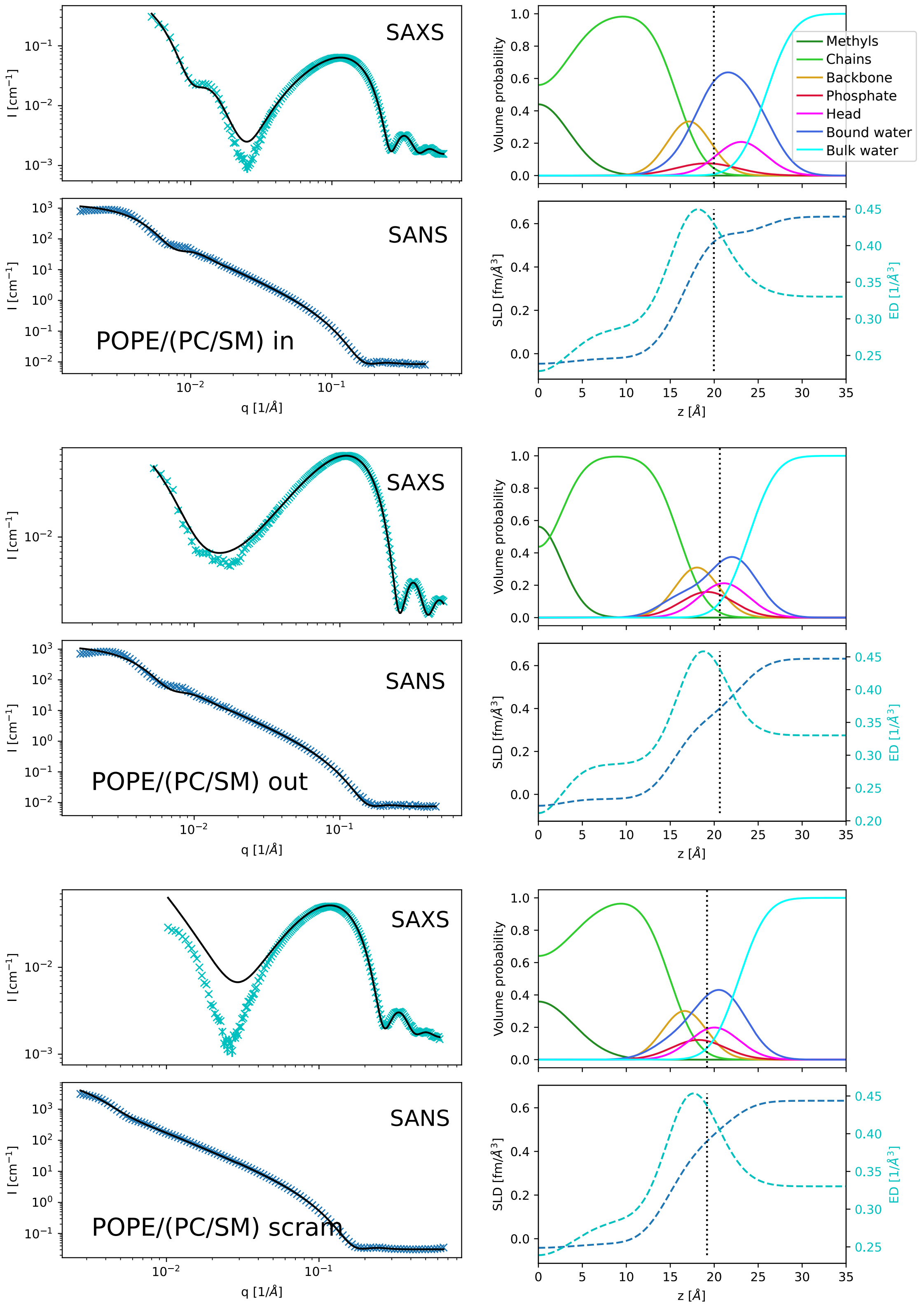

Figure S12: SAXS and SANS data with fits (black lines); SDP volume probability, electron density and neutron scattering length density profiles for POPE/(PC/SM) inner/outer leaflet symmetric mimics, as well as the scrambled sample.

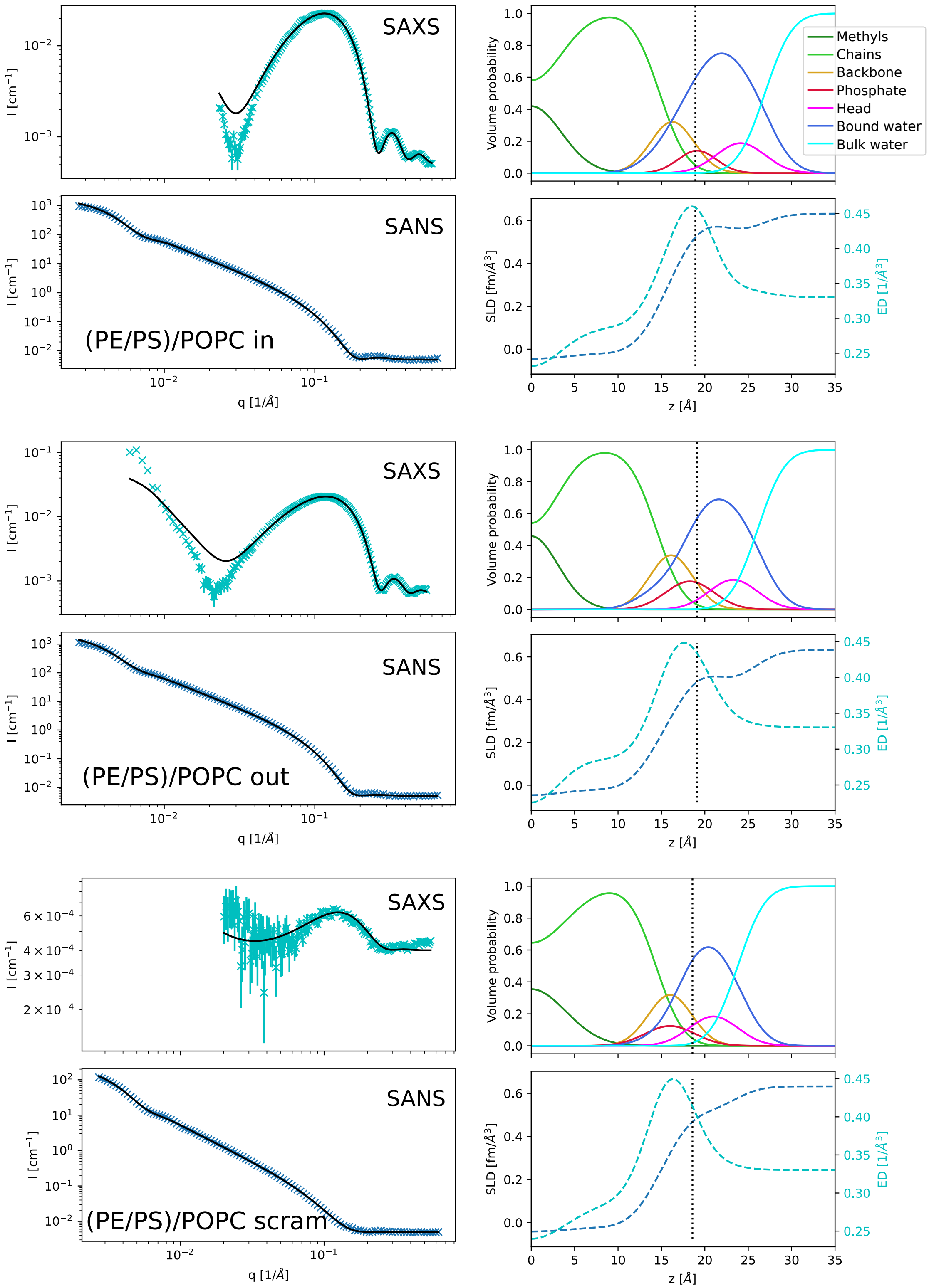

Figure S13: SAXS and SANS data with fits (black lines); SDP volume probability, electron density and neutron scattering length density profiles for (PE/PS)/POPC inner/outer leaflet symmetric mimics, as well as the scrambled sample.

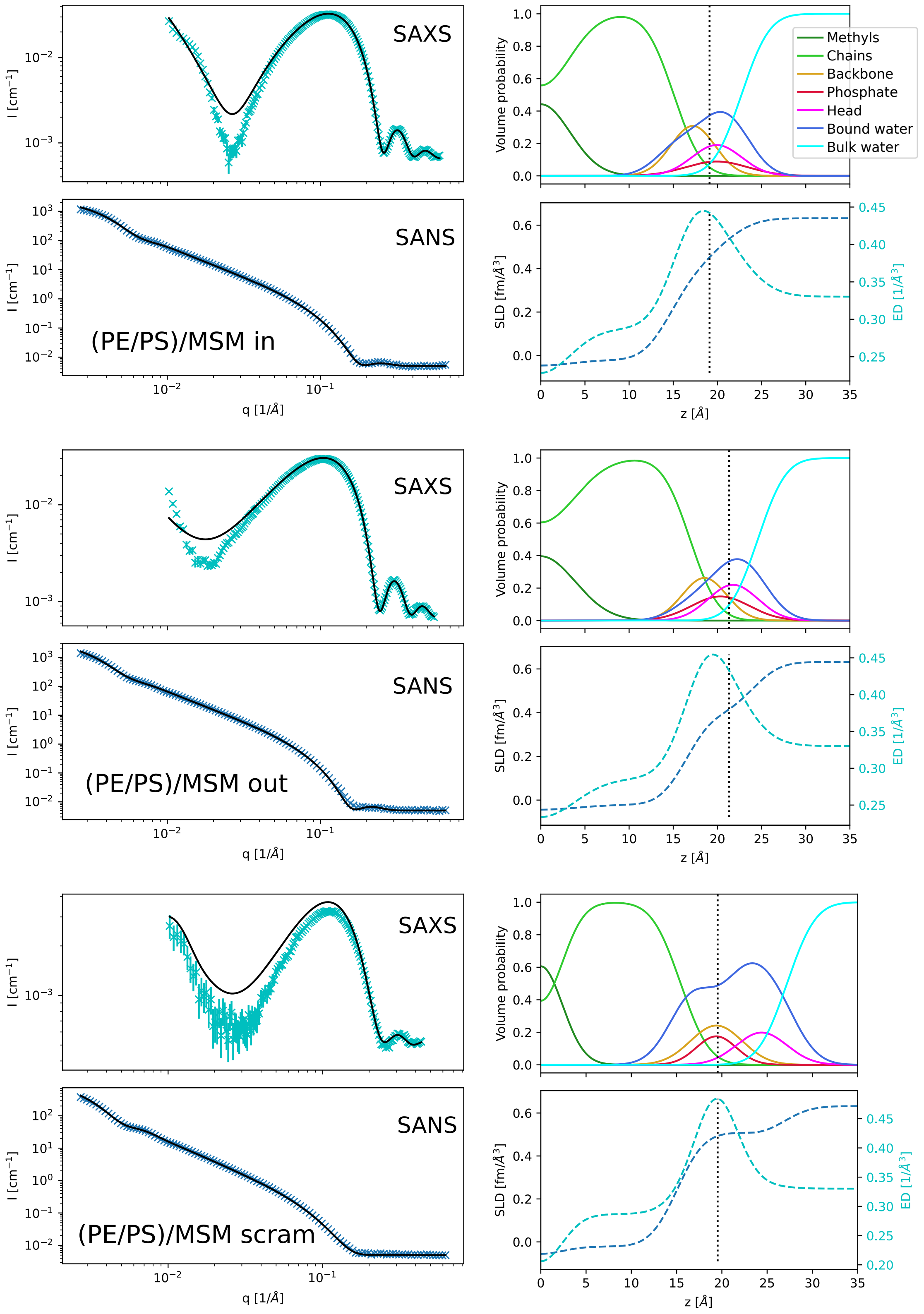

Figure S14: SAXS and SANS data with fits (black lines); SDP volume probability, electron density and neutron scattering length density profiles for (PE/PS)/MSM inner/outer leaflet symmetric mimics, as well as the scrambled sample.

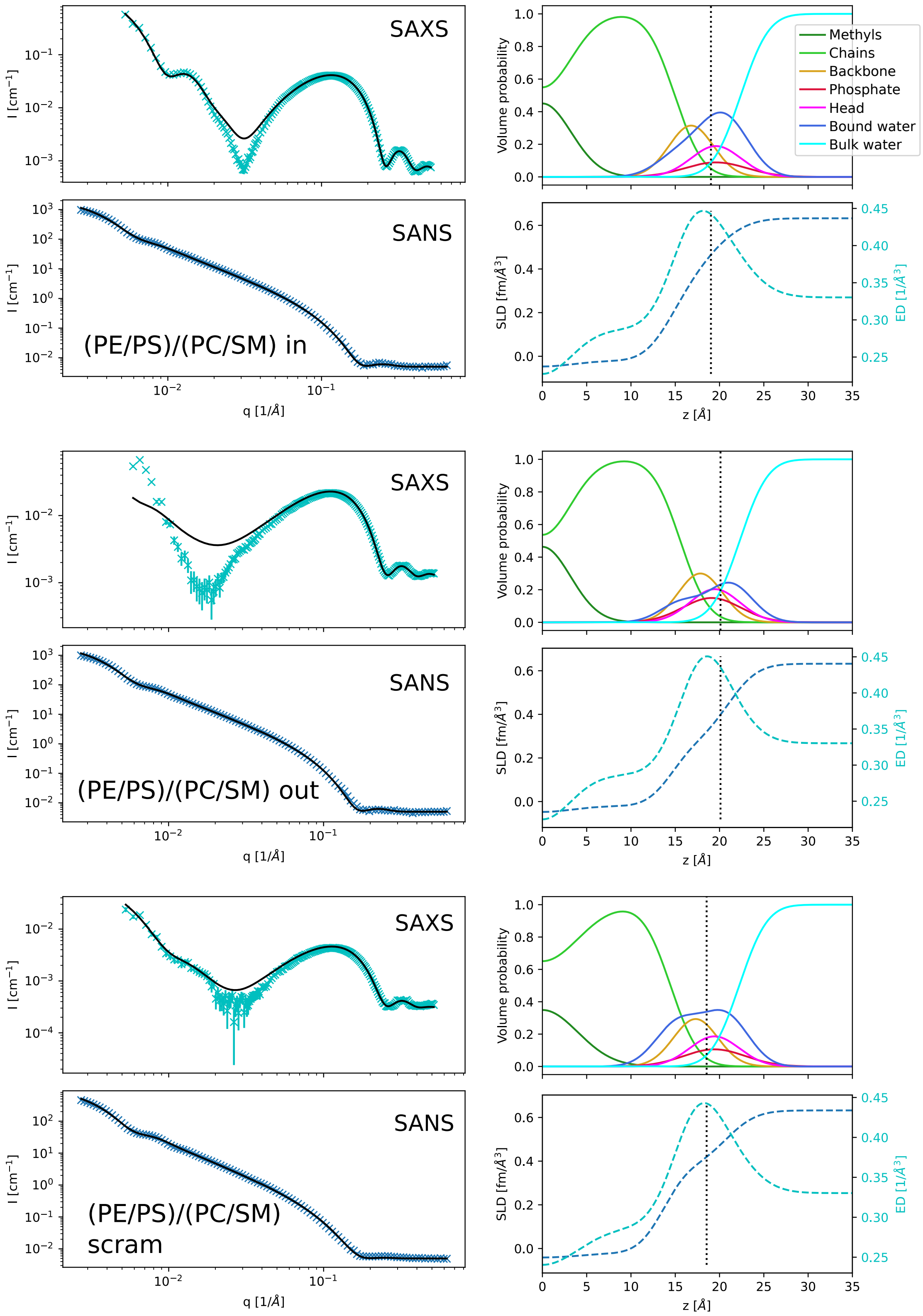

Figure S15: SAXS and SANS data with fits (black lines); SDP volume probability, electron density and neutron scattering length density profiles for (PE/PS)/(PC/SM) inner/outer leaflet symmetric mimics, as well as the scrambled sample.

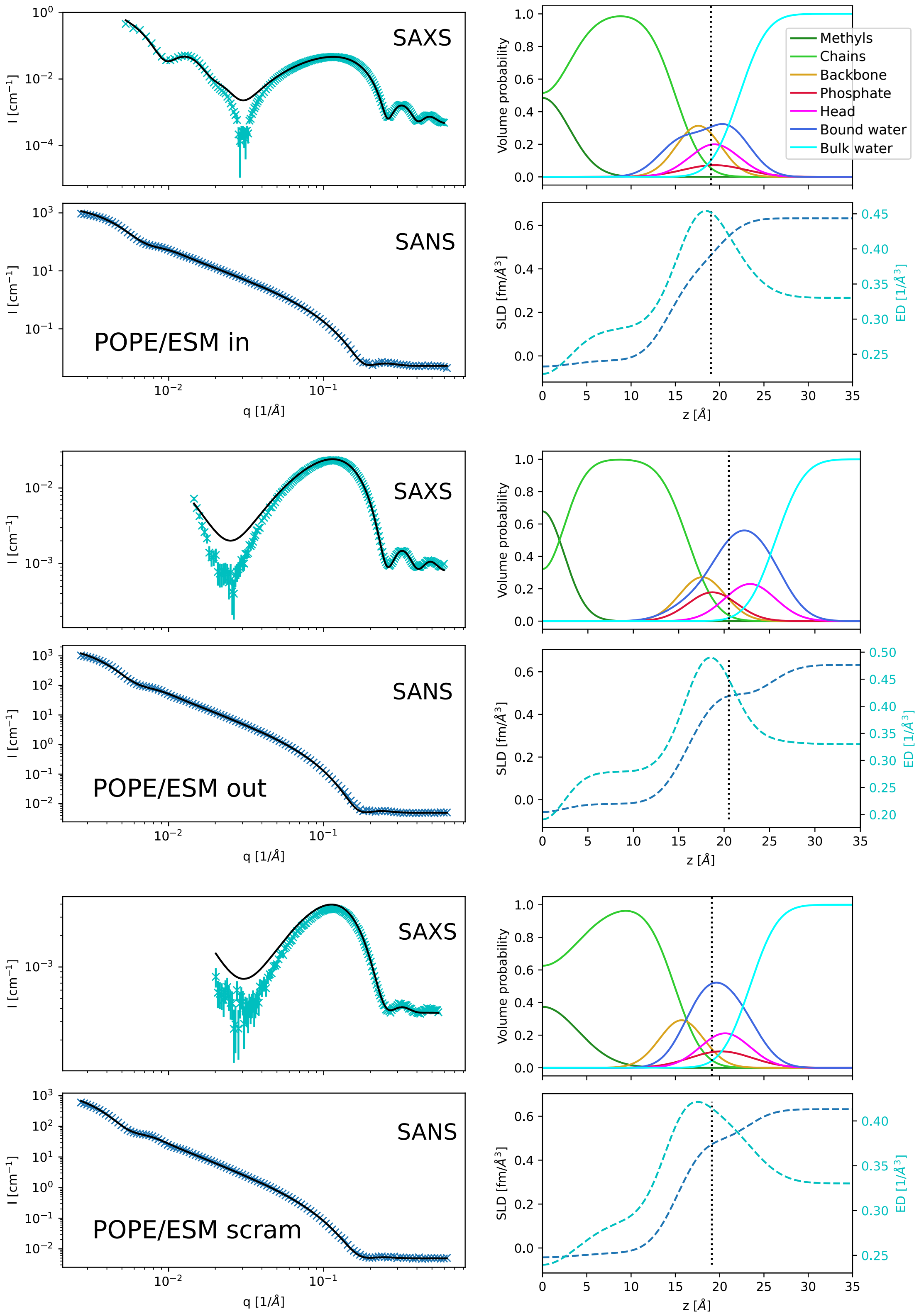

Figure S16: SAXS and SANS data with fits (black lines); SDP volume probability, electron density and neutron scattering length density profiles for POPE/ESM inner/outer leaflet symmetric mimics, as well as the scrambled sample.

References

[1] Ali Imran, Dumitru Popescu, and Liviu Movileanu. Cyclic activity of an osmotically stressed liposome in a finite hypotonic environment. Langmuir, 36(13):3659–3666, 2020.

[2] A Srinivas Reddy, Dora Toledo Warshaviak, and Mirianas Chachisvilis. Effect of membrane tension on the physical properties of dopc lipid bilayer membrane. Biochimica et Biophysica Acta (BBA)-Biomembranes, 1818(9):2271–2281, 2012.

[3] Rubèn Serral Gracià, Natalya Bezlyepkina, Roland L. Knorr, Reinhard Lipowsky, and Rumiana Dimova. Effect of cholesterol on the rigidity of saturated and unsaturated membranes: fluctuation and electrodeformation analysis of giant vesicles. Soft Matter, 6(7):1472–1482, 2010.

[4] W. Rawicz, K.C. Olbrich, T. McIntosh, D. Needham, and E. Evans. Effect of chain length and unsaturation on elasticity of lipid bilayers. Biophysical Journal, 79:328–339, 7 2000.

[5] Moritz P. K. Frewein, Paulina Piller, Enrico F. Semeraro, Krishna C. Batchu, Frederick A. Heberle, Haden L. Scott, Yuri Gerelli, Lionel Porcar, and Georg Pabst. Interdigitation-induced order and disorder in asymmetric membranes. The Journal of Membrane Biology, 4 2022.

[6] Moritz P. K. Frewein, Milka Doktorova, Frederick A. Heberle, Haden L. Scott, Enrico F. Semeraro, Lionel Porcar, and Georg Pabst. Structure and interdigitation of chain-asymmetric phosphatidylcholines and milk sphingomyelin in the fluid phase. Symmetry, 13:1441, 8 2021.

[7] Norbert Kučerka, Jeremy Pencer, Jonathan N. Sachs, John F. Nagle, and John Katsaras. Curvature effect on the structure of phospholipid bilayers. Langmuir, 23(3):1292–1299, 2007.
