# Supplementary material for: Distributing aminophospholipids asymmetrically across leaflets causes anomalous membrane stiffening
Source: Biophys J. 2023 Apr 29;122(12):2445–55. doi: 10.1016/j.bpj.2023.04.025 (PMC10322881; doi:10.1016/j.bpj.2023.04.025)
Supplement: Document S2. Article plus supporting material [file mmc2.pdf]

Biophysical Journal
Article

Biophysical Society

Distributing aminophospholipids asymmetrically across leaflets causes anomalous membrane stiffening

Moritz P. K. Frewein,[1,2,3,4] Paulina Piller,[1,3,4] Enrico F. Semeraro,[1,3,4] Orsolya Czakkel,[2] Yuri Gerelli,[5,6] Lionel Porcar,[2] and Georg Pabst[1,3,4,*]

[1]Biophysics, Institute of Molecular Biosciences, University of Graz, NAWI Graz, Graz, Austria; [2]Institut Laue-Langevin, Grenoble, France; [3]BioTechMed Graz, Graz, Austria; [4]Field of Excellence BioHealth, Graz, Austria; [5]CNR Institute for Complex Systems, Uos Sapienza, Roma, Italy; and [6]Department of Physics, Sapienza University of Rome, Roma, Italy

ABSTRACT We studied the mechanical leaflet coupling of prototypic mammalian plasma membranes using neutron spin-echo spectroscopy. In particular, we examined a series of asymmetric phospholipid vesicles with phosphatidylcholine and sphingomyelin enriched in the outer leaflet and inner leaflets composed of phosphatidylethanolamine/phosphatidylserine mixtures. The bending rigidities of most asymmetric membranes were anomalously high, exceeding even those of symmetric membranes formed from their cognate leaflets. Only asymmetric vesicles with outer leaflets enriched in sphingolipid displayed bending rigidities in conformity with these symmetric controls. We performed complementary small-angle neutron and x-ray experiments on the same vesicles to examine possible links to structural coupling mechanisms, which would show up in corresponding changes in membrane thickness. In addition, we estimated differential stress between leaflets originating either from a mismatch of their lateral areas or spontaneous curvatures. However, no correlation with asymmetry-induced membrane stiffening was observed. To reconcile our findings, we speculate that an asymmetric distribution of charged or H-bond forming lipids may induce an intraleaflet coupling, which increases the weight of hard undulatory modes of membrane fluctuations and hence the overall membrane stiffness.

SIGNIFICANCE Biological membranes serve as more than just outer barriers for cells and organelles; they also contain a complex machinery of proteins that is not only sensitive to the surrounding lipid composition, but also to the mechanical properties of its hosting bilayer. Little is known about the effect of transbilayer lipid asymmetry, a hallmark of all plasma membranes, on bilayer structure and dynamics. The results of this study indicate that the composition of the plasma membrane leads to unique mechanical coupling mechanisms, suggesting that the regulation of lipid asymmetry through flipases/flopases and scramblases could be an important means to tune the mechanical stiffness of the membrane.

INTRODUCTION

Cellular envelopes contain a large number of lipid species that are distributed asymmetrically between the two leaflets of the bilayer. For example, mammalian plasma membranes are known to be composed of an outer leaflet enriched in choline phospholipids, while the majority of the aminophospholipids are confined to the inner leaflet (1,2). Membrane asymmetry is generated and maintained by an array of enzymes termed flipases, flopases, and scramblases (3). However, the physiological need for or benefits from membrane asymmetry are far from being understood. For example, the different chemical potentials stored in both leaflets might induce a coupling that can be exploited, e.g., in signaling or transport processes such as endo/exocytosis even in the absence of proteins (4,5). Currently conceived lipid-mediated coupling mechanisms consider intrinsic lipid curvature, headgroup electrostatics, cholesterol flip-flop, dynamic chain interdigitation, or differential stress between lipid leaflets (6–8).

Transleaflet coupling has been reported to induce domains in nondomain-forming lipid mixtures (9,10), affect lateral lipid diffusion (11), or cross-link the melting transitions of individual leaflets (7). Recently, we reported that

Submitted December 22, 2022, and accepted for publication April 25, 2023.

*Correspondence: georg.pabst@uni-graz.at

Editor: Andreas Janshoff.

https://doi.org/10.1016/j.bpj.2023.04.025

© 2023 Biophysical Society.

This is an open access article under the CC BY license (http://creativecommons.org/licenses/by/4.0/).

the extent of hydrocarbon interdigitation allows membranes to tweak the lateral packing of lipids in the opposing leaflet through competing cohesive and entropic interactions of the acyl chains (12). In addition, membrane asymmetry can lead to distinct effects on its elastic behavior, either due to lipid-specific properties (e.g., size, shape) or simply lipid over/undercrowding of a given leaflet (8,13). Reported experimental data for membrane asymmetry-induced stiffening agree at least qualitatively with this theory but are scarce (14–17). In particular, none of the previous reports interrogated the effect of asymmetrically distributed charged lipids on the bilayer's bending rigidity. Note that charged lipids are able to increase the stiffness of symmetric bilayers significantly (18,19).

This prompted us to perform bending rigidity measurements on asymmetric membranes with—compared with previous reports—higher compositional complexity. In particular, we engineered mimics of mammalian plasma membranes with lipid architectures close to the reported distributions of phosphatidylcholine, sphingomyelin, phosphatidylethanolamine, and phosphatidylserine in mammalian plasma membranes (2). Asymmetric large unilamellar vesicles (aLUVs) were produced using cylcodextrin-mediated lipid exchange (20) and studied by small-angle neutron scattering (SANS), small-angle x-ray scattering (SAXS), and neutron spin-echo spectroscopy (NSE). Cholesterol was deliberately excluded from the present study, despite its abundance in mammalian plasma membranes. Cholesterol is known to induce lipid domains in lipid mixtures mimicking mammalian membranes (9,10), which challenges nanostructural interpretation as attempted in this report. Leaving out cholesterol thus allowed us to probe pure phospholipid interactions without any interference from heterogeneities (domains) induced by cholesterol-lipid interactions.

We found that aLUVs with inner leaflets enriched either in palmitoyl oleoyl phosphatidylethanolamine (POPE), or POPE/phosphatidylserine (POPS) and outer leaflets containing palmitoyl oleoyl phosphatidylcholine (POPC), milk sphingomyelin (MSM), or equimolar mixtures of both lipids are always more rigid than symmetric vesicles of the same lipid composition. Moreover, most aLUVs were even more rigid than symmetric vesicles of their cognate leaflets, i.e., displayed anomalous stiffening. No anomalous stiffening was observed when only MSM was enriched in the outer leaflet, which can, due to its highly asymmetric hydrocarbon chain composition, interdigitate into the inner leaflet (12). Nanostructural data of the same samples allowed us to seek agreement with existing theories that couple membrane thickness (21) and differential stress (8) to the elastic behavior of membranes. However, we could not find a satisfactory agreement. We speculate that charge and H-bonding mediated intraleaflet interactions between aminophospholipids lead to a dominance of short wavelength fluctuations (hard modes) when these lipids are distributed asymmetrically in bilayers and hence to much increased bending rigidities. The absence of anomalous stiffening for MSM-containing asymmetric membranes suggests that hydrocarbon chain interdigitation can alleviate this effect.

MATERIALS AND METHODS

Lipids, chemicals, and sample preparation

POPC, POPE, POPS, and MSM, as well as egg sphingomyelin (ESM), dipalmitoylphosphatidylcholine (DPPC), dipalmitoylphosphatidylglycerol, dioleoyl phosphatidylcholine (DOPC), and palmitoyl oleoyl phosphatidylglycerol (POPG) were purchased from Avanti Polar Lipids (Alabaster, AL) and used without further purification. Chloroform, methanol (pro analysis grade), sucrose, and methyl-β-cyclodextrin (mβCD) were obtained from Merck KGaA (Darmstadt, Germany). We prepared asymmetric unilamellar vesicles following the heavy donor cyclodextrin exchange protocol (20). Acceptor and donor lipids were weighed (ratio 1:2 mol/mol), dissolved separately in a chloroform/methanol mixture (2:1, vol/vol) and dried under a soft argon stream in a glass vial. Acceptor vesicles were prepared from either DPPC with 5 mol % dipalmitoylphosphatidylglycerol, POPE with 10 mol % palmitoyl oleoyl phosphatidylglycerol, or POPE with 30 mol % POPS. Donor vesicles consisted of ESM, MSM, POPC, or equimolar POPC:MSM mixtures and did not contain any charged lipid.

The resulting dry lipid films were kept overnight in vacuum to ensure the evaporation of all solvent and subsequently hydrated. Acceptor vesicles were prepared in ultrapure H_2O containing 25 mM NaCl (lipid concentration: 10 mg/mL) by 1 h incubation at 50°C with intermittent vortex mixing and five freeze/thaw cycles. Donor vesicles were hydrated analogously, but in a 20 wt % sucrose solution (lipid concentration: 20 mg/mL). Acceptor vesicles were extruded at 50°C using a handheld mini extruder (Avanti Polar Lipids) with a 100 nm pore diameter polycarbonate filter 31 times or until reaching a polydispersity index <10%. The latter parameter was monitored by dynamic light scattering using a Zetasizer NANO ZS90, (Malvern Panalytical, Malvern, UK) ensuring the exclusive presence of LUVs. Multilamellar vesicles (MLVs) of donor lipids were diluted 20-fold with water and centrifuged at 20,000 $\times$ g for 30 min. The supernatant was discarded. The resulting pellet was suspended in a 35 mM mβCD solution (lipid:mβCD 1:8 mol/mol) and incubated for 2 h at 50°C, while being shaken at a frequency of 600 min^{-1}. Acceptor vesicles were added and incubated for another 15 min. The exchange was stopped by diluting the mixture eightfold with water and centrifuging at 20,000 $\times$ g for 30 min. The supernatant containing the asymmetric vesicles was then concentrated to <500 μL using 15 mL Amicon centrifuge filters (Merck, 100 kDa cutoff) at 5000 $\times$ g. To remove residual mβCD and sucrose, and to exchange H_2O by D_2O, filters were filled with D_2O and reconcentrated in three cycles. The final vesicle sizes were again measured by dynamic light scattering to ensure the absence of donor MLVs. The observed size increase of aLUVs (Table S1) indicates increased membrane tension due to the presence of salt in the vesicle lumen. However, the calculated change of membrane tension ($\sim 10^{-4}$ mN/m) is too small to affect the experimental membrane properties reported here, such as the bilayer thickness or bending rigidity; for details see the supporting material.

Scrambled LUVs were produced by drying asymmetric vesicles in a rotary evaporator at 30°C and pressures around 50 Pa. The resulting films were thereafter rehydrated with D_2O and extruded as described above. Symmetric reference LUVs for inner and outer leaflets were prepared in pure D_2O analogously. Inner leaflet reference LUVs contained 90 mol % acceptor lipid and 10 mol % donor lipid, while outer leaflet

reference LUVs were mixtures of 30 mol % acceptor and 70 mol % donor lipid.

Compositional analysis using gas chromatography and high-performance thin-layer chromatography

The overall vesicle composition of all aLUVs—except for POPE^{in}/POPC^{out} and (POPE/POPS)^{in}/POPC^{out}—was determined by characterizing the fatty acid composition via GC. For this procedure, fatty acid methyl esters were prepared upon incubation with a methanolic-H_2SO_4 solvent mixture. Gas chromatography (GC) measurements were done using a GC 2010 Plus (Shimadzu, Kyoto, Japan), equipped with a split/splitless injector and an SGE BPX70-cyanopropyl polysilphenylene-siloxane column (25 m $\times$ 0.22 mm ID and 0.25 μm film thickness) as described in (22). For calibration, we used logarithmically spaced concentration series of DPPC, DOPC, ESM (mostly 16:0 acyl chains), and MSM (mostly 22:0, 23:0, and 24:0 acyl chains). Lipid composition was given by interpolating through these calibration points.

POPE^{in}/POPC^{out} and (POPE/POPS)^{in}/POPC^{out} aLUVs were analyzed by HPTLC. After lipid extraction against organic solvent—$CHCl_3$/MeOH 2:1 (vol/vol)—samples were spotted on silica plates (Sigma-Aldrich, Steinheim, Germany) with the automatic TLC sampler 4 (CAMAG, Switzerland). The mobile phase in the developing chamber was a solvent mixture composed of 32.5:12.5:2 (vol/vol/vol) $CHCl_3$/MeOH/H_2O. After drying, the plate was immersed in a developing bath (5.08 g $MnCl_2$ dissolved in 480 mL H_2O, 480 mL EtOH, and 32 mL H_2SO_4), which is sensitive to double bonds, and dried for 15 min at 120°C. To quantify the lipid concentrations, the plate was scanned with the TLC scanner 3 (CAMAG) and further analyzed with WinCats software.

Measuring asymmetry via NMR spectroscopy

We used ^{1}H-NMR spectroscopy to determine the ratio of donor lipids in inner and outer leaflets by exposing the aLUVs to the shift reagent Pr^{3+} as detailed previously (20). Choline, which is present in all headgroups of donor lipids used here, gives a strong ^{1}H-NMR signal, which is shifted if the headgroup is surrounded by Pr^{3+}. Adding the reagent after sample preparation affects only outer leaflet headgroups within the timescale of the experiment and thereby allows us to discriminate against the signal of inner leaflet choline lipids. Experiments were performed on an Avance III 600 MHz spectrometer (Bruker, Billerica, MA) using the Bruker TopSpin acquisition software. A ^{1}H pulse sequence including water suppression (23) was used to collect 64 transients at 50°C. Data were processed with TopSpin 3.6 applying a line-broadening parameter of 1.5 Hz. In contrast to former measurements, we did not shift the outer leaflet peak to a certain position, but added enough Pr^{3+} to decrease its intensity far below those of its neighboring peaks (4 μL of a 20 mM Pr^{3+}/D_2O solution added to 500 μL lipid vesicle solution of about 0.5 mg/mL). The inside/outside ratio of donor lipid is given by the areas of the peak of the total cholines before and after adding the shift reagent; see the supporting material for more information.

SANS and SAXS

SANS measurements were performed at D22, Institut Laue-Langevin, Grenoble, France, and are available from DOI (10.5291/ILL-DATA.9-13-953, 10.5291/ILL-DATA.9-13-997). We used a multidetector setup with two ^{3}H-detectors, one of which was positioned at sample detector distances SDD = 5.6 or 17.8 m with collimations of 5.6 and 17.8 m, respectively, while the second detector was positioned out of center of the direct neutron beam at SDD = 1.3 m. The neutron wavelength was 6 Å ($\Delta\lambda/\lambda$ = 10%). Samples were filled in Hellma 120-QS cuvettes of 1 mm pathway and heated to 50°C using a water bath circulator. Lipid concentrations were about 5 mg/mL in 100% D_2O. Data reduction, including flat field, solid angle, dead time, and transmission corrections, intensity normalization by incident flux, and subtraction of contributions from empty cell and solvent were performed using GRASP (ILL).

SAXS data were recorded at BM29, ESRF, Grenoble, France, using a photon energy of 15 keV and a Pilatus3 2M detector at SDD = 2.867 m (data available at DOI: 10.15151/ESRF-ES-514136943). Samples were measured at a concentration of 10 mg/mL, at 50°C and exposed for 40 s (20 frames of 2 s each) in a flow-through quartz glass capillary (diameter: 1 mm). Data reduction and normalization was done by the automated ExiSAXS system, and SAXSutilities 2 (ESRF) was used for subtracting contributions from solvent and glass capillary.

SANS and SAXS data were analyzed jointly in terms of Gaussian-type volume distribution functions of quasimolecular lipid fragments across the bilayer, using our recently reported advanced scattering density model for aLUVs (12) (for details, see the supporting material). Compared with this previous study, we did not use lipids with deuterated hydrocarbons. Firstly, because our NSE experiments required fully protiated samples for optimal contrast, and, secondly, because of their limited commercial availability. Consequently we are not able to discriminate the structure of the individual leaflets. Structural parameters, such as the lateral area per lipid, A, are thus averaged over both leaflets. The chain thickness D_C of each leaflet was estimated from the position of the terminal methyl group. Note that this might not necessarily correspond to the center at the membrane, especially for samples containing the highly chain-asymmetric MSM (12).

Data were fitted following (24), but additionally constraining lipid composition of inner and outer leaflets with results from GC, HPTLC, and ^{1}H-NMR experiments as described by Eicher et al. (25). Furthermore, we fixed the distance between the hydrophobic interface and the backbone d_{BB} = 0.9 Å, the width of the Gaussians describing backbone σ_{BB} = 2.1 Å and the outermost headgroup (choline/amine) σ_{Chol} = 3 Å, and the smearing of the error function describing the hydrophobic-hydrophilic interface σ_{CH2} = 2.5 Å. Volumes of the individual moieties of the lipids were taken from (26). In the case of lipid mixtures, these values were calculated using molecular averaging and the experimentally determined estimates of leaflet composition (see results).

NSE

NSE measurements were performed at the IN15 spin-echo spectrometer, Institut Laue-Langevin, Grenoble, France (data available at DOI: 10.5291/ILL-DATA.TEST-3125, 10.5291/ILL-DATA.9-13-953, 10.5291/ILL-DATA.9-13-997) (27). We recorded NSE data in the range of q = 0.020 to 0.111 Å^{-1} using λ = 8, 10, and 12 Å. Lipid dispersions (concentration ~15 mg/mL) were filled into quartz cells of 1 mm pathlength. The accessible Fourier times ranged, depending on wavelength, from 0.01 to 300 ns. All experiments were performed at 50°C.

NSE data were analyzed in terms of the Zilman-Granek (ZG) theory (28) following the approach of Gupta et al. (29). In brief, the mean-squared displacement (MSD), $\langle \Delta r(t)^2 \rangle$, of the membrane from a flat geometry is related to the intermediate scattering function $S(q,t)$ assuming a Gaussian distribution of fluctuations, which at the same time is required to follow the ZG model

$$\frac{S(q,t)}{S(q)} \propto \exp\left[-\frac{q^2\langle \Delta r(t)^2 \rangle}{6}\right] \overset{!}{=} \exp\left[-\left(\Gamma_q t\right)^{2/3}\right], \quad (1)$$

where $S(q) = S(q,0)$ is the elastic structure factor. The decay constant Γ_q is connected to the “effective” or “dynamic” bending rigidity $\tilde{\kappa}$.

$$\Gamma_q = 0.025\gamma \frac{k_B T}{\eta}\sqrt{\frac{k_B T}{\tilde{\kappa}}} q^3, \quad (2)$$

where $\gamma \approx 1$ for $\tilde{\kappa} \gg k_B T$ (28), η is the solvent viscosity (η_{D_2O} (50°C) = 0.652×10^{-3} [Ns/m^2] (30)), k_B is Boltzmann’s constant, and T is the

TABLE 1 Composition and structure of presently studied aLUVs and scrambled LUVs

	Composition[a]			aLUV		Scrambled		
	χ^{total}	χ^{in}	χ^{out}	A_{av} (Å^2)[b]	D_B (Å)[c]	A (Å^2)[b]	D_B (Å)[c]	$\Delta A/A$ (%)[d]
POPE^{in}/ESM^{out}	61:39	98:02	28:72	64.0	37.6	61.8	38.2	3.6
POPE^{in}/MSM^{out}	67:33	82:18	54:46	61.4	40.3	63.8	39.0	−3.8
POPE^{in}/POPC^{out}	77:23	90:10	65:35	62.0	39.0	64.9	37.4	−4.5
POPE^{in}/(POPC/MSM)^{out}	52:48	65:35	40:60	62.9	39.6	65.0	38.4	−3.2
(POPE/POPS)^{in}/MSM^{out}	59:41	97:03	25:75	62.8	40.0	64.3	39.1	−2.3
(POPE/POPS)^{in}/POPC^{out}	71:29	97:03	48:52	66.0	37.2	65.8	36.7	0.3
(POPE/POPS)^{in}/(POPC/MSM)^{out}	62:38	77:23	49:51	63.4	39.1	66.9	37.1	−5.2

[a]χ, molar ratio between acceptor and donor lipids.
[b]A, lateral area per lipid. Average between inner and outer leaflet for aLUVs. Estimated relative uncertainty, 2%.
[c]D_B, Luzzati bilayer thickness. Estimated relative uncertainty: 2–3%.
[d]$\Delta A/A = (A_{av}^{aLUV} - A^{scrambled})/A^{scrambled}$. Estimated absolute uncertainty: 4%.

absolute temperature. The advantage of the MSD approach is the ability to discriminate against deviations from the ZG prediction for the decay of $S(q,t)/S(q)$ either due to non-Gaussian fluctuations occurring at short Fourier times, or due to vesicle diffusion dynamics, which dominate current data at long Fourier times (29). This approach avoids any "artificial" scaling by particle diffusion or interfacial viscosity (Fig. S3), which has led to significant controversies about bending rigidity values κ on absolute scale reported from NSE (31).

After ensuring the proper Fourier time range for the application of the ZG model we derived κ following the model of Watson and Brown (32). This theory takes internal dissipation into account and arrives at $\tilde{\kappa} = \kappa + 2h^2k_m$, where h is the position of the neutral surface and k_m is the monolayer area compressibility modulus. Furthermore, using $k_m = 12\kappa_m/D_C^2$, as predicted by the polymer brush model (21), and $\kappa_m = \kappa/2$ (33) for each leaflet, one arrives at

$$\kappa = \left[1 + 48(h/2D_C)^2\right]^{-1}\tilde{\kappa} \quad (3)$$

The exact position of h is not entirely clear and $h/2D_C$ and different groups report values between 0.25 and 0.6 (34). Following our work on intrinsic lipid curvatures (35) we assume that the neutral surface coincides with the lipid's backbone and calculate $h/(2D_C)$ from the results of our SAXS/SANS SDP analysis.

RESULTS

Transbilayer asymmetry and structure

We prepared a set of seven differently composed aLUVs using cyclodextrin-mediated exchange. Two different acceptor vesicles, POPE and POPE/POPS (7:3 mol/mol), were prepared. The latter mixture mimics to first order the natural composition of the inner leaflet of mammalian plasma membranes (2). Exchange was performed with four different donor MLVs: MSM, ESM, POPC, and POPC/MSM (1:1 mol/mol), the latter of which mimics the outer leaflet of plasma membranes. Furthermore, comparing MSM and ESM allowed us to probe the effects of chain length distribution in sphingolipids. The achieved total exchange and asymmetries are detailed in Table 1. Overall, exchange of the outer leaflet showed significant variations and was highest for POPE^{in}/POPC^{out}, where 65 mol % of acceptor lipid was replaced, a value which agrees well with a previous report (7). Exchange was in general low for both sphingolipids (<30 mol %). This might also affect the molecular ratio of aLUVs containing POPC/MSM mixtures, i.e., the final POPC/MSM molar ratios might be <1. Note that ^{1}H-NMR experiments performed on the chosen lipid moieties cannot be used to differentiate between phosphatidylcholines and sphingolipids. Such an analysis would require the application of headgroup deuterated species (36), which is beyond the scope of this study.

In the next step, we determined the corresponding membrane structural parameters using the joint analysis of SANS and SAXS data (Fig. 1). All experiments were performed at 50°C to ensure that both leaflets were well within the fluid lamellar phase. The transbilayer structure was parsed into the volume distributions of phosphate (PCN), choline-CH_3 (CholCH3), glycerol or sphingosine backbone (BB), methylene/methine (chains), and methyl (CH3) groups, including also bound water in the headgroup region (12). The applied model is in principle capable of deriving leaflet-specific structural details (12). However, the structural resolution of the individual leaflets is poor in the absence of deuterated phospholipids. Deriving leaflet-specific structural details is specifically challenged by the fact that the location of the terminal methyl groups—especially for the highly chain asymmetric MSM—might not be exactly at the center of an asymmetric bilayer (12,24). Therefore, we report the areas per lipid averaged over both leaflets, A_{av}, for aLUVs in Table 1; nonaveraged, leaflet-specific structural data are listed in Table S3 (fits are shown in Fig. S5). In addition, we report in Table 1 the corresponding structural parameters for scrambled vesicles, that is LUVs with the same, but symmetric lipid composition (fits to SANS/SAXS data are shown in Figs. S10–S16 and structural details in Tables S6–S8). We note deviations in the SAXS data fits of some samples at very low scattering angles. The origin of these deviations is most likely due to issues with assigning proper contrast in the headgroup regime. However, because membrane structure dominates scattering at higher q, the impact of

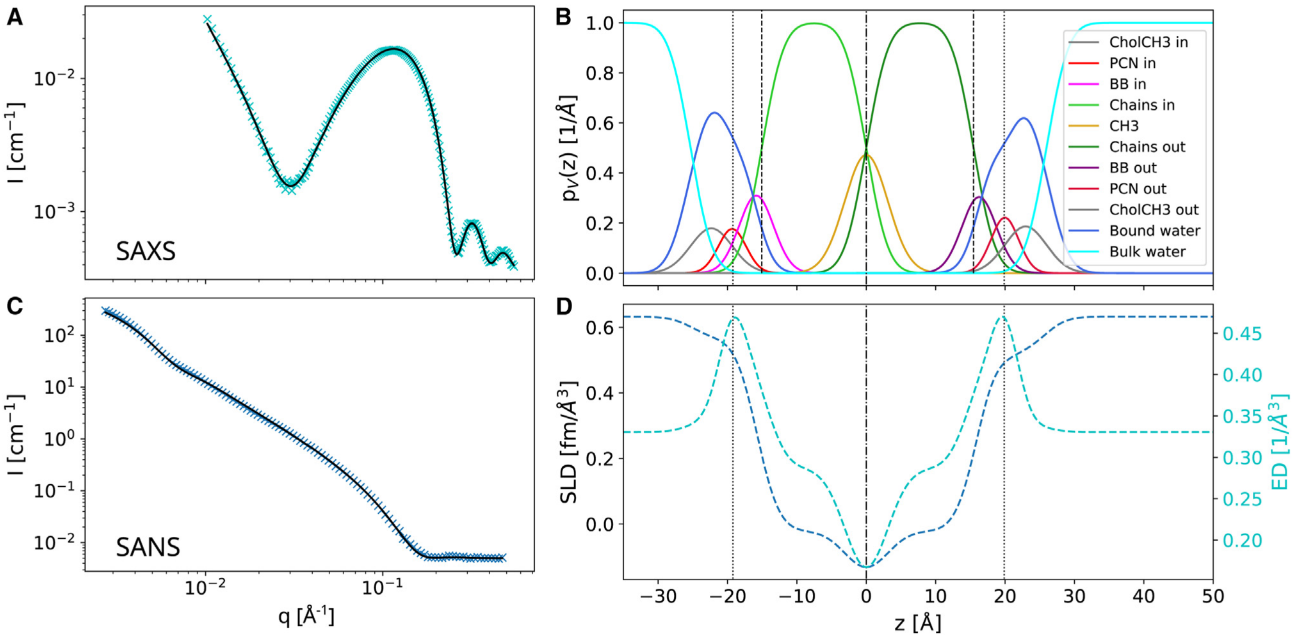

FIGURE 1 Structure of (POPE/POPS)^{in}/(MSM/POPC)^{out} aLUVs as revealed by joint SAXS/SANS data analysis. (*A* and *C*) Fits (*black lines*) to SAXS and SANS data (50°C). (*B*) The derived volume probability profiles. (*D*) The neutron scattering length density (SLD) and electron density (ED). To see this figure in color, go online.

this mismatch at low q on the derived structural parameters is rather low (see also (24) for more details).

To facilitate comparison between aLUVs and scrambled LUVs we additionally report the relative change in lipid area $\Delta A/A = (A_{av}^{aLUV} - A^{scrambled})/A^{scrambled}$ in Table 1. Overall, we observe that aLUVs are laterally more condensed than their scrambled analogs. Exceptions to this finding are POPE^{in}/ESM^{out} with $\Delta A/A > 0$ and (POPE/POPS)^{in}/POPC^{out} bilayers ($\Delta A/A \sim 0$).

Bending elasticity

NSE experiments were performed on the same exact samples studied by SANS/SAXS to avoid potential artifacts from sample history, e.g., sample-to-sample variations of lipid exchange efficiencies. The principles of the applied analysis are demonstrated in Fig. 2 for POPE^{in}/POPC^{out} aLUVs. The MSD plot shows an overlap of data between $t = 5 - 100$ ns and q values between ~ 0.07 and 0.11 Å^{-1}, all following a $t^{2/3}$ slope as predicted by the ZG theory (28). Data that do not follow the ZG theory, i.e., deviate from the $t^{2/3}$ slope, have been omitted from further analysis (Fig. 2). Fig. 2 also demonstrates the sensitivity of the technique to fluctuation differences between symmetric and asymmetric bilayers.

We first tested our NSE analysis using DPPC LUVs at 50°C as a benchmark system. This not only allows us to compare our analysis with previously reported bending rigidity data of DPPC, but also provides us with an internal standard to observe relative changes for all our other samples. Deriving the membrane bending rigidity requires, according to Eq. 3, the neutral plane, h, and the hydrocarbon thickness, D_C. Assuming that h coincides with the position of the glycerol backbone (35), we arrive at $h/(2D_C) = 0.53 \pm 0.07$ using previously reported data for DPPC bilayers (12). This result is well within the range of previously reported values for $h/(2D_C)$ (34). Application of Eq. 3 then leads to $\kappa = 12.0 \pm 0.4$ k$_B$T, which is almost three times lower than the bending rigidity of DPPC reported from other techniques (37). Note that alternative NSE evaluations of the same data also do not yield satisfactory agreement with literature values (Fig. S3). It has been suggested that including a separate tilt modulus in the NSE data analysis should be considered to harmonize κ values from NSE with other techniques (38). Indeed, Guler et al. (39) reported $\kappa \sim 15.0$ k$_B$T for DPPC—a value that is very close to our present result—using x-ray scattering, without correcting for the tilt modulus. Only later was this value corrected to $\kappa \sim 30$ k$_B$T by including the tilt modulus in the analysis (37). Revising the theory for NSE to bring κ to correct absolute scale levels is beyond the scope of the present study. Here, it suffices to note that NSE is sensitive to relative changes of undulatory motions (see also insert to Fig. 2). We will thus discuss only the relative differences of κ between the studied samples.

Symmetric references of inner and outer leaflets

We performed additional NSE experiments on symmetric control samples composed of POPC, MSM, ESM and

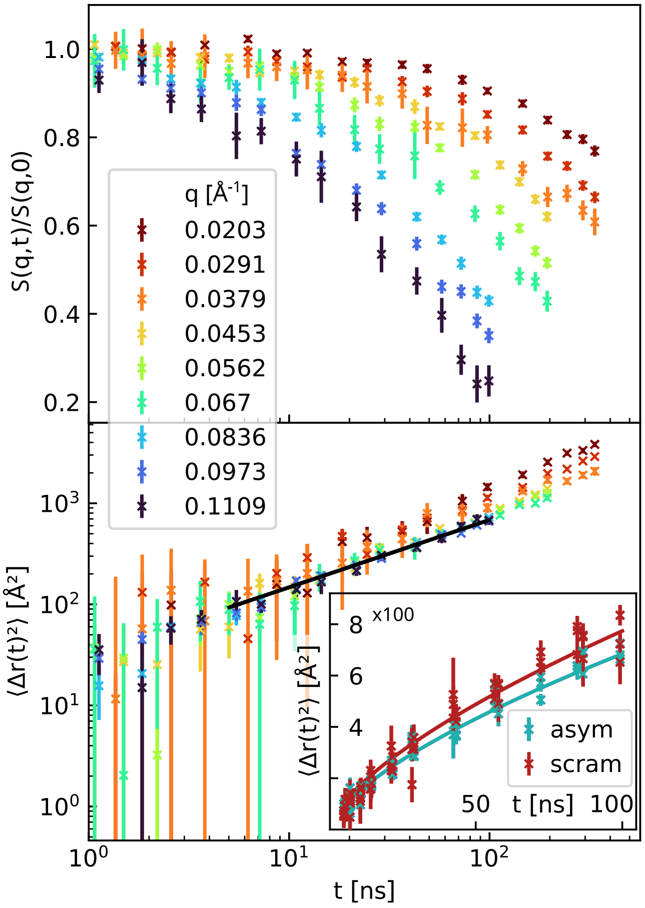

FIGURE 2 Principles of NSE data analysis. The upper panel shows raw NSE of POPE^{in}/POPC^{out} aLUVs. Data below 5 ns were used to normalize $S(q,t)/S(q,0)$ and have been omitted in part in the plot due to experimental uncertainties. The lower panel presents the corresponding MSD (*lower panel*). The solid black line marks the range fitting the $t^{2/3}$-slope predicted by the Zilman-Granek model, and which is used for the bending rigidity analysis. The insert compares the MSD data of the aLUVs with scrambled vesicles of the same lipid composition and demonstrates the sensitivity of the technique to differences in fluctuations dynamics. Errors correspond to experimentsal uncertainties. To see this figure in color, go online.

POPE, or POPE/POPS (7:3 mol/mol) mixtures. These samples allowed us to estimate the bending rigidities of each leaflet for the aLUVs in the absence of any coupling mechanism (see below).

Table 2 summarizes the corresponding major results from SANS/SAXS and NSE experiments (see also Figs. S10–S16and Tables S6–S8). We briefly note some interesting elastic properties of these simple lipid systems. Firstly, the bending rigidity of POPC is lower than that of DPPC despite its about equal membrane thickness (see the head-to-headgroup distances D_{HH}, reported in Table S5). This is surprising in view of the well-accepted polymer brush model (21), which predicts $\kappa \propto t_m^2$, where t_m is the mechanical thickness of the membrane, which is frequently set equal to D_{HH}. A recent computational approach showed that t_m can be smaller than D_{HH} for unsaturated hydrocarbons (40), which might explain our findings. Secondly, the bending rigidities of both sphingolipids are markedly higher than all other presently studied lipids. This indicates additional contributions due to the different backbone structure and the lipid's ability to form intermolecular H-bonds. Thirdly, the addition of 30 mol % POPS to POPE increases the lateral area per lipid (Table S5), but does not lead to significant changes in κ. Possibly its concentration is still too low to cause membrane stiffening as reported for other charged bilayers (18,19).

The bending rigidities of symmetric vesicles composed just of inner leaflet or outer leaflet lipid mixtures, detailed in Table 1, can now be estimated by interpolating the data reported in Table 2. Alternatively, one could have measured just vesicles with the exact leaflet compositions of Table 1. However, lipid exchange is difficult to predict. Thus, additional NSE, SAXS, and SANS beamtimes would have been needed, just to measure the properties of these control samples. Our solution thus was to interpolate data, which was additionally aided by measuring symmetric acceptor:donor lipid mixtures of 9:1 and 3:7 mol/mol. These compositions roughly correspond to the inner and outer leaflet compositions of the aLUVs. The bending rigidity of POPC/MSM equimolar mixtures was calculated from the molecular average of κ of the individual lipids. Plotting κ of symmetric vesicles as a function of lipid composition, we observed clear linear trends for most studied bilayers (Figs. 3 and S4), providing us with good confidence in the interpolated κ values of the inner and outer leaflet mimics controls for comparison with the elasticity of aLUVs.

Anomalous elasticity of asymmetric bilayers

Fig. 4 summarizes our results for κ values of all studied aLUVs and compares them with the bending rigidities of three different symmetric LUVs: scrambled vesicles, and their cognate inner and outer leaflets. The bending rigidities of symmetric cognate leaflet samples correspond to upper and lower boundary expectation values for each system. In the case of uncoupled lipid leaflets, κ of aLUVs should correspond to the arithmetic mean of these boundary values (8). Measurements of scrambled LUVs provide an additional control for the effect of lipid distribution across the bilayer on bending fluctuations.

Four aLUVs with inner leaflets enriched only in POPE were studied. These had outer leaflets with ESM, MSM, POPC, and equimolar mixtures of POPC and MSM. Differences in κ between asymmetric and scrambled membranes were negligible within experimental certainty for POPE/ESM and POPE/MSM vesicles. Furthermore, bending rigidities of these asymmetric membranes corresponded to the arithmetic averages of their cognate leaflet vesicles, indicating the absence of transbilayer elastic coupling. This seems to contrast a recent NSE study on POPE^{in}/ESM^{out}, which reported stiffer asymmetric bilayers compared with

TABLE 2 Structural and elastic properties of symmetric LUVs containing acceptor and donor lipids at 50°C

	$\frac{h}{2D_C}$[c]	κ (k_BT)
POPC[a]	0.53 ± 0.07	8.9 ± 0.5
MSM[a]	0.56 ± 0.08	22.4 ± 0.9
ESM	0.59 ± 0.08	16.5 ± 0.8
POPE	0.54 ± 0.08	8.5 ± 0.3
POPE/POPS[b]	0.59 ± 0.08	7.8 ± 0.5

[a]Structural data taken from (24).
[b]7:3 mol/mol.
[c]h, the height of the neutral plane; D_C, the hydrocarbon chain length.

their scrambled systems (17). However, the stiffening effect was mainly dominating at lower temperatures (30°C), i.e., within the chain-melting regime, and decreased upon increasing temperature to 45°C, the highest studied temperature by these authors. It thus appears that the stiffening effect is abolished upon further increasing temperature, i.e., when reaching the temperature of 50°C used here. The other two aLUVs with POPE-enriched inner leaflets behaved much differently. When either POPC or POPC/MSM mixtures were present in the outer leaflet, aLUVs were much stiffer than all other symmetric control LUVs, i.e., exceeding even the upper boundaries of inner/outer leaflet LUVs. The stiffening effect was slightly more expressed for POPE^{in}/POPC^{out}, with $\Delta\kappa_r \equiv \kappa^{aLUV}/\kappa^{scrambled} \sim 1.5$, whereas $\Delta\kappa_r \sim 1.4$ for POPE^{in}/(POPC/MSM)^{out}.

Asymmetric membranes with POPE/POPS mixtures showed even larger rigidity differences between aLUVs and scrambled vesicles. The largest relative bending changes between these two systems occurred again in the case of POPC outer leaflets with $\Delta\kappa_r \sim 2.2$, followed by (POPC/MSM)^{out} ($\Delta\kappa_r \sim 1.9$) and MSM^{out} ($\Delta\kappa_r \sim 1.7$). Note that the minor contamination of (POPE/POPS)^{in}/POPC^{out} samples with POPC MLVs, as observed by SAXS (Fig. S8), would rather skew the determined κ toward smaller values (see Table 2). Furthermore, the MSM^{out} aLUVs had an about equal bending rigidity than symmetric MSM-enriched outer leaflet vesicles. Asymmetric membranes with either POPC or POPC/MSM outer leaflets in turn revealed bending rigidities much outside the κ boundaries set by the symmetric cognate leaflet vesicles.

DISCUSSION

We observed anomalous stiffening for most of the presently studied asymmetric lipid membranes. Here, anomalous refers to bilayers, which are even stiffer than the upper rigidity boundary set by their symmetric cognate leaflet vesicles. A similar effect was reported previously for asymmetric giant unilamellar vesicles composed of POPC and DOPC (14), as well as DOPC and dimyristoylphosphatidylcholine (16). In turn, Rickeard et al. (17) found that the κ values of POPE/ESM aLUVs matched those of outer leaflet symmetric vesicles for fluid bilayers. Thus, the bending rigidity of these asymmetric membranes was not anomalously stiffer than the symmetric controls, but dominated by the more rigid leaflets. We found a complete loss of this mechanical coupling for the same asymmetric membranes at slightly higher temperatures (Fig. 4), most likely due to interactions of entropic origin. Similarly, POPE/MSM aLUVs showed no anomalous coupling of its leaflets. In stark contrast, POPE/POPC asymmetric bilayers were about 1.5 times more rigid than the cognate leaflet systems, clearly signifying anomalous membrane stiffening. This effect was enhanced for aLUVs with POPE/POPS-enriched inner leaflets, except for (POPE/POPS)^{in}/MSM^{out}, whose bending rigidity was dominated by the more rigid leaflet, following the above discussed behavior of POPE/ESM asymmetric membranes at lower temperatures (17).

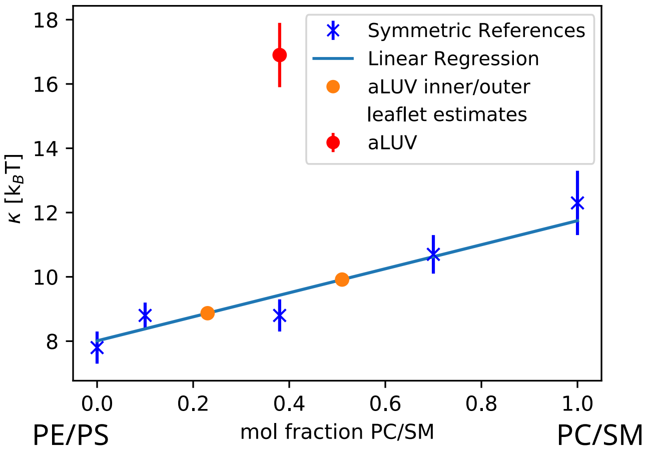

FIGURE 3 Variation of bending rigidity of symmetric POPE/POPS/POPC/MSM mixtures with POPC/MSM (1:1 mol/mol) concentration, abbreviated in the plot by PC/SM for clarity of presentation. A PC/SM mol fraction of 0 corresponds to POPE/POPS (7:3 mol/mol), here abbreviated by PE/PS. Orange circles represent κ estimates using the overall lipid compositions of Table 1. The red symbol shows κ of the corresponding aLUVs (see also Fig. 4). Errors correspond to uncertainties of the applied NSE data analysis. To see this figure in color, go online.

Attempting to reconcile our experimental observations within existing theoretical frameworks, we first checked whether the differences could be simply understood in terms of the polymer brush model (21), i.e., differences in membrane thickness. Indeed, we observed a thickening of aLUVs for most of the presently considered systems, which would agree with a decreased bending flexibility (Table 1). Assuming that the area expansion modulus is not affected by membrane asymmetry, $\Delta\kappa_r$, determined above from NSE experiments, can also be estimated using the D_{HH} values reported in Tables S5, S7, and S8. This yields $\Delta\kappa_r$ values between 0.9 and 1.2 for the presently studied samples, but with no correlation to the $\Delta\kappa_r$ values found from NSE data (Fig. 4). This indicates that the area expansion modulus of asymmetric membranes differs significantly from that of scrambled vesicles, which would agree with a previous report (16).

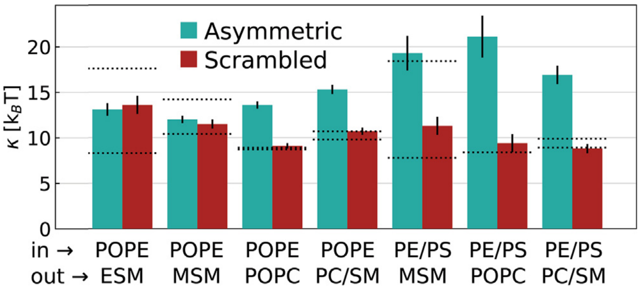

FIGURE 4 Bending rigidities κ measured by NSE for asymmetric and physically scrambled vesicles. Dotted lines correspond to the reference samples of inner and outer leaflet composition. The labels indicate the predominant lipids in inner and outer leaflets. The following mixtures have been abbreviated for clarity of display: PE/PS, POPE/POPS (7:3 mol/mol); PC/SM, POPC/MSM (1:1 mol/mol). Errors correspond to uncertainties of the applied NSE data analysis.To see this figure in color, go online.

Hossein and Deserno (8,13) recently predicted a stiffening transition in asymmetric membranes based on differential stress (nonvanishing lateral tension) between the two leaflets. Such differential stress can arise either from compositional asymmetry, or leaflet under/overcrowding (number asymmetry). The latter type of asymmetry is difficult to control experimentally. Experimental studies using cyclodextrin-mediated lipid exchange, including this one, typically report some amounts of donor lipid within the inner leaflet (7,12,17,36,41). This suggests a rapid equilibration of any differential stress resulting from number asymmetry. We note that anomalous stiffening for asymmetric bilayers was also reported using a very different sample preparation technique (14,16). Anomalous membrane stiffening thus is unlikely a manufacturing artifact. On quantitative grounds, we can use the area per lipid determined by SANS/SAXS as indicators of leaflet under/overcrowding. Table 1 reports the relative area per lipid changes between asymmetric and scrambled vesicles. In the case of significant stress due to lateral area mismatch between leaflets in asymmetric membranes, we would expect also to observe corresponding area per lipid changes upon lipid scrambling due to stress relaxation. Variations of $\Delta A/A$ are, however, within a few percent, and do not correlate with the observed effects on bending rigidity. Hence, we can rule out membrane stiffening arising from number asymmetry.

Focusing next on compositional asymmetry, we estimate the differential stress in flat membranes (8).

$$\sigma_d = \frac{\overline{\kappa} J_{0b}}{2h}, \tag{4}$$

where $J_{0,b}$ is the rigidity-weighted difference of the outer leaflet J_0^{OL} and inner leaflet J_0^{IL} spontaneous curvatures and $\overline{\kappa} = \kappa_m^{\mathrm{OL}} + \kappa_m^{\mathrm{IL}}$ is the leaflet averaged bending rigidity. Table 3 shows the results using previously reported spontaneous monolayer data (42,43). The calculated $J_{0,b}$ values are all close to 0 and, with the exception of $(\mathrm{POPE/POPS})^{\mathrm{in}}$/$(\mathrm{POPC/MSM})^{\mathrm{out}}$, all have positive signs. Consequently, also differential stresses are rather small, with absolute values varying between 0.16 and 0.95 mN/m. Correcting our κ for contributions of the tilt modulus (see above) would increase the reported σ_d values, but by not more than a factor of 3, i.e., still yielding rather small σ_d values. Thus, contributions of compositional asymmetry to differential stress remain negligible, mostly due to the small $J_{0,b}$ values. Moreover, relative changes of σ_d also do not explain the variation of κ for different samples and anomalous stiffening (Fig. 4). In particular, aLUVs with the highest κ are among those with lowest $|\sigma_d|$.

For the sake of the argument—and in the absence of any theory we are aware of—let us, therefore, speculate on the putative role of fluctuations. The undulatory motions of the fluctuation spectrum of lipid membranes comprise bending and protrusion modes (44,45). Here, we probed bending modes, which dominate the fluctuation spectrum for distances larger than the membrane thickness. At shorter distances, these interleaflet dynamics are thought to be essentially decoupled. In this regime shorter wavelength fluctuations (alternatively hard modes) are dominated by single lipid motions (e.g., protrusions). While this theory agrees well with large-scale molecular dynamics simulations of symmetric bilayers (44,45), it is less clear if it applies also to asymmetric membranes. For instance, our data might suggest that fluctuations in some of our aLUVs are coupled even at distances smaller than the membrane thickness, increasing the weight of “hard” modes on bending fluctuations. This enhanced intraleaflet coupling appears to particularly occur for POPC-enriched outer leaflets. Both POPE and POPS carry primary amines in their headgroups, enabling intermolecular H-bond formation, which is indeed known to stabilize bilayer structure (46). The headgroup charge of POPS additionally seems to accentuate the intraleaflet coupling, possibly by giving rise to a transmembrane potential, or by increasing contributions from surface tension. It should be noted that interactions, particularly between charged lipids, depend strongly on ionic strength and may differ between the low-salt conditions used here and physiological conditions in an organism. It is therefore desirable to address the effect of ionic strength and pH in detail in a subsequent study.

Interestingly, no anomalous elastic coupling was observed for ESM- and MSM-enriched outer leaflets, and the stiffening effect was partially reduced for POPC/MSM mixtures. ESM and MSM are natural lipid extracts, containing also some long acyl chains. In particular, MSM is highly chain-asymmetric with mostly 22:0, 23:0, and 24:0 hydrocarbons and thus prone to interdigitation (12). The hydrocarbon chain composition of ESM is dominated by 16:0 hydrocarbons but also contains a few longer hydrocarbons that can interdigitate into the opposing leaflet. Hydrocarbon interdigitation of POPC in turn is negligible (24). Consequently, our data suggest that the anomalous mechanical coupling induced by the

TABLE 3 Elastic leaflet properties and resulting theoretical rigidity-weighted spontaneous curvature and differential stress for aLUVs

	J_0^{IL} (nm^{-1})[a]	J_0^{OL} (nm^{-1})[a]	κ_m^{IL} (k_BT)[b]	κ_m^{OL} (k_BT)[b]	h (Å)[c]	J_{0b} (nm^{-1})	σ_d (mN/m)[d]
POPE^{in}/ESM^{out}	−0.28	−0.06	4.2	7.5	16.9	0.06	0.96
POPE^{in}/MSM^{out}	−0.23	−0.14	5.3	5.3	16.8	0.05	0.63
POPE^{in}/POPC^{out}	−0.25	−0.17	4.4	4.5	16.0	0.04	0.47
POPE^{in}/(POPC/MSM)^{out}	−0.17	−0.09	5	5.5	16.3	0.03	0.49
(POPE/POPS)^{in}/MSM^{out}	−0.21	−0.08	3.9	9.2	16.6	0.01	0.11
(POPE/POPS)^{in}/POPC^{out}	−0.21	−0.15	4.2	4.2	15.2	0.03	0.37
(POPE/POPS)^{in}/(POPC/MSM)^{out}	−0.14	−0.15	4.5	5	16.1	−0.01	−0.17

[a]Monolayer spontaneous curvature. Data taken from (42,43) and averaged according to molecular leaflet composition. Intrinsic curvatures of MSM, ESM, and POPS were assumed to be zero.
[b]Monolayer bending rigidity. Calculated from symmetric reference LUVs for inner leaflets (IL) and outer leaflets (OL) using $\kappa_m = \kappa/2$.
[c]Height of the neutral plane. Average value for both leaflets.
[d]Differential stress.

enrichment of aminophospholipid in one leaflet can be alleviated by hydrocarbon interdigitation.

The potential biological relevance is as intriguing as our findings. Several cellular processes, occurring either at large scales, such as the formation of exo- or endosomes, or within the single molecular regime, e.g., transport or signaling events, depend on membrane elasticity (4,5). By tuning the leaflet distribution of lipids in given regions of the plasma membrane through an orchestrated operation of flipase/flopase and scramblase cells might control its elasticity over a broad range of bending rigidities and exploit its effect on certain cellular processes. Indeed, a highly enhanced deformability of plasma membranes of *Drosophila* cells was recently attributed to the activity of a dedicated scramblase (47). Further scrutiny of the systems reported here, including cholesterol and different buffer conditions, is challenging but certainly warranted. Furthermore, including sufficient amounts of cholesterol would also allow for lowering the temperature to more physiologically relevant conditions, while remaining within the fluid phase for both lipid leaflets.

CONCLUSION

We observed that asymmetric transbilayer distribution of the aminophospholipids POPE and POPS can lead to an anomalous stiffening of membranes, i.e., exceeding not only the bending rigidity of their scrambled analogs but also that of their cognate leaflets in symmetric bilayers. These effects can be alleviated at least in part by the addition of sphingomyelin in the outer leaflet, possibly due to hydrocarbon chain interdigitation. We speculate that an asymmetric distribution of lipids with specific properties (charge, H-bonding abilities) leads to enhanced mechanical coupling of membranes, by increasing the weight of hard undulatory modes through intraleaflet interactions. This strongly encourages theoretical and computational studies along those directions. Although the presently studied systems still lack important components of mammalian plasma membranes (e.g., cholesterol) it is plausible that cells have evolved to a state where they can exploit a large range of bending rigidities for protein function by dynamically modulating leaflet-specific lipid composition.

SUPPORTING MATERIAL

Supporting material can be found online at https://doi.org/10.1016/j.bpj.2023.04.025.

AUTHOR CONTRIBUTIONS

M.P.K.F., Y.G., L.P., and G.P. designed the research. M.P.K.F., P.P., E.F.S., and O.C. carried out all experiments. M.P.K.F. analyzed the data. M.P.K.F. and G.P. wrote the article.

ACKNOWLEDGMENTS

This research was supported by the ILL graduate school, Ph.D. no. 181-19. P.P. and E.F.S. are supported by the Austrian Science Fund, grant no. P32514 (to G.P.). We thank the ILL and the ESRF for beamtime, the PSCM for access to support laboratories, and Petra Pernot, Mark Tully, Dihia Moussaoui, and Anton Popov for their assistance with experiments at BM29 and Krishna Batchu for help with GC measurements. We thank E. London, H. Heerklotz, F. Heberle, M. Doktorova, and M. Deserno for constructive discussions.

DECLARATION OF INTERESTS

The authors declare no competing interests.

SUPPORTING CITATIONS

References (48–51) appear in the supporting material.

REFERENCES

1. Verkleij, A. J., R. F. Zwaal, …, L. L. van Deenen. 1973. The asymmetric distribution of phospholipids in the human red cell membrane. A combined study using phospholipases and freeze-etch electron microscopy. *Biochim. Biophys. Acta.* 323:178–193.

2. Lorent, J. H., K. R. Levental, …, I. Levental. 2020. Plasma membranes are asymmetric in lipid unsaturation, packing and protein shape. *Nat. Chem. Biol.* 16:644–652. https://doi.org/10.1038/s41589-020-0529-6.
3. Van Meer, G. 2011. Dynamic transbilayer lipid asymmetry. *Cold Spring Harb. Perspect. Biol.* 3:a004671. http://cshperspectives.cshlp.org/content/3/5/a004671.full.pdf#page=1&view=FitH.
4. Doktorova, M., J. L. Symons, and I. Levental. 2020. Structural and functional consequences of reversible lipid asymmetry in living membranes. *Nat. Chem. Biol.* 16:1321–1330. http://www.nature.com/articles/s41589-020-00688-0.
5. Tiberti, M. L., B. Antonny, and R. Gautier. 2020. The transbilayer distribution of polyunsaturated phospholipids determines their facilitating effect on membrane deformation. *Soft Matter.* 16:1722–1730.
6. Nickels, J. D., J. C. Smith, and X. Cheng. 2015. Lateral organization, bilayer asymmetry, and inter-leaflet coupling of biological membranes. *Chem. Phys. Lipids.* 192:87–99.
7. Eicher, B., D. Marquardt, …, G. Pabst. 2018. Intrinsic curvature-mediated transbilayer coupling in asymmetric lipid vesicles. *Biophys. J.* 114:146–157. https://doi.org/10.1016/j.bpj.2017.11.009.
8. Hossein, A., and M. Deserno. 2020. Spontaneous curvature, differential stress, and bending modulus of asymmetric lipid membranes. *Biophys. J.* 118:624–642.
9. Kiessling, V., C. Wan, and L. K. Tamm. 2009. Domain coupling in asymmetric lipid bilayers. *Biochim. Biophys. Acta.* 1788:64–71.
10. Enoki, T. A., and G. W. Feigenson. 2022. Improving our picture of the plasma membrane: rafts induce ordered domains in a simplified model cytoplasmic leaflet. *Biochim. Biophys. Acta.* 1864, 183995. https://www.sciencedirect.com/science/article/pii/S000527362200133X.
11. Chiantia, S., and E. London. 2012. Acyl chain length and saturation modulate interleaflet coupling in asymmetric bilayers: effects on dynamics and structural order. *Biophys. J.* 103:2311–2319.
12. Frewein, M. P. K., P. Piller, …, G. Pabst. 2022. Interdigitation-induced order and disorder in asymmetric membranes. *J. Membr. Biol.*. https://link.springer.com/10.1007/s00232-022-00234-0.
13. Hossein, A., and M. Deserno. 2021. Stiffening transition in asymmetric lipid bilayers: the role of highly ordered domains and the effect of temperature and size. *J. Chem. Phys.* 154, 014704.
14. Elani, Y., S. Purushothaman, …, O. Ces. 2015. Measurements of the effect of membrane asymmetry on the mechanical properties of lipid bilayers. *Chem. Commun.* 51:6976–6979. http://pubs.rsc.org/en/content/articlepdf/2015/cc/c5cc00712g.
15. Karamdad, K., R. V. Law, …, O. Ces. 2016. Studying the effects of asymmetry on the bending rigidity of lipid membranes formed by microfluidics. *Chem. Commun.* 52:5277–5280. http://xlink.rsc.org/?DOI=C5CC10307J.
16. Lu, L., W. J. Doak, …, P. R. Chiarot. 2016. Membrane mechanical properties of synthetic asymmetric phospholipid vesicles. *Soft Matter.* 12:7521–7528. http://xlink.rsc.org/?DOI=C6SM01349J.
17. Rickeard, B. W., M. H. L. Nguyen, …, D. Marquardt. 2020. Transverse lipid organization dictates bending fluctuations in model plasma membranes. *Nanoscale.* 12:1438–1447.
18. Lu, B.-S., S. P. Gupta, …, G. Pabst. 2016. Modulation of elasticity and interactions in charged lipid multibilayers: monovalent salt solutions. *Langmuir.* 32:13546–13555.
19. Faizi, H. A., S. L. Frey, …, P. M. Vlahovska. 2019. Bending rigidity of charged lipid bilayer membranes. *Soft Matter.* 15:6006–6013.
20. Doktorova, M., F. A. Heberle, …, D. Marquardt. 2018. Preparation of asymmetric phospholipid vesicles for use as cell membrane models. *Nat. Protoc.* 13:2086–2101. https://doi.org/10.1038/s41596-018-0033-6.
21. Rawicz, W., K. C. Olbrich, …, E. Evans. 2000. Effect of chain length and unsaturation on elasticity of lipid bilayers. *Biophys. J.* 79:328–339. https://linkinghub.elsevier.com/retrieve/pii/S0006349500762953.
22. Marx, L., M. P. K. Frewein, …, G. Pabst. 2021. Antimicrobial peptide activity in asymmetric bacterial membrane mimics. *Faraday Discuss.* 232:435–447. http://pubs.rsc.org/en/Content/ArticleLanding/2021/FD/D1FD00039J.
23. Favier, A., and B. Brutscher. 2019. NMRlib: user-friendly pulse sequence tools for Bruker NMR spectrometers. *J. Biomol. NMR.* 73:199–211.
24. Frewein, M. P. K., M. Doktorova, …, G. Pabst. 2021. Structure and interdigitation of chain-asymmetric phosphatidylcholines and milk sphingomyelin in the fluid phase. *Symmetry.* 13:1441. https://www.mdpi.com/2073-8994/13/8/1441.
25. Eicher, B., F. A. Heberle, …, G. Pabst. 2017. Joint small-angle X-ray and neutron scattering data analysis of asymmetric lipid vesicles. *J. Appl. Crystallogr.* 50:419–429.
26. Nagle, J. F., R. M. Venable, …, R. W. Pastor. 2019. Revisiting volumes of lipid components in bilayers. *J. Phys. Chem. B.* 123:2697–2709.
27. Schleger, P., B. Alefeld, …, D. Richter. 1997. The long-wavelength neutron spin-echo spectrometer IN15 at the Institut Laue-Langevin. *Phys. B Condens. Matter.* 241-243:164–165.
28. Zilman, A. G., and R. Granek. 1996. Undulations and dynamic structure factor of membranes. *Phys. Rev. Lett.* 77:4788–4791. https://doi.org/10.1103/PhysRevLett.77.4788.
29. Gupta, S., J. U. De Mel, …, G. J. Schneider. 2018. Dynamics of phospholipid membranes beyond thermal undulations. *J. Phys. Chem. Lett.* 9:2956–2960.
30. Cho, C. H., J. Urquidi, …, G. W. Robinson. 1999. Thermal offset viscosities of liquid H2O, D2O, and T2O. *J. Phys. Chem. B.* 103:1991–1994.
31. Gupta, S., and G. J. Schneider. 2020. Modeling the dynamics of phospholipids in the fluid phase of liposomes. *Soft Matter.* 16:3245–3256.
32. Watson, M. C., and F. L. H. Brown. 2010. Interpreting membrane scattering experiments at the mesoscale: the contribution of dissipation within the bilayer. *Biophys. J.* 98:L9–L11.
33. Shearman, G. C., O. Ces, …, J. M. Seddon. 2006. Inverse lyotropic phases of lipids and membrane curvature. *J. Phys. Condens. Matter.* 18:S1105–S1124.
34. Nagao, M., E. G. Kelley, …, P. D. Butler. 2017. Probing elastic and viscous properties of phospholipid bilayers using neutron spin echo spectroscopy. *J. Phys. Chem. Lett.* 8:4679–4684. https://doi.org/10.1021/acs.jpclett.7b01830.
35. Kollmitzer, B., P. Heftberger, …, G. Pabst. 2013. Monolayer spontaneous curvature of raft-forming membrane lipids. *Soft Matter.* 9:10877–10884.
36. Heberle, F. A., D. Marquardt, …, G. Pabst. 2016. Subnanometer structure of an asymmetric model membrane: interleaflet coupling influences domain properties. *Langmuir.* 32:5195–5200.
37. Nagle, J. F. 2017. Experimentally determined tilt and bending moduli of single-component lipid bilayers. *Chem. Phys. Lipids.* 205:18–24. https://linkinghub.elsevier.com/retrieve/pii/S0009308417300427.
38. Nagle, J. F. 2021. Measuring the bending modulus of lipid bilayers with cholesterol. *Phys. Rev. E.* 104, 044405.
39. Guler, S. D., D. D. Ghosh, …, S. Tristram-Nagle. 2009. Effects of ether vs. ester linkage on lipid bilayer structure and water permeability. *Chem. Phys. Lipids.* 160:33–44.
40. Doktorova, M., M. V. LeVine, …, H. Weinstein. 2019. A new computational method for membrane compressibility: bilayer mechanical thickness revisited. *Biophys. J.* 117:502–790. https://linkinghub.elsevier.com/retrieve/pii/S0006349518345302.
41. Marquardt, D., F. A. Heberle, …, G. Pabst. 2017. 1H NMR shows slow phospholipid flip-flop in gel and fluid bilayers. *Langmuir.* 33:3731–3741.
42. Frewein, M. P. K., M. Rumetshofer, and G. Pabst. 2019. Global small-angle scattering data analysis of inverted hexagonal phases. *J. Appl. Crystallogr.* 52:403–414. http://scripts.iucr.org/cgi-bin/paper?S1600576719002760.
43. Kaltenegger, M., J. Kremser, …, G. Pabst. 2021. Intrinsic lipid curvatures of mammalian plasma membrane outer leaflet lipids and ceramides. *Biochim. Biophys. Acta. Biomembr.* 1863:183709.
44. Goetz, R., G. Gompper, and R. Lipowsky. 1999. Mobility and elasticity of self-assembled membranes. *Phys. Rev. Lett.* 82:221–224.

45. Lindahl, E., and O. Edholm. 2000. Mesoscopic undulations and thickness fluctuations in lipid bilayers from molecular dynamics simulations. *Biophys. J.* 79:426–433.

46. Marsh, D. 2013. Handbook of Lipid Bilayers, Second edition. CRC Press.

47. Shiomi, A., K. Nagao, …, M. Umeda. 2021. Extreme deformability of insect cell membranes is governed by phospholipid scrambling. *Cell Rep.* 35, 109219.

48. Imran, A., D. Popescu, and L. Movileanu. 2020. Cyclic activity of an osmotically stressed liposome in a finite hypotonic environment. *Langmuir.* 36:3659–3666.

49. Reddy, A. S., D. T. Warshaviak, and M. Chachisvilis. 2012. Effect of membrane tension on the physical properties of DOPC lipid bilayer membrane. *Biochim. Biophys. Acta.* 1818:2271–2281.

50. Gracià, R. S., N. Bezlyepkina, …, R. Dimova. 2010. Effect of cholesterol on the rigidity of saturated and unsaturated membranes: fluctuation and electrodeformation analysis of giant vesicles. *Soft Matter.* 6:1472–1482. https://pubs.rsc.org/en/content/articlelanding/2010/sm/b920629a.

51. Kučerka, N., J. Pencer, …, J. Katsaras. 2007. Curvature effect on the structure of phospholipid bilayers. *Langmuir.* 23:1292–1299.

Biophysical Journal, Volume *122*

Supplemental information

Distributing aminophospholipids asymmetrically across leaflets causes anomalous membrane stiffening

Moritz P.K. Frewein, Paulina Piller, Enrico F. Semeraro, Orsolya Czakkel, Yuri Gerelli, Lionel Porcar, and Georg Pabst

Supplement: Aminophospholipids Stiffen Membranes When Populating Their Inner Leaflets

Moritz PK Frewein, Paulina Piller, Enrico F Semeraro,
Orsolya Czakkel, Yuri Gerelli, Lionel Porcar and Georg Pabst

Contents

1 Effect of hypotonic conditions on membrane tension 2

2 Evaluation of NMR data 3

3 SDP-model used for SAS-analysis 4

4 The contribution of diffusion in NSE-data modelling 7

5 Bending Rigidities of Lipid mixtures 8

6 SAS-model parameters of donor/acceptor lipids 9

7 SAS-model parameters of asymmetric vesicles 11

8 SAS-model parameters of reference LUVs 16

1 Effect of hypotonic conditions on membrane tension

Preparation of acceptor vesicles in 25 mM NaCl and washing in pure water after CD-mediated lipid exchange creates an osmotic imbalance between the lumen of the aLUVs and the bulk water solution, which potentially leads to a surface tension that affects bending rigidity measurements. In the following, we estimate this effect.

Lipid vesicles are known to swell under hypotonic conditions until reaching a critical tension beyond which pores or defects may form to relax the stress [1]. At lower tension and in equilibrium, the osmotic pressure created by the osmolytes

$$P_{osm} = kTN_A \Delta c_s \tag{1}$$

balances the Laplace pressure

$$P_L = 2\sigma/R, \tag{2}$$

where k is Boltzmann's constant, N_A is Avogadro's constant, Δc_s is the concentration difference of the solute between the lumen of the vesicle and bulk solution, σ is the surface tension and R is the vesicle radius. The change in membrane tension due to swelling of the vesicle is easily calculated from the change of vesicle size.

$$\Delta\sigma = \frac{kTN_A\Delta c_s}{2}\Delta R. \tag{3}$$

We observed an increase of vesicle radii between 0 and 18 nm (Tab. S1) by DLS. The highest value appears to be an outlier due to the presence of a minor population of donor vesicles; see also (Fig. S8). Thus, using $\Delta c_s = 25$ mM and $\Delta R = 3$ nm (average size increase, excluding the outlier) we calculate $\Delta\sigma \approx 10^{-4}$ mN/m.

We can estimate the effect of the calculated membrane tension change on the membrane thickness, d_B. Molecular dynamics simulations of dioleoyl phosphatidylcholine reported $\frac{\Delta d_B}{\Delta\sigma} \approx -1.33 \times 10^3$ Å/(mN/m) [2]. The expected change of membrane thickness for $\Delta\sigma \approx 10^{-4}$ mN/m, therefore, is ≈ -0.1 Å, i.e. within our present experimental uncertainty of SAXS/SANS (consistent with our observations). Membrane tension does affect the undulation amplitudes of vesicles, which might couple for sufficiently high values to the bending rigidity, κ [3]. However, the calculated tension change is well within the low-tension regime (< 0.5 mN/m), where κ remains constant and enables its measurement via micropipette pressurization of giant bilayer vesicles [4]. Thus, we can neglect any systematic increase of bending rigidity originating from our aLUV preparation.

Table S1: Vesicle radii for acceptor vesicles, R_{av}^{acc}, and aLUVs, R_{av}^{asym} from DLS experiments (experimental uncertainty: ±3 nm). Experiments and data analysis were done using a Malvern Zetasizer Nano ZS90 and the software supplied by the manufacturer (Malvern Panalytical, Malvern, UK). PDI denotes the polydispersity index of the measured size distribution.

Sample	R_{av}^{acc} [nm]	PDI [%]	R_{av}^{asym} [nm]	PDI [%]	ΔR [nm]
POPE^{in}/ESM^{out}	61	6	63	11	2
POPE^{in}/MSM^{out}	55	10	58	18	3
POPE^{in}/POPC^{out}	55	10	64	30	9
POPE^{in}/(POPC/MSM)^{out}	55	10	58	17	3
(POPE/POPS)^{in}/MSM^{out}	65	15	65	6	0
(POPE/POPS)^{in}/POPC^{out}	65	15	83	30	18[a]
(POPE/POPS)^{in}/(POPC/MSM)^{out}	65	15	68	13	3

[a] minor donor contamination (see also Fig. S8)

2 Evaluation of NMR data

Figure S1 shows an exemplary NMR spectrum of asymmetric vesicles before and after addition of Pr^{3+}. The areas A_0 and A_1 under the Lorentzian fits of the peak at 3.4 ppm (dotted lines) correspond to the total amount of choline headgroups (before) and the amount of choline lipids inside the vesicle (after), respectively. The mol-fraction of PC-lipids (POPC and/or Sphingomyelin) in the inner leaflet is therefore given by $\chi_P^{in} = A_1/A_0$.

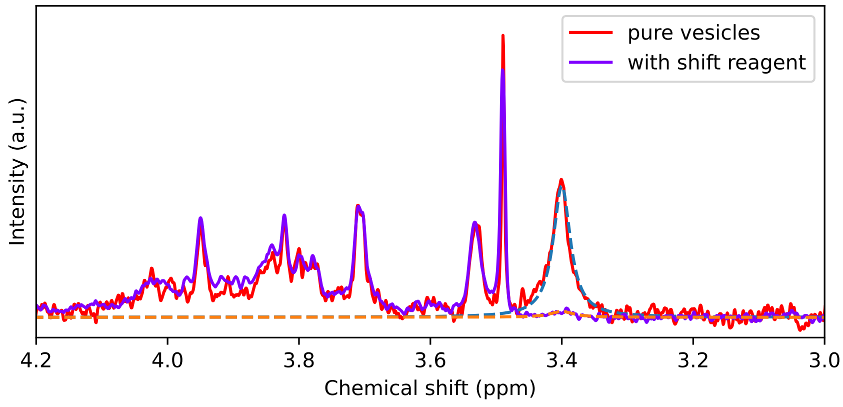

Figure S1: The graph shows a part of the NMR spectrum of asymmetric vesicles before and after adding Pr^{3+}. While the majority of the choline peak is moved from its original position (3.4 ppm), the rest of the spectrum is unaffected. Dotted lines mark Lorentzians used to fit the peaks and to calculate the underlying areas.

3 SDP-model used for SAS-analysis

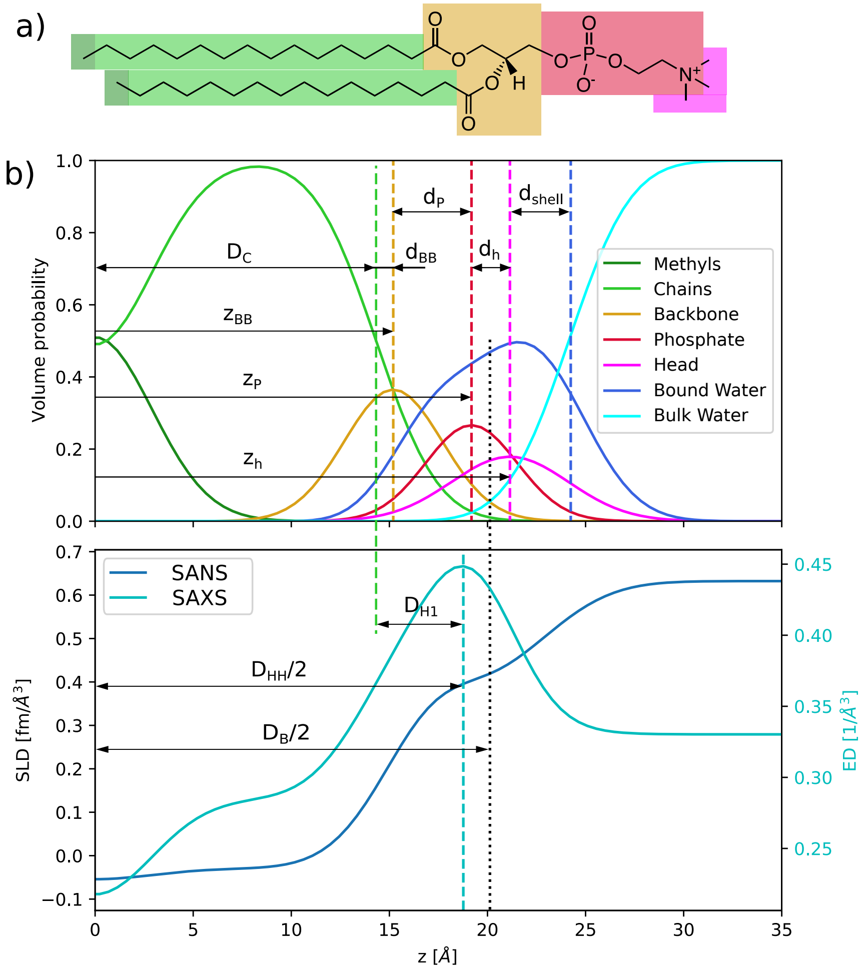

Figure S2: Lipid parsing and scattering length density profile modelling: a) The upper graphic shows how a lipid is divided in fragments on the example of DPPC. The parsing off all other used lipids can be found in Table S2. b) The color-coded arrangement of functions (*pdf*) in the upper diagram corresponds to the probability density to find a fragment at a distance z from the bilayer center. The *pdf*s for the terminal methyls (T), lipid backbones (BB), phosphate groups (P) and heads (h) are modelled by Gaussian functions. Hydrocarbon chains (HC) and a hydration water layer (bound water, BW) are slabs with altitude 1 and smeared with error-functions. From these, the functions of T and $BB + P + H$ are substracted respectively. The *pdf* of the solvent (bulk water W) fills the remaining space, so that the sum of all *pdf*s is 1 at all z. The lower diagram shows the neutron scattering length density (SLD) and electron density (ED) profiles of the modelled lipid in D_2O. It is obtained from the sum over all *pdf*s, multiplied with the respective SLD or ED.

Table S2: List of lipid fragments and chemical compositions for all used lipids. X denote exchangable hydrogens, which are replaced with deuterium in a D_2O-environment.

Fragment	Terminal methyls	Chains without T	Backbone	Phosphate group	Head
Abbr.	T	HC	BB	P	h
DPPC	$(CH_3)_2$	$(CH_2)_{28}$	$C_5O_4H_5$	$PO_4(CH_2)_2N$	$(CH_3)_3$
DPPG	$(CH_3)_2$	$(CH_2)_{28}$	$C_5O_4H_5$	PO_4	$C_3H_5O_2X_2$
POPE	$(CH_3)_2$	$(CH_2)_{28}(CH)_2$	$C_5O_4H_5$	PO_4	$(CH_2)_2NX_3$
POPG	$(CH_3)_2$	$(CH_2)_{28}(CH)_2$	$C_5O_4H_5$	PO_4	$C_3H_5O_2X_2$
POPS	$(CH_3)_2$	$(CH_2)_{28}(CH)_2$	$C_5O_4H_5$	PO_4	$C_3H_2X_3NO_2H$
POPC	$(CH_3)_2$	$(CH_2)_{28}(CH)_2$	$C_5O_4H_5$	$PO_4(CH_2)_2N$	$(CH_3)_3$
ESM	$(CH_3)_2$	$(CH_2)_{26.6}(CH)_2$	$C_4O_2NH_6$	$PO_4(CH_2)_2N$	$(CH_3)_3$
MSM	$(CH_3)_2$	$(CH_2)_{32}(CH)_2$	$C_4O_2NH_6$	$PO_4(CH_2)_2N$	$(CH_3)_3$

SAS-data are modelled using the vesicle form factor F_{sphere}, the bilayer form F_{bil}, which we split into real and imaginary part, and the incoherent background I_{inc}. Furthermore, we applied a Gaussian polydispersity on the hydrophobic thickness D_C, which results in a weighted average over several bilayer form factors $F_{bil,k}$. A more detailed description of the model was published earlier [5]. Due to the lack of contrast between both leaflets, we are not able to locate the bilayer center by SANS and therefore fixed the position of the terminal methyl group $z_T = 0$ in this study. For all symmetric references, the imaginary part of the bilayer form factor is equal 0 and only one leaflet has to be modelled. All quantities $\Delta\rho$ denote the scattering length density contrast of moiety k with respect to the water/heavy water environment $\Delta\rho_k = \rho_k - \rho_W$. Distances are defined in Fig. S2, where D_B is defined as the Luzzati bilayer thickness and D_{HH} the distance between the maxima in the ED-profile. $D_{H1} = (D_B - D_{HH})/2$. σ_k are the standard deviations of the respective *pdf*s. d_{shell} was fixed to 3.1 Å for all samples. Further parameters: A area per lipid; V_L total lipid volume; V_H total head group volume (back bone, phosphate group and head); $r_{BB} = V_{BB}/V_H$, $r_P = V_P/V_H$, $r = V_{CH3}/V_{CH2}$, $r_{12} = V_{CH}/V_{CH2}$; $V_{W,bound}$ volume per bound water molecule; n_W number of bound water molecules; Y relative interdigitation (see [6]).

$$I(q) \;\propto\; F_{sphere}(R_m, \sigma_R) \sum_k \mathcal{N}(D_{C,k}|\bar{D_C}, \sigma_{\mathrm{poly}}) \left\{ |F^{real}_{bil,k}|^2 + |F^{imag}_{bil,k}|^2 | \right\} + I_{inc} \tag{4}$$

with

$$\begin{aligned}
F^{real}_{bil,k} =& 4\Delta\rho_{\mathrm{bw}} \frac{1}{q} \mathrm{e}^{-\frac{q^2 \sigma^{\mathrm{in}\,2}_{\mathrm{CH2}}}{2}} \sin\left(q \frac{d^{\mathrm{in}}_{\mathrm{BB}} + d^{\mathrm{in}}_P + d^{\mathrm{in}}_{shell}}{2} \right) \\
& \cos\left(q(-D^{\mathrm{in}}_{C,k} - \frac{d^{\mathrm{in}}_{\mathrm{BB}} + d^{\mathrm{in}}_P d^{\mathrm{in}}_{shell}}{2}) \right) + \\
& 2(\Delta\rho^{\mathrm{in}}_P - \Delta\rho_{\mathrm{bw}}) \frac{V^{in}_P}{A} \mathrm{e}^{-\frac{q^2 \sigma^{\mathrm{in}\,2}_P}{2}} \cos\left(q(-D^{\mathrm{in}}_{C,k} - d^{\mathrm{in}}_{\mathrm{BB}} - d^{\mathrm{in}}_P/2) \right) + \\
& 2(\Delta\rho^{\mathrm{in}}_{\mathrm{BB}} - \Delta\rho_{\mathrm{bw}}) \frac{V^{\mathrm{in}}_{\mathrm{BB}}}{A} \mathrm{e}^{-\frac{q^2 \sigma^{\mathrm{in}}_{\mathrm{BB}}{}^2}{2}} \cos\left(q(-D^{\mathrm{in}}_{C,k} - d^{\mathrm{in}}_{\mathrm{BB}}/2) \right) + \\
& 2\Delta\rho^{\mathrm{in}}_{\mathrm{CH2}} \frac{1}{q} \mathrm{e}^{-\frac{q^2 \sigma^{\mathrm{in}}_{\mathrm{CH2}}{}^2}{2}} \sin\left(q D^{\mathrm{in}}_{C,k}/2 \right) \cos\left(q(-D^{\mathrm{in}}_{C,k}/2) \right) + \\
& 2\Delta\rho_{\mathrm{CH2}} \frac{1}{q} \mathrm{e}^{-\frac{q^2 \sigma^2_{\mathrm{CH2}}}{2}} \sin\left(q D^{\mathrm{out}}_{C,k}/2 \right) \cos\left(q D^{\mathrm{out}}_{C,k}/2 \right) + \\
& 2(\Delta\rho^{\mathrm{out}}_{\mathrm{BB}} - \Delta\rho_{\mathrm{bw}}) \frac{V^{\mathrm{out}}_{\mathrm{BB}}}{A} \mathrm{e}^{-\frac{q^2 \sigma^{\mathrm{out}\,2}_{\mathrm{BB}}}{2}} \cos\left(q(D^{\mathrm{out}}_{C,k} + d^{\mathrm{out}}_{\mathrm{BB}}/2) \right) + \\
& 2(\Delta\rho^{\mathrm{out}}_P - \Delta\rho_{\mathrm{bw}}) \frac{V^{\mathrm{out}}_P}{A} \mathrm{e}^{-\frac{q^2 \sigma^{\mathrm{out}\,2}_P}{2}} \cos\left(q(D^{\mathrm{out}}_{C,k} + d^{\mathrm{out}}_{\mathrm{BB}} + d^{\mathrm{out}}_P/2) \right) + \\
& 4\Delta\rho_{\mathrm{bw}} \frac{1}{q} \mathrm{e}^{-\frac{q^2 \sigma^{\mathrm{out}}_{\mathrm{CH2}}{}^2}{2}} \sin\left(q \frac{d^{\mathrm{out}}_{\mathrm{BB}} + d^{\mathrm{out}}_P + d^{\mathrm{out}}_{shell}}{2} \right) \\
& \cos\left(q(D^{\mathrm{out}}_{C,k} + \frac{d^{\mathrm{out}}_{\mathrm{BB}} + d^{\mathrm{out}}_P + d^{\mathrm{out}}_{shell}}{2}) \right)
\end{aligned} \tag{5}$$

and

$$
\begin{aligned}
F_{bil,k}^{imag} =& 4\Delta\rho_{\mathrm{bw}}\frac{1}{q}\mathrm{e}^{-\frac{q^2\sigma_{\mathrm{CH2}}^{\mathrm{in}\,2}}{2}}\sin\left(q\frac{d_{\mathrm{BB}}^{\mathrm{in}}+d_{P}^{\mathrm{in}}+d_{shell}^{\mathrm{in}}}{2}\right) \\
& \sin\left(q(-D_{C,k}^{\mathrm{in}}-\frac{d_{\mathrm{BB}}^{\mathrm{in}}+d_{P}^{\mathrm{in}}d_{shell}^{\mathrm{in}}}{2})\right)+ \\
& 2(\Delta\rho_{P}^{\mathrm{in}}-\Delta\rho_{\mathrm{bw}})\frac{V_{P}^{in}}{A}\mathrm{e}^{-\frac{q^2\sigma_{P}^{\mathrm{in}\,2}}{2}}\sin\left(q(-D_{C,k}^{\mathrm{in}}-d_{\mathrm{BB}}^{\mathrm{in}}-d_{P}^{\mathrm{in}}/2)\right)+ \\
& 2(\Delta\rho_{\mathrm{BB}}^{\mathrm{in}}-\Delta\rho_{\mathrm{bw}})\frac{V_{\mathrm{BB}}^{\mathrm{in}}}{A}\mathrm{e}^{-\frac{q^2\sigma_{\mathrm{BB}}^{\mathrm{in}\,2}}{2}}\sin\left(q(-D_{C,k}^{\mathrm{in}}-d_{\mathrm{BB}}^{\mathrm{in}}/2)\right)+ \\
& 2\Delta\rho_{\mathrm{CH2}}^{\mathrm{in}}\frac{1}{q}\mathrm{e}^{-\frac{q^2\sigma_{\mathrm{CH2}}^{\mathrm{in}\,2}}{2}}\sin\left(qD_{C,k}^{\mathrm{in}}/2\right)\sin\left(q(-D_{C,k}^{\mathrm{in}}/2\right)+ \\
& \frac{\Delta\rho_{\mathrm{T}}^{\mathrm{in}}+\Delta\rho_{\mathrm{T}}^{\mathrm{out}}}{2}\mathrm{e}^{-\frac{q^2\sigma_{\mathrm{T}}^2}{2}}+ \qquad (6) \\
& 2\Delta\rho_{\mathrm{CH2}}\frac{1}{q}\mathrm{e}^{-\frac{q^2\sigma_{\mathrm{CH2}}^2}{2}}\sin\left(qD_{C,k}^{\mathrm{out}}/2\right)\sin\left(qD_{C,k}^{\mathrm{out}}/2\right)+ \\
& 2(\Delta\rho_{\mathrm{BB}}^{\mathrm{out}}-\Delta\rho_{\mathrm{bw}})\frac{V_{\mathrm{BB}}^{\mathrm{out}}}{A}\mathrm{e}^{-\frac{q^2\sigma_{\mathrm{BB}}^{\mathrm{out}\,2}}{2}}\sin\left(q(D_{C,k}^{\mathrm{out}}+d_{\mathrm{BB}}^{\mathrm{out}}/2)\right)+ \\
& 2(\Delta\rho_{P}^{\mathrm{out}}-\Delta\rho_{\mathrm{bw}})\frac{V_{P}^{\mathrm{out}}}{A}\mathrm{e}^{-\frac{q^2\sigma_{P}^{\mathrm{out}\,2}}{2}}\sin\left(q(D_{C,k}^{\mathrm{out}}+d_{\mathrm{BB}}^{\mathrm{out}}+d_{P}^{\mathrm{out}}/2)\right)+ \\
& 4\Delta\rho_{\mathrm{bw}}\frac{1}{q}\mathrm{e}^{-\frac{q^2\sigma_{\mathrm{CH2}}^{\mathrm{out}\,2}}{2}}\sin\left(q\frac{d_{\mathrm{BB}}^{\mathrm{out}}+d_{P}^{\mathrm{out}}+d_{shell}^{\mathrm{out}}}{2}\right) \\
& \sin\left(q(D_{C,k}^{\mathrm{out}}+\frac{d_{\mathrm{BB}}^{\mathrm{out}}+d_{P}^{\mathrm{out}}+d_{shell}^{\mathrm{out}}}{2})\right)
\end{aligned}
$$

To describe the contribution from the overall vesicle shape we use the Schultz-distributed form factor of a sphere, as described in [7]:

$$
F_{sphere} = \frac{8\pi^2(z+1)(z+2)}{s^2q^2}\left\{1-\left(1+\frac{4q^2}{s^2}\right)^{-(z+3)/2}\cos\left[(z+3)\arctan\left(\frac{2q}{s}\right)\right]\right\} \qquad (7)
$$

Mean vesicle radius R_m and polydispersity σ_R enter via the auxiliary quantities $s = \frac{R_m}{\sigma_R^2}$ and $z = \frac{R_m^2}{\sigma_R^2} - 1$.

4 The contribution of diffusion in NSE-data modelling

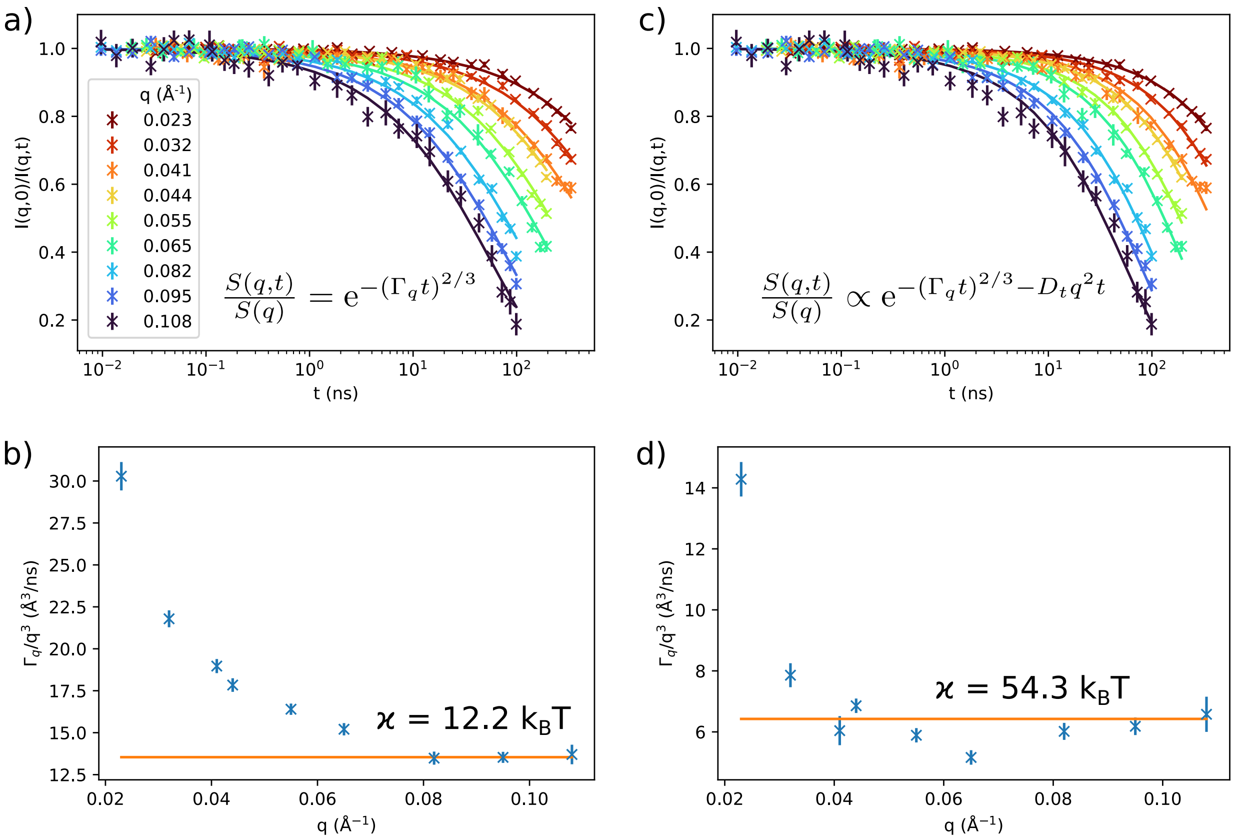

Figure S3: The graphic shows the comparison of 2 models used to evaluate NSE-data of DPPC-vesicles at 50 °C: (I) the pure Zilman-Granek (ZG) model (a,b) and (II) the ZG model with a contribution from diffusion (c,d), using a translational diffusion constant $D_t = 0.66$ Å^2/ns, measured by dynamic light scattering. The formulas to fit data are given in (a) and (c). Both models are in reasonable agreement with the raw data, however using model (I), the resulting values for the q-dependent decay constant Γ_q (b) do not follow q^3 as predicted by the ZG-theory. Model (II) improves the agreement over a wider q-range (d), but still deviates at low q. The given values for the bending rigidity κ in (b) and (d) correspond to the orange lines.

5 Bending Rigidities of Lipid mixtures

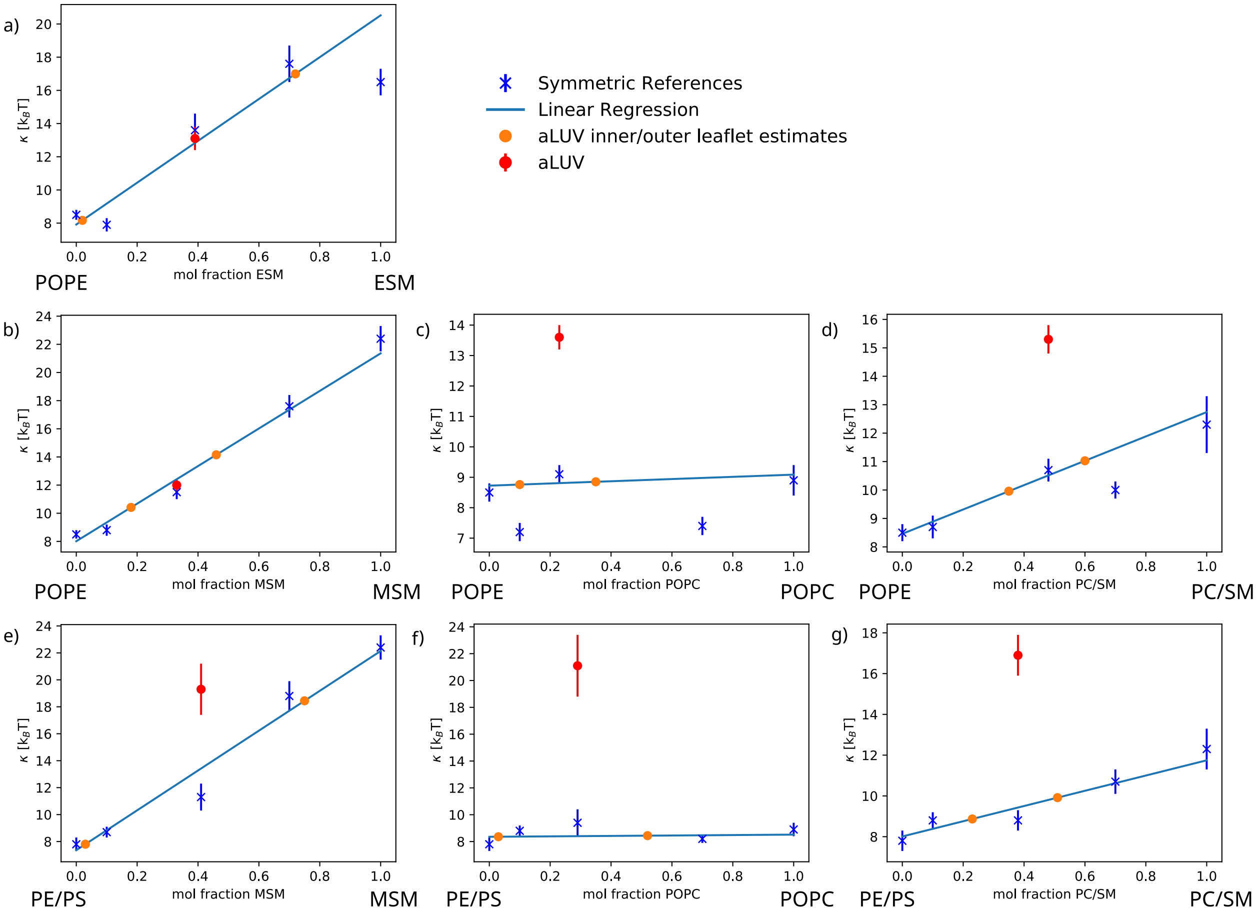

Figure S4: The graphic shows the measured bending rigidities κ for all aLUVs (red) as well as symmetric references (blue). We performed linear regressions through the reference samples and extrapolated to retrieve the κ at the measured inner/outer leaflet compositions (orange). Some points had to be excluded from the analysis due to large deviations from the linear law: a) $X_{ESM} = 0.1$ and $X_{ESM} = 1$, c) $X_{POPC} = 0.1$ and $X_{POPC} = 0.7$, d) $X_{PC/SM} = 0.7$, e) $X_{MSM} = 0.46$. The origin of these deviations is unclear.

6 SAS-model parameters of donor/acceptor lipids

Table S3: Summary of structural properties of symmetric LUVs at 50°C.

	A (Å^2)	D_{HH} (Å)[c]	$2D_C$ (Å)	h (Å)
DPPC[a]	63.1 ± 1.3	37.5 ± 1.1	28.6 ± 0.9	15.2 ± 1.2
POPC[a]	67.5 ± 1.4	37.5 ± 1.1	28.4 ± 0.9	14.8 ± 1.2
MSM[a]	64.8 ± 1.3	43.0 ± 1.1	32.8 ± 1.0	18.4 ± 1.5
ESM	56.2 ± 1.1	41.5 ± 1.1	33.6 ± 1.0	19.8 ± 1.6
POPE	60.5 ± 1.2	36.0 ± 1.1	31.2 ± 0.9	17.0 ± 1.4
POPE/POPS[b]	65.3 ± 1.3	37.1 ± 1.1	28.9 ± 0.9	17.0 ± 1.4

[a] Structural data taken from ref. [6].
[b] 7:3 mol/mol.
[c] head-to-headgroup distance.

Table S4: Properties of symmetric lipid bilayers from SAXS/SANS analysis at 50 °C containing POPE/POPG 9:1 (POPE), POPE/POPS 7:3 (PE/PS) and ESM/DPPG 19:1 (ESM).

	ϵ [%]	POPE	PE/PS	ESM
V_L^* [Å^3]		1193.8	1199.3	1218.5
V_H^* [Å^3]		249.6	254.9	274.9
r_{BB}^*		0.51	0.50	0.33
r_P^*		0.14	0.18	0.31
r^*		2.09	2.09	2.09
r_{12}^*		0.8	0.8	0.8
D_B [Å]	3	39.5	36.8	43.4
D_{HH} [Å]	3	36.0	37.1	41.5
$2D_C$ [Å]	3	31.2	28.9	33.6
D_{H1} [Å]	20	2.4	4.1	4.0
A [Å^2]	2	60.5	65.3	56.2
z_{BB} [Å]	8	17.0	17.0	19.8
σ_{BB} [Å]	20	2.5	2.5	3.2
z_P [Å]	8	18.8	20.0	21.1
σ_P [Å]	20	3.5	3.0	2.9
z_h [Å]	3	23.8	20.0	21
$\sigma_h^\dagger$ [Å]		2.8	3	3.0
σ_{HC} [Å]		2.5	2.5	2.5
σ_T [Å]	5	3.0	3.0	4.1
σ_{poly} [%]	6	4.0	8.1	3.5
$V_{W,bound}$ [Å^3]	6	29.6	29.4	27.3
n_W	6	13.8	10.0	4.4
Y	9	0.40	0.45	0.62

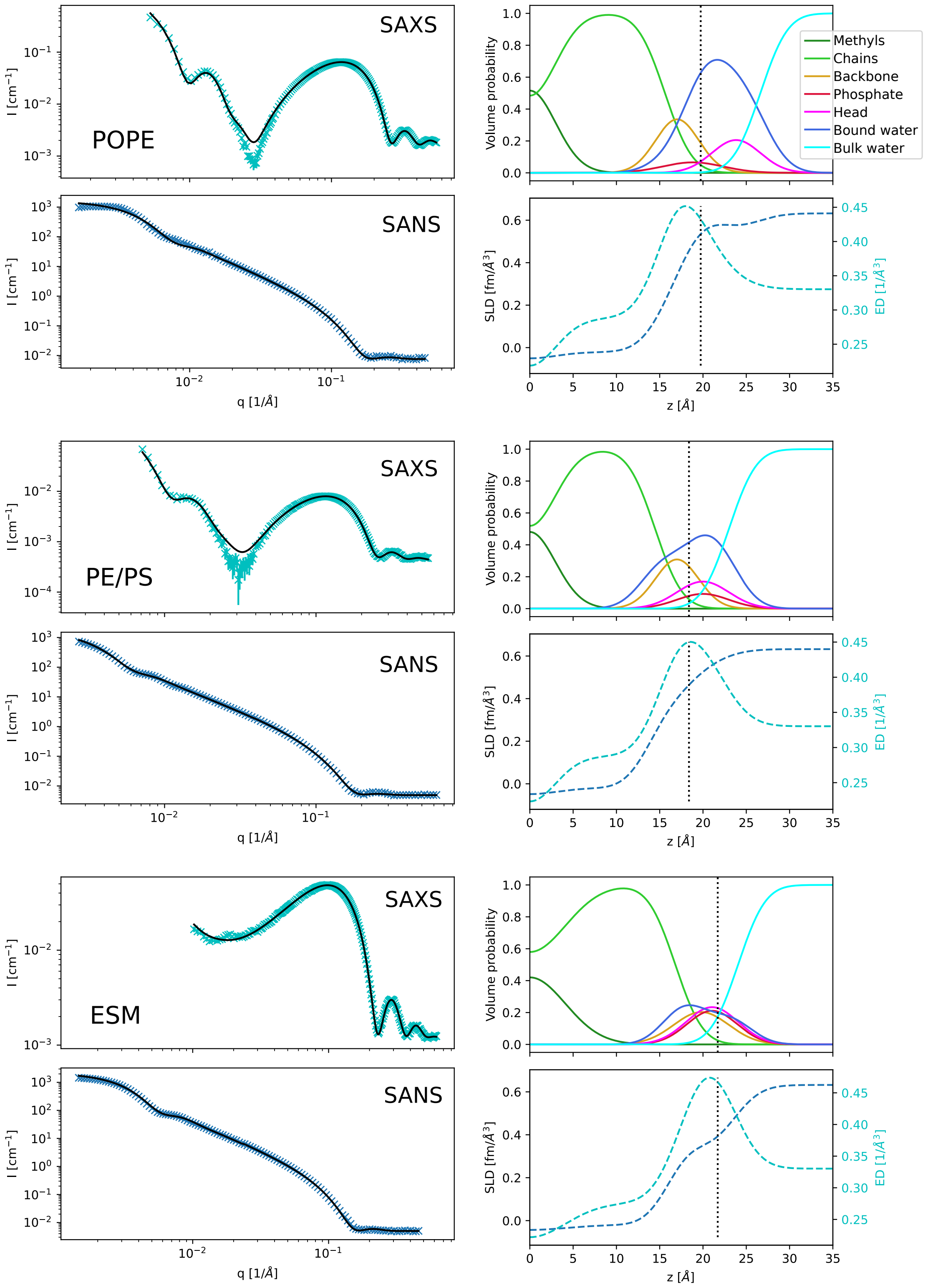

Figure S5: SAXS and SANS data with fits (black lines); SDP volume probability, electron density and neutron scattering length density profiles for symmetric lipid vesicles containing POPE/POPG 9:1, POPE/POPS 7:3 and Egg-SM/DPPG 19:1.

7 SAS-model parameters of asymmetric vesicles

Table S5: SAS-fitting parameters of aLUVs containing either a POPE/POPG 9:1 mixture (POPE) or a POPE/POPS 7:3 mixture (PE/PS) as acceptor lipids and ESM, POPC, MSM or a 1:1 POPC/MSM mixture (PC/SM) as donor lipids.

	ϵ [%]	POPE ESM	POPE POPC	POPE MSM	POPE PC/SM	PE/PS POPC	PE/PS MSM	PE/PS PC/SM
Total acc/don %	5	61:39	77:23	67:33	52:48	71:29	59:41	62:38
In acc/don %	5	98:2	90:10	82:18	65:35	97:3	97:3	77:23
Out acc/don %	5	28:72	65:35	54:46	40:60	48:52	25:75	49:51
D_B [Å]	3	37.6	39.0	40.3	39.6	37.1	40.0	39.1
D_{HH} [Å]	3	37.3	36.6	37.8	37.8	34.3	37.4	38.5
$2D_C$ [Å]	3	29.5	30.4	31.9	30.9	28.8	31.6	30.5
D_C^{in} [Å]	5	14.3	15.2	15.4	15.7	13.5	16.3	15.1
D_C^{out} [Å]	5	15.2	15.2	16.5	15.2	15.3	15.3	15.5
D_{H1}^{in} [Å]	20	4.6	2.8	3.3	3.8	2.3	3.4	3.9
D_{H1}^{out} [Å]	20	3.2	3.4	2.6	3.2	3.2	2.4	4.1
A^{av} [Å^2]	2	64.0	62.0	61.4	62.9	66.0	62.8	63.4
z_{BB}^{in} [Å]	6	-16.4	-16.0	-16.2	-16.5	-14.3	-17.1	-15.9
z_{BB}^{out} [Å]	6	17.4	16.0	17.3	16.0	16.1	16.1	16.3
$\sigma_{BB}^{in/out}$ [Å]		2.5	2.5	2.5	2.5	2.5	2.5	2.5
z_P^{in} [Å]	10	-19.4	-21.0	-19.2	-21.2	-17.3	-20.1	-19.3
z_P^{out} [Å]	10	22.4	19.0	21.5	19.0	19.7	19.9	20
σ_P^{in} [Å]	10	2.0	3.5	2	3.0	4.0	2.0	2
σ_P^{out} [Å]	10	4.0	2.0	3.5	2.3	3.0	3.8	2
z_h^{in} [Å]	10	-21.7	-24.0	-22.2	-24.2	-20.3	-23.1	-22.3
z_h^{out} [Å]	10	24.6	22.0	24.5	22.0	22.7	22.9	23
$\sigma_h^{in/out\dagger}$ [Å]		3.0	3.0	3	3.0	3.0	3.0	3
$\sigma_{HC}^{in/out}$ [Å]		2.5	2.5	2.5	2.5	2.5	2.5	2.5
σ_T [Å]	20	3.7	3.0	3.4	3.7	5.0	3.6	3.1
σ_{poly} [%]	6	0.0	3.9	4.3	5.0	10.0	7.0	7
$V_{W,bound}$ [Å^3]	6	30.3	30.3	30.2	30.0	29.4	29.6	29.3
n_W^{in}	6	14.4	15.4	11.8	14.6	14.2	10.3	12.8
n_W^{out}	6	16.7	11.0	13.4	11.5	11.6	14.8	12.7
R_m [Å]	10	361	361	345.3	373.4	369	361.2	381.9
σ_R [Å]	10	133	133	101.4	111.7	116.1	133	134.7

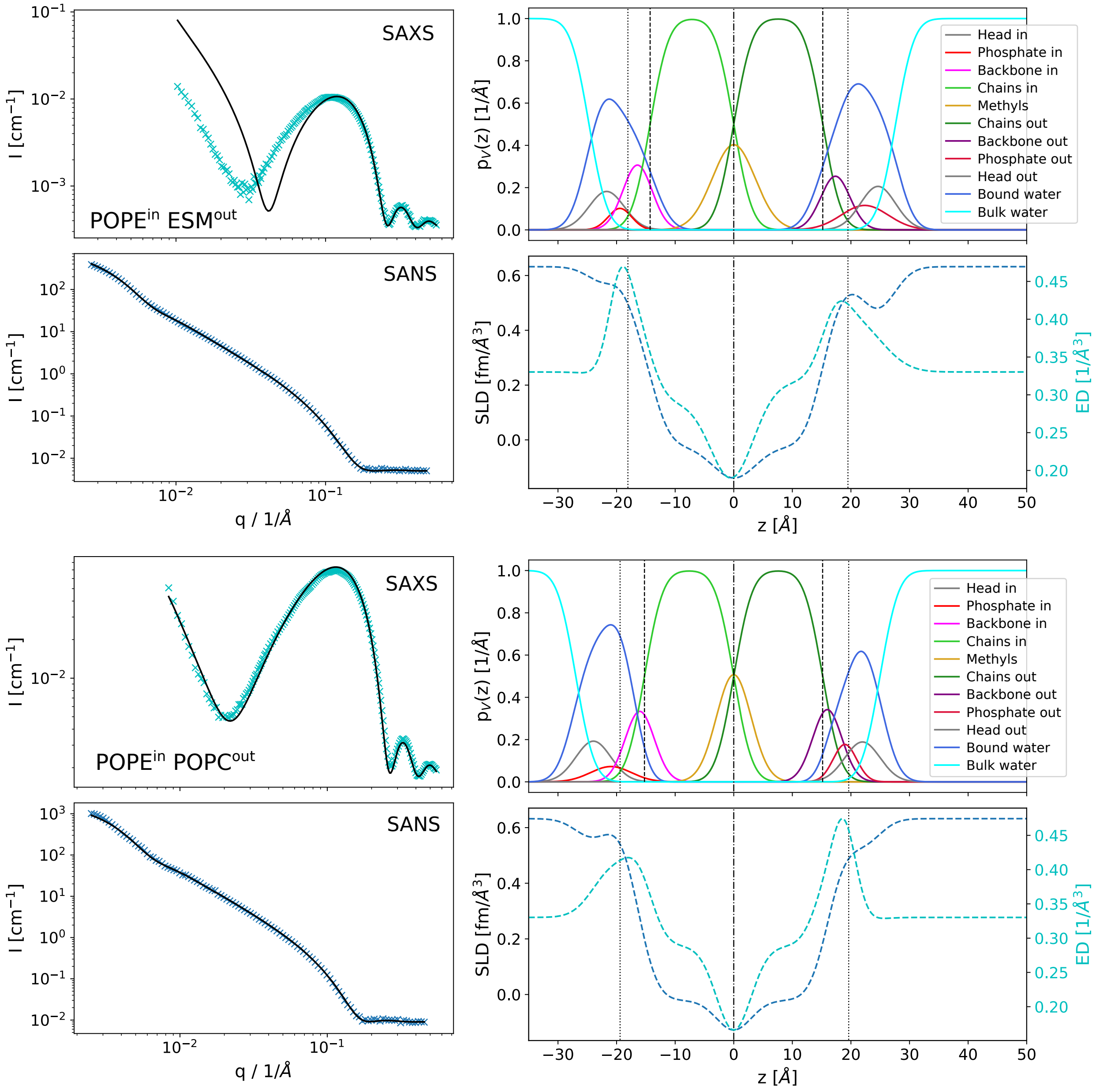

Figure S6: SAXS and SANS data with fits (black lines); SDP volume probability, electron density and neutron scattering length density profiles for the systems POPE$^{\rm in}$/ESM$^{\rm out}$ and POPE$^{\rm in}$/POPC$^{\rm out}$.

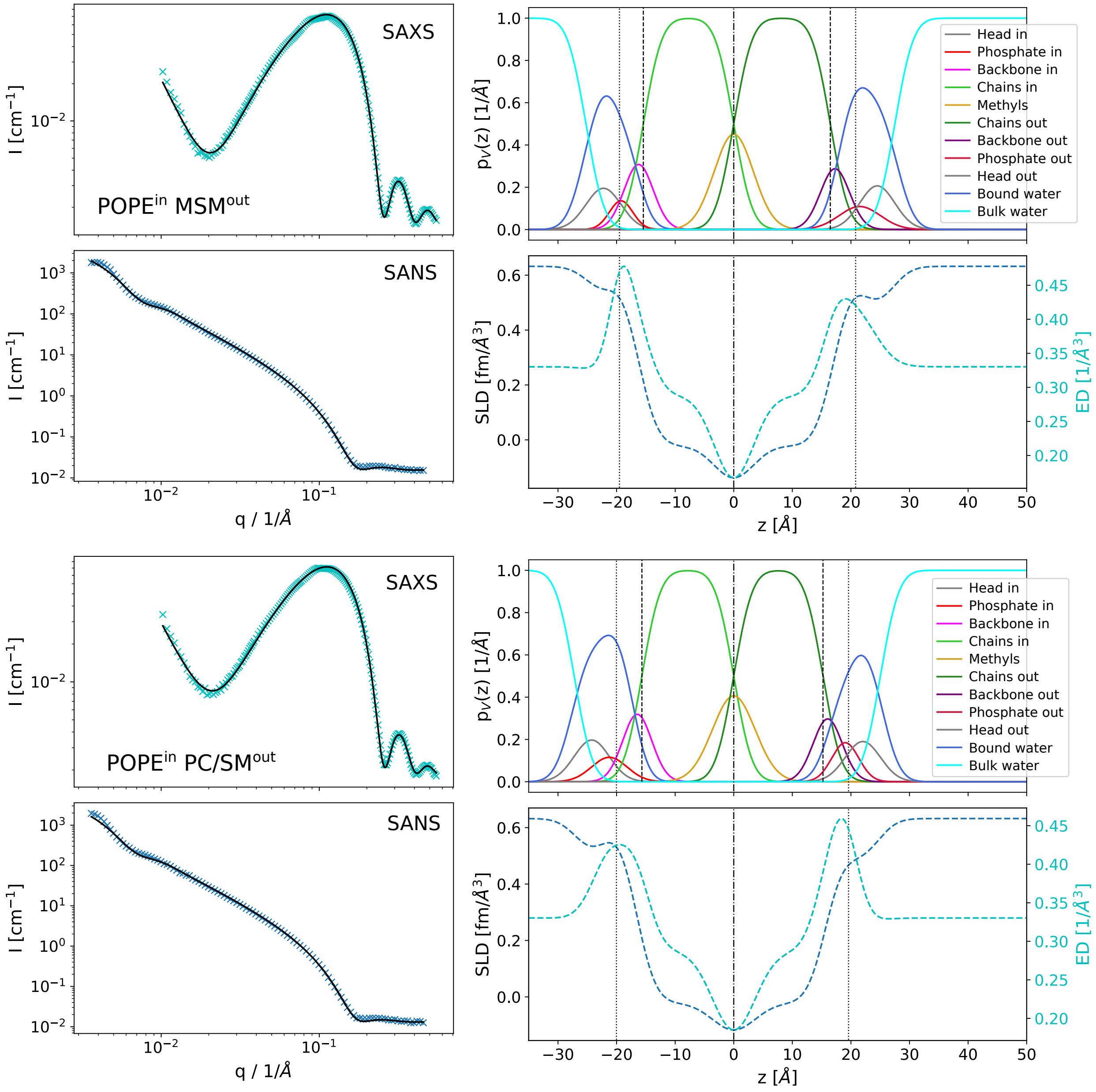

Figure S7: SAXS and SANS data with fits (black lines); SDP volume probability, electron density and neutron scattering length density profiles for the systems POPE^{in}/MSM^{out} and POPE^{in}/(PC/SM)^{out}.

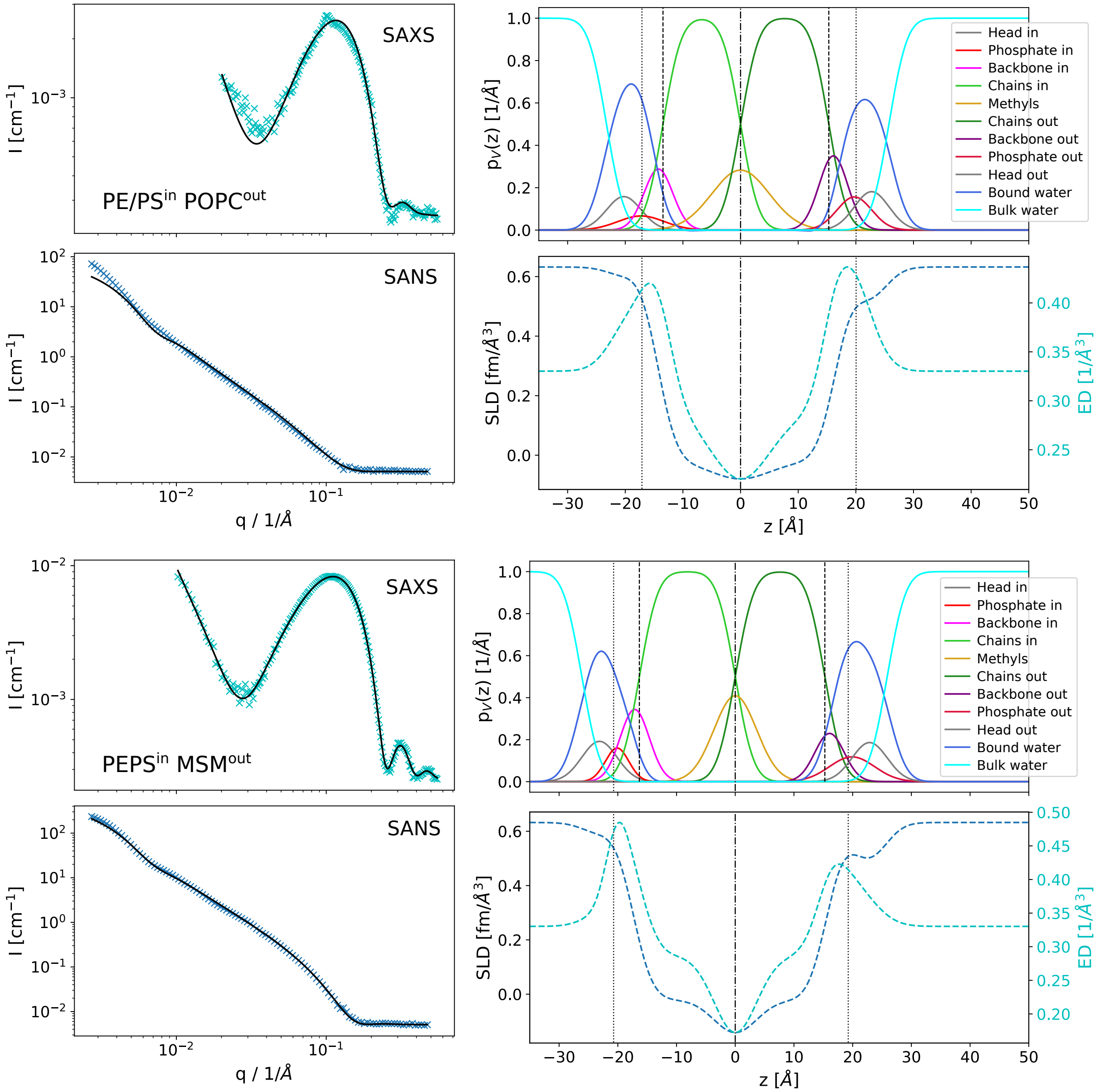

Figure S8: SAXS and SANS data with fits (black lines); SDP volume probability, electron density and neutron scattering length density profiles for the systems $(PE/PS)^{in}/POPC^{out}$ and $(PE/PS)^{in}/MSM^{out}$. SAXS data in the upper panel show a small bragg peak, which is probably connected to a small contamination of multilamellar donor vesicles in the sample. This could be also responsable for the disagreement between data and model in low-q SANS. We expect a higher experimental error for this sample.

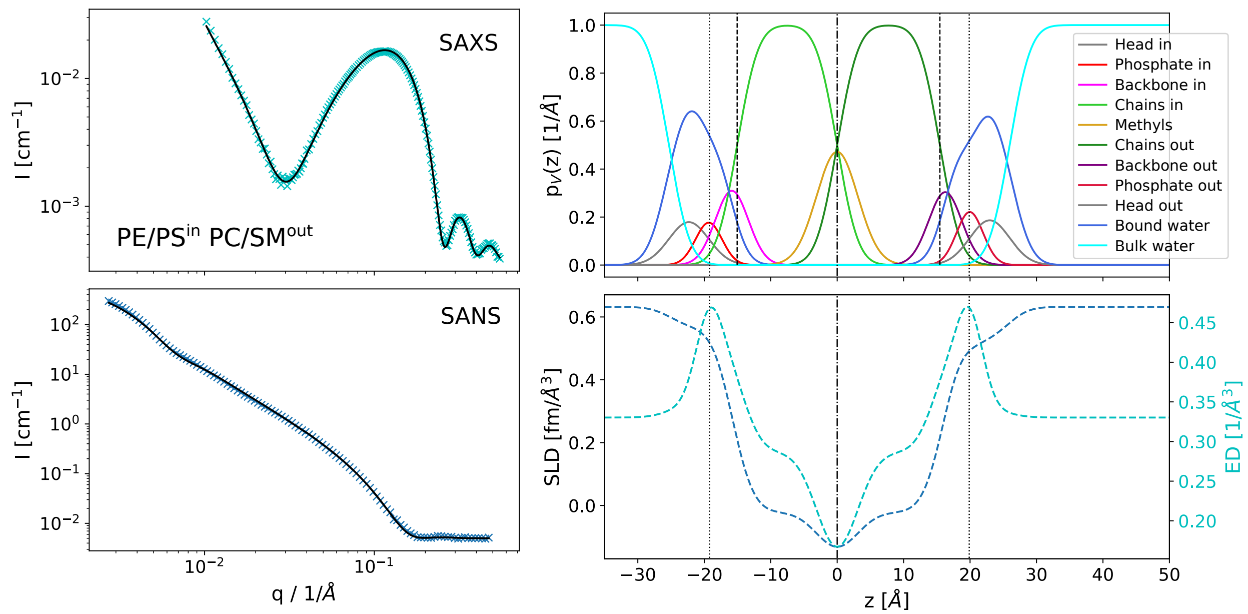

Figure S9: SAXS and SANS data with fits (black lines); SDP volume probability, electron density and neutron scattering length density profiles for the system (PE/PS)^{in}/(PC/SM)^{out}.

8 SAS-model parameters of reference LUVs

Table S6: SAS-fitting parameters of symmetric reference LUVs for POPE/POPG acceptor vesicles.
Inner leaflet samples (in): 90% POPE/POPG (9:1 mol/mol) and 10% POPC, MSM, or POPC/MSM (1:1 mol/mol).
Outer leaflet samples (out): 30% POPE/POPG (9:1 mol/mol) and 70% POPC, MSM, or POPC/MSM (1:1 mol/mol).
Scrambled vesicles (scram): same composition as aLUVs, see Tab. S5.

		POPC			MSM			PC/SM		
	ϵ [%]	in	out	scram	in	out	scram	in	out	scram
V_L^* [Å^3]		1202.1	1252.0	1212.94	1208.1	1293.56	1240.85	1205.11	1272.77	1247.96
V_H^* [Å^3]		257.4	304.48	267.63	252.0	266.68	257.65	254.74	285.58	274.27
r_{BB}^*		0.50	0.46	0.49	0.49	0.38	0.45	0.5	0.42	0.45
r_P^*		0.16	0.25	0.18	0.16	0.27	0.2	0.16	0.26	0.22
r^*		2.09	2.09	2.09	2.09	2	2.09	2.09	2.09	2.09
r_{12}^*		0.8	0.8	0.8	0.8	1	0.8	0.8	0.8	0.8
D_B [Å]	3	39.6	38.9	37.37	40.6	43.13	38.87	39.92	41.28	38.36
D_{HH} [Å]	3	37.2	35.6	33.84	37.0	39.44	35.59	36.3	37.53	35.31
$2D_C$ [Å]	3	31.1	29.4	29.12	32.2	34.23	30.79	31.47	32.01	29.92
D_{H1} [Å]	20	3.1	3.1	2.36	2.4	2.61	2.4	2.41	2.76	2.7
A [Å^2]	2	60.8	64.5	64.93	59.5	60	63.86	60.39	61.68	65.08
z_{BB} [Å]	8	17.9	17.1	15.65	17.7	18.24	16.53	17.13	18.04	16.63
σ_{BB} [Å]	20	2.5	2.5	2.5	2.5	2.5	2.5	2.5	2.5	2.5
z_P [Å]	8	19.1	18.2	17.66	18.9	20.83	18.81	19.15	19.21	18.21
σ_P [Å]	20	3.0	3.2	3.2	3.0	3	3.3	3.5	3.01	3.05
z_h [Å]	3	22.0	21.5	19.95	23	23.72	21.37	23.06	21.1	20.02
$\sigma_h^\dagger$ [Å]		2.8	2.8	2.8	2.8	2.8	2.8	2.8	2.8	2.8
σ_{HC} [Å]		2.5	2.5	2.5	2.5	2.5	2.5	2.5	2.5	2.5
σ_T [Å]	5	2.4	2.4	2.79	2.4	4	3.76	3.51	2.7	4
σ_{poly} [%]	6	4.8	3.22	5.96	5.4	6.34	4.82	4.31	3.66	7.5
$V_{W,bound}$ [Å^3]	6	29.8	29.9	28.83	29.5	29.8	29.01	29.75	29.87	28.47
n_W	6	10.0	10.3	8.73	10.8	9.83	10	11.77	6.66	7.84
R_m [Å]	10	370.1	370.2	430.7	372.5	470.2	408.6	356.2	391.4	390.6
σ_R [Å]	10	103.9	123.7	171.7	102.3	127.7	182.9	91.67	106.7	188.3

Table S7: SAS-fitting parameters of symmetric reference LUVs for POPE/POPS acceptor vesicles.
Inner leaflet samples (in): 90% POPE/POPS (7:3 mol/mol) and 10% POPC, MSM, or POPC/MSM (1:1 mol/mol).
Outer leaflet samples (out): 30% POPE/POPS (7:3 mol/mol) and 70% POPC, MSM, or POPC/MSM (1:1 mol/mol).
Scrambled vesicles (scram): same composition as aLUVs, see Tab. S5.

		POPC			MSM			PC/SM		
	ϵ [%]	in	out	scram	in	out	scram	in	out	scram
V_L^* [Å^3]		1207.1	1253.6	1221.8	1213.03	1295.21	1255.49	1210.06	1274.42	1240.09
V_H^* [Å^3]		262.2	306.07	276.1	256.81	268.27	262.73	259.51	287.17	272.42
r_{BB}^*		0.49	0.46	0.48	0.48	0.37	0.42	0.48	0.41	0.45
r_P^*		0.19	0.26	0.21	0.19	0.28	0.24	0.19	0.27	0.23
r^*		2.09	2.09	2.09	2.09	2	2.09	2.09	2.09	2.09
r_{12}^*		0.8	0.8	0.8	0.8	1	0.8	0.8	0.8	0.8
D_B [Å]	3	37.8	38.2	37.2	38.22	42.65	39.08	38.06	40.22	37.09
D_{HH} [Å]	3	37.0	35.3	32.7	36.85	38.99	38.93	36.28	37.2	36.53
$2D_C$ [Å]	3	29.6	28.8	28.8	30.12	33.81	30.89	29.89	31.15	28.93
D_{H1} [Å]	20	3.7	3.2	2.0	3.37	2.59	4.02	3.19	3.03	3.8
A [Å^2]	2	63.8	65.7	65.8	63.49	60.75	64.27	63.61	63.39	66.89
z_{BB} [Å]	8	16.3	16.1	16.0	17.22	18.53	19.39	16.8	17.84	17.31
σ_{BB} [Å]	20	2.5	2.5	2.5	2.5	2.5	2.86	2.5	2.5	2.5
z_P [Å]	8	19.1	18.3	16.0	19.92	20.34	19.39	19.59	19.1	19.42
σ_P [Å]	20	2.2	2.8	2.9	3.5	3.29	2.2	3.5	3.27	3.5
z_h [Å]	3	24.1	23.3	21	19.92	21.77	24.39	19.59	19.47	19.42
$\sigma_h^\dagger$ [Å]		2.8	2.8	2.8	2.8	2.8	2.8	2.8	2.8	2.8
σ_{HC} [Å]		2.5	2.5	2.5	2.5	2.5	2.5	2.5	2.5	2.5
σ_T [Å]	5	3.5	3.1	4.0	3.32	3.89	2.4	3.26	3.19	4
σ_{poly} [%]	6	7.3	6.63	10.0	5.26	4.81	9.46	6.08	5.58	6.92
$V_{W,bound}$ [Å^3]	6	30.0	29.7	30.3	30.03	29.78	30.01	28.97	29.26	29.41
n_W	6	16.9	15.2	11.4	7.58	6.53	16.25	7.07	4.54	8.14
R_m [Å]	10	381	392.6	469.1	409.5	495.7	607.7	430.9	451.9	500.7
σ_R [Å]	10	109.2	133.5	154.4	131.7	167.1	151.9	140.6	133.1	128.7

Table S8: SAS-fitting parameters of symmetric reference LUVs for POPE/POPG acceptor vesicles.
Inner leaflet samples (in): 90% POPE/POPG (9:1 mol/mol) and 10% ESM.
Outer leaflet samples (out): 30% POPE/POPG (9:1 mol/mol) and 70% ESM.
Scrambled vesicles (scram): same composition as aLUVs, see Tab. S5.

		ESM		
	ϵ [%]	in	out	scram
V_L^* [Å^3]		1196.5	1212.2	1181.0
V_H^* [Å^3]		252.0	266.68	259.1
r_{BB}^*		0.49	0.38	0.44
r_P^*		0.16	0.27	0.21
r^*		2.09	2.09	2.09
r_{12}^*		0.8	0.8	0.8
D_B [Å]	3	38.0	41.1	38.2
D_{HH} [Å]	3	36.9	37.0	34.9
$2D_C$ [Å]	3	30.0	32.0	29.8
D_{H1} [Å]	20	3.4	2.5	2.6
A [Å^2]	2	63.0	59.1	61.8
z_{BB} [Å]	8	17.6	17.6	15.7
σ_{BB} [Å]	20	2.5	2.5	2.5
z_P [Å]	8	19.4	18.7	20.2
σ_P [Å]	20	3.5	2.7	3.5
z_h [Å]	3	19.4	22.9	21
$\sigma_h^\dagger$ [Å]		2.8	2.8	2.8
σ_{HC} [Å]		2.5	2.5	2.5
σ_T [Å]	5	3.1	2.4	4.0
σ_{poly} [%]	6	5.3	4.95	8.5
$V_{W,bound}$ [Å^3]	6	27.7	27.6	30.3
n_W	6	6.7	10.0	8.8
R_m [Å]	10	393.1	455	531.6
σ_R [Å]	10	114.1	132.9	138.9

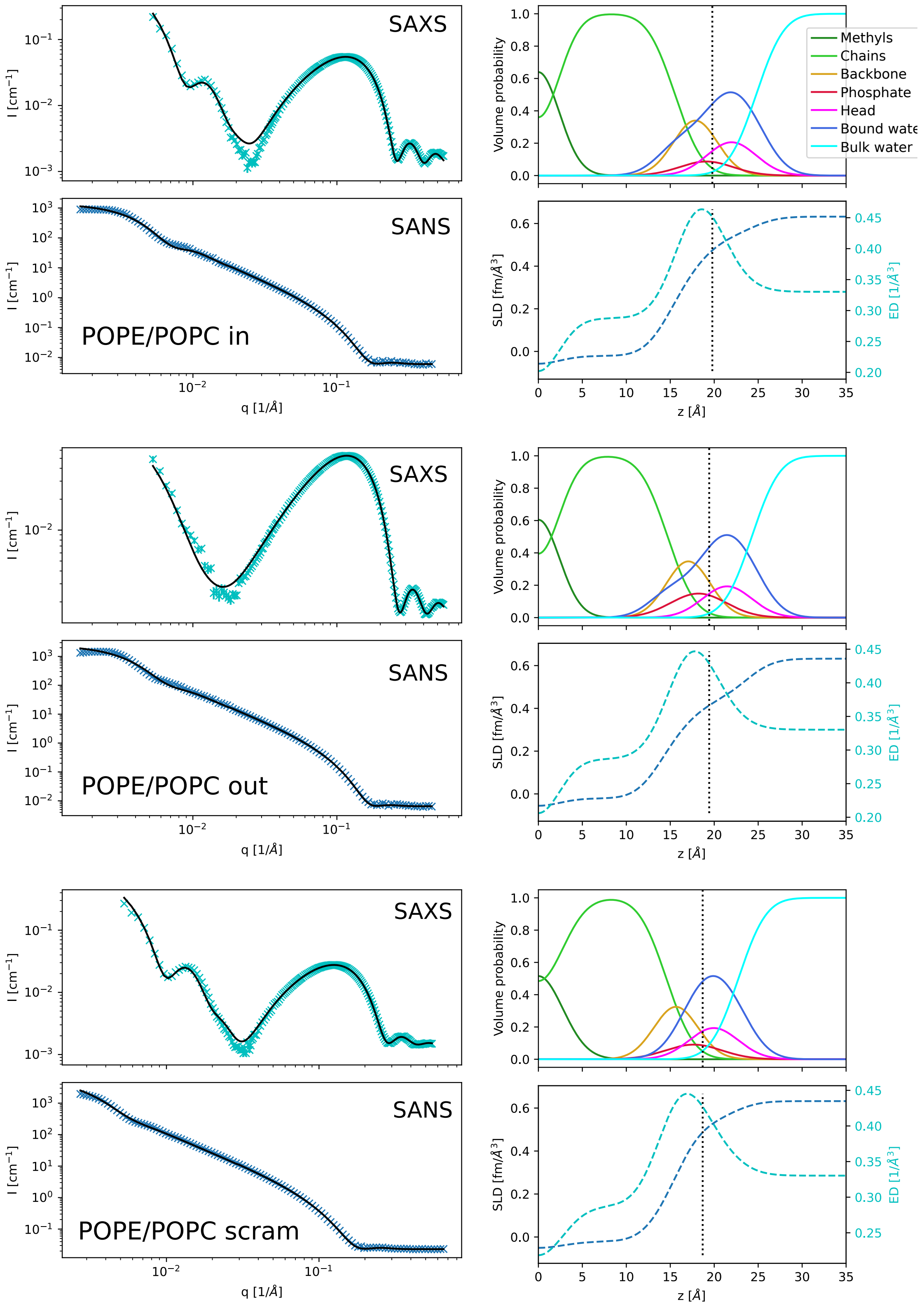

Figure S10: SAXS and SANS data with fits (black lines); SDP volume probability, electron density and neutron scattering length density profiles for POPE/POPC inner/outer leaflet symmetric mimics, as well as the scrambled sample.

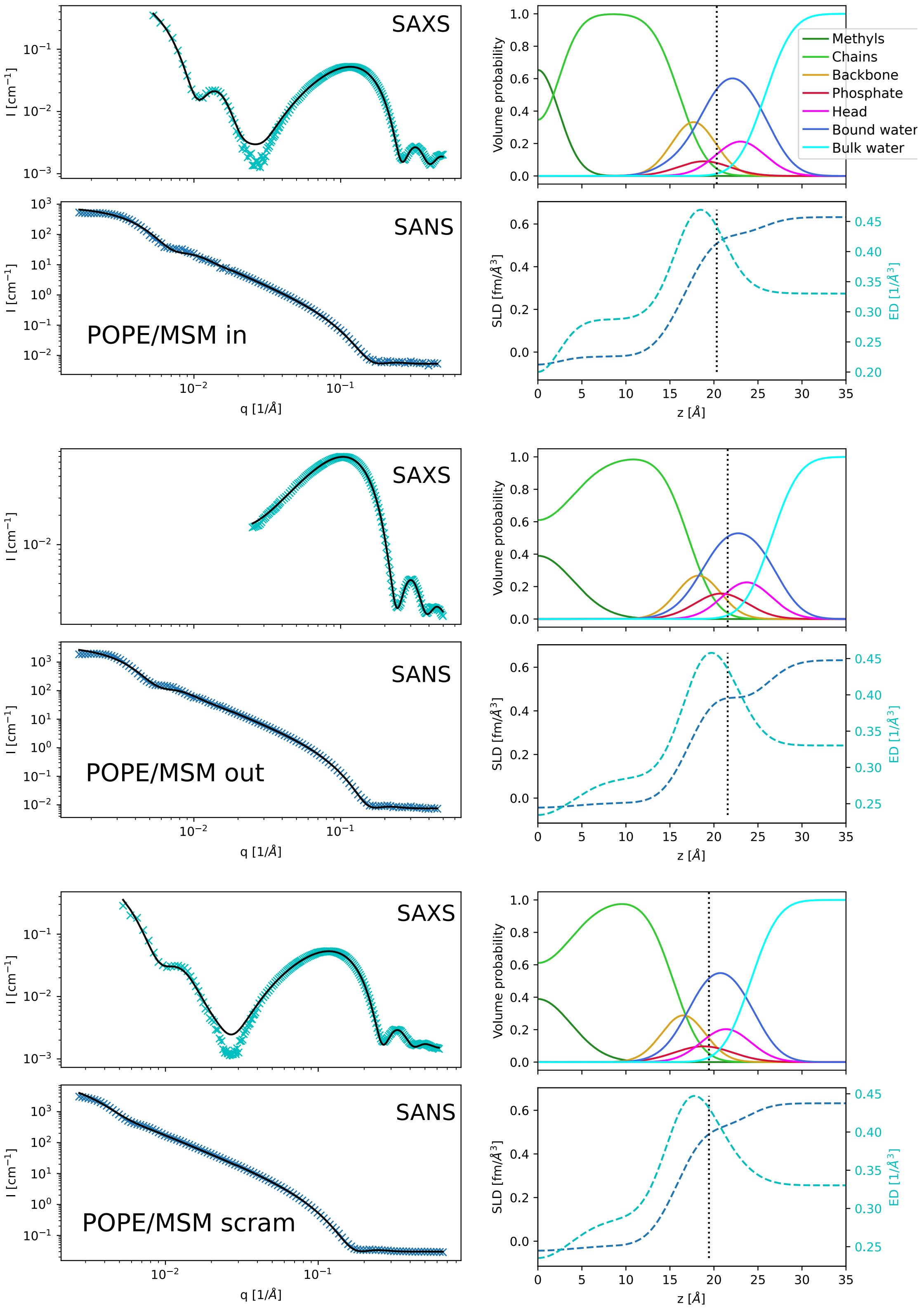

Figure S11: SAXS and SANS data with fits (black lines); SDP volume probability, electron density and neutron scattering length density profiles for POPE/MSM inner/outer leaflet symmetric mimics, as well as the scrambled sample.

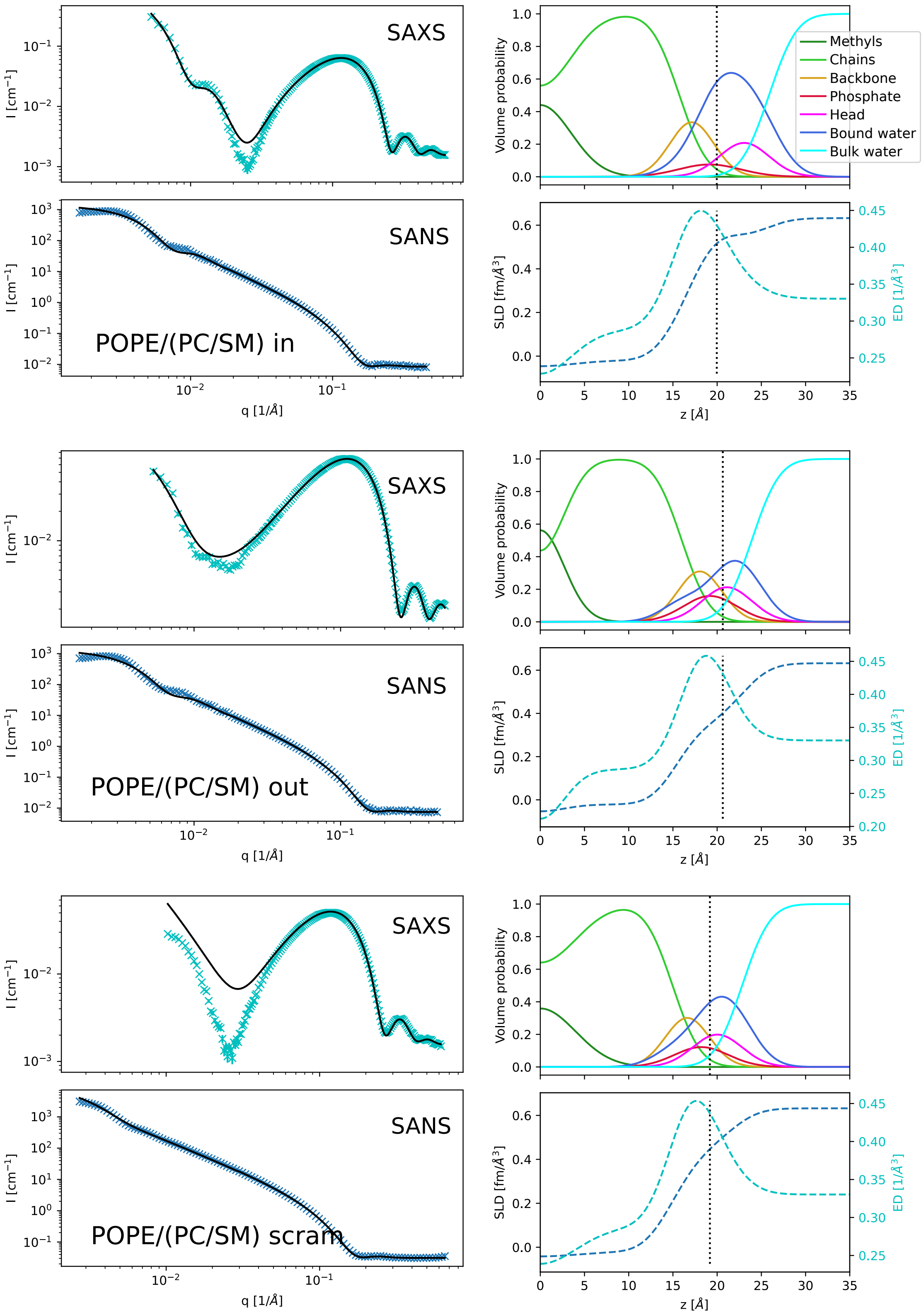

Figure S12: SAXS and SANS data with fits (black lines); SDP volume probability, electron density and neutron scattering length density profiles for POPE/(PC/SM) inner/outer leaflet symmetric mimics, as well as the scrambled sample.

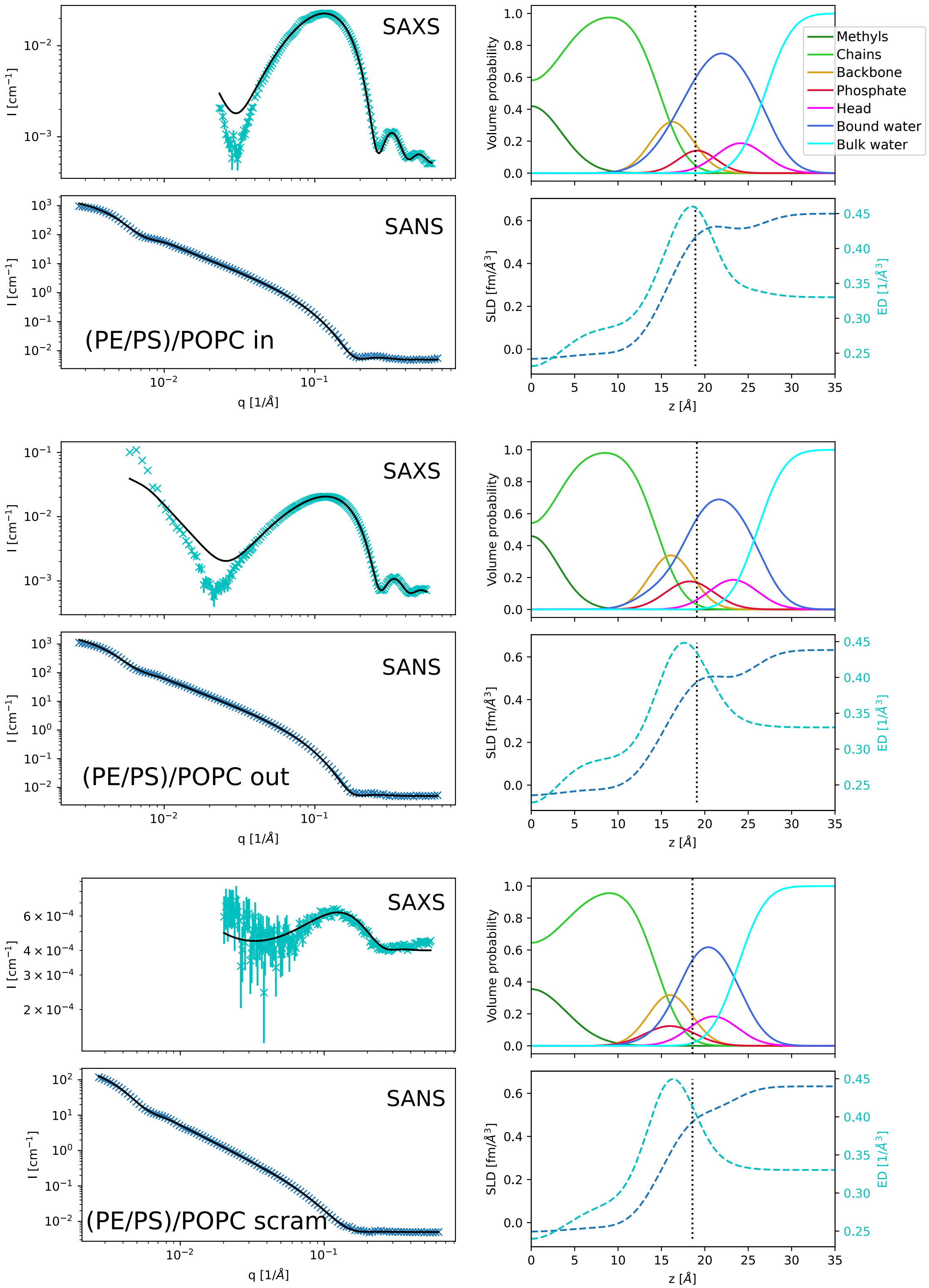

Figure S13: SAXS and SANS data with fits (black lines); SDP volume probability, electron density and neutron scattering length density profiles for (PE/PS)/POPC inner/outer leaflet symmetric mimics, as well as the scrambled sample.

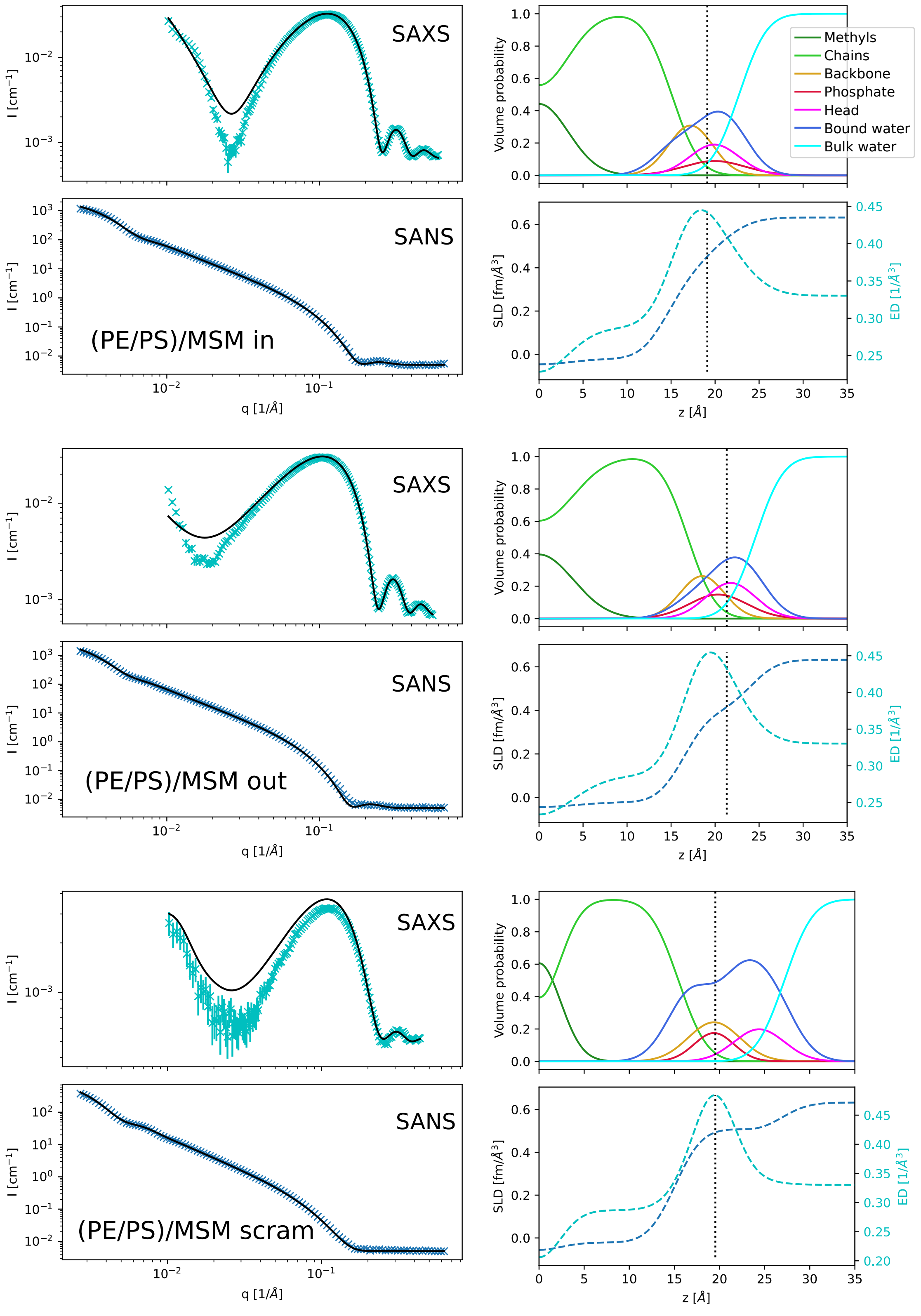

Figure S14: SAXS and SANS data with fits (black lines); SDP volume probability, electron density and neutron scattering length density profiles for (PE/PS)/MSM inner/outer leaflet symmetric mimics, as well as the scrambled sample.

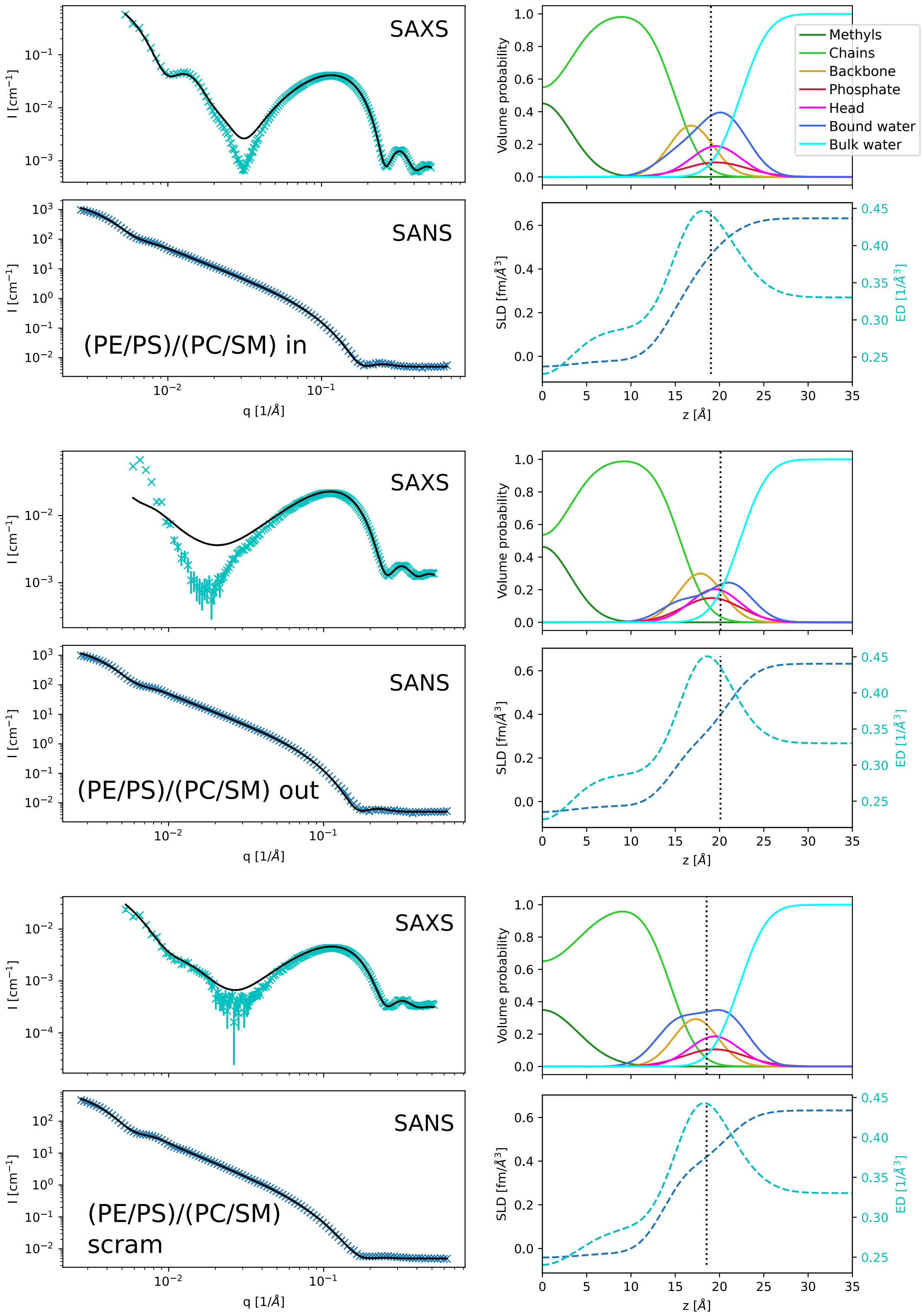

Figure S15: SAXS and SANS data with fits (black lines); SDP volume probability, electron density and neutron scattering length density profiles for (PE/PS)/(PC/SM) inner/outer leaflet symmetric mimics, as well as the scrambled sample.

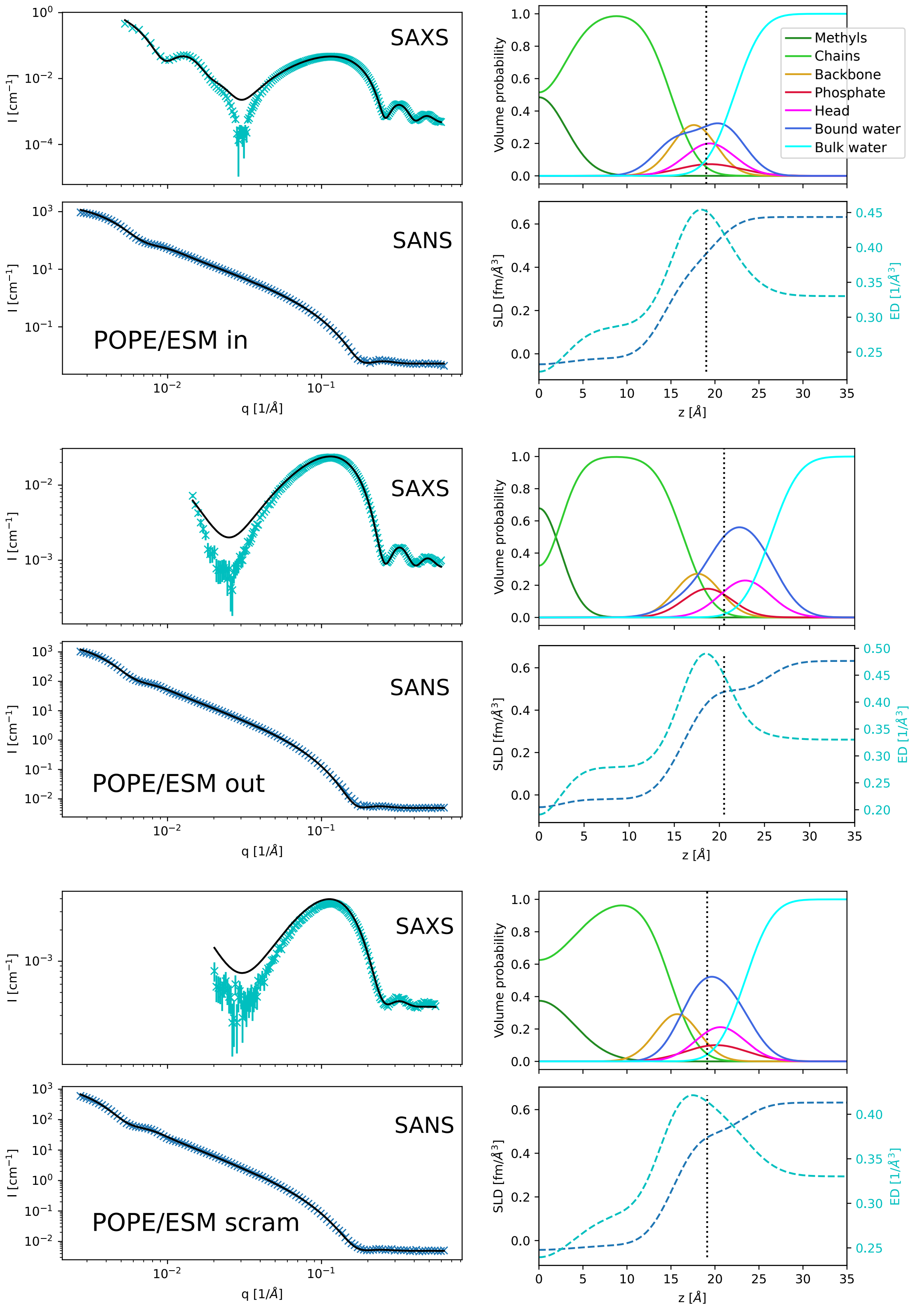

Figure S16: SAXS and SANS data with fits (black lines); SDP volume probability, electron density and neutron scattering length density profiles for POPE/ESM inner/outer leaflet symmetric mimics, as well as the scrambled sample.

References

[1] Ali Imran, Dumitru Popescu, and Liviu Movileanu. Cyclic activity of an osmotically stressed liposome in a finite hypotonic environment. Langmuir, 36(13):3659–3666, 2020.

[2] A Srinivas Reddy, Dora Toledo Warshaviak, and Mirianas Chachisvilis. Effect of membrane tension on the physical properties of dopc lipid bilayer membrane. Biochimica et Biophysica Acta (BBA)-Biomembranes, 1818(9):2271–2281, 2012.

[3] Rubèn Serral Gracià, Natalya Bezlyepkina, Roland L. Knorr, Reinhard Lipowsky, and Rumiana Dimova. Effect of cholesterol on the rigidity of saturated and unsaturated membranes: fluctuation and electrodeformation analysis of giant vesicles. Soft Matter, 6(7):1472–1482, 2010.

[4] W. Rawicz, K.C. Olbrich, T. McIntosh, D. Needham, and E. Evans. Effect of chain length and unsaturation on elasticity of lipid bilayers. Biophysical Journal, 79:328–339, 7 2000.

[5] Moritz P. K. Frewein, Paulina Piller, Enrico F. Semeraro, Krishna C. Batchu, Frederick A. Heberle, Haden L. Scott, Yuri Gerelli, Lionel Porcar, and Georg Pabst. Interdigitation-induced order and disorder in asymmetric membranes. The Journal of Membrane Biology, 4 2022.

[6] Moritz P. K. Frewein, Milka Doktorova, Frederick A. Heberle, Haden L. Scott, Enrico F. Semeraro, Lionel Porcar, and Georg Pabst. Structure and interdigitation of chain-asymmetric phosphatidylcholines and milk sphingomyelin in the fluid phase. Symmetry, 13:1441, 8 2021.

[7] Norbert Kučerka, Jeremy Pencer, Jonathan N. Sachs, John F. Nagle, and John Katsaras. Curvature effect on the structure of phospholipid bilayers. Langmuir, 23(3):1292–1299, 2007.
